# Synthesis and Cytotoxic Evaluation of Alkoxylated Chalcones

**DOI:** 10.3390/molecules191117256

**Published:** 2014-10-28

**Authors:** Xiao-Guang Bai, Chang-Liang Xu, Shuang-Shuang Zhao, Hong-Wei He, Yu-Cheng Wang, Ju-Xian Wang

**Affiliations:** Institute of Medicinal Biotechnology, Chinese Academy of Medical Sciences and Peking Union Medical College, Beijing 100050, China; E-Mails: baixiaoguang130@126.com (X.-G.B.); clxu1986@163.com (C.-L.X.); zhshsh1112@163.com (S.-S.Z.); hehwei@163.com (H.W.H.); wyc9999@126.com (Y.-C.W.)

**Keywords:** alkoxylated chalcones, synthesis, cytotoxicity

## Abstract

A series of chalcones **a1**–**20** bearing a 4-OMe groups on the A-ring were initially synthesized and their anticancer activities towards HepG2 cells evaluated. Subsequently, a series of chalcones **b1**–**42** bearing methoxy groups at the 2' and 6'-positions of the B-ring were synthesized and their anticancer activities towards five human cancer cell lines (HepG2, HeLa, MCF-7, A549 and SW1990) and two non-tumoral human cell lines evaluated. The results showed that six compounds (**b6**, **b8**, **b11**, **b16**, **b18**, **b22**, **b23** and **b29**) displayed promising activities, with compounds **b22** and **b29** in particular showing higher levels of activity than etoposide against all five cancer cell lines. Compound **b29** showed a promising SI value compared with both HMLE and L02 (2.1–6.5 fold in HMLE and > 33 > 103.1 fold in L02, respectively).

## 1. Introduction

**Figure 1:**
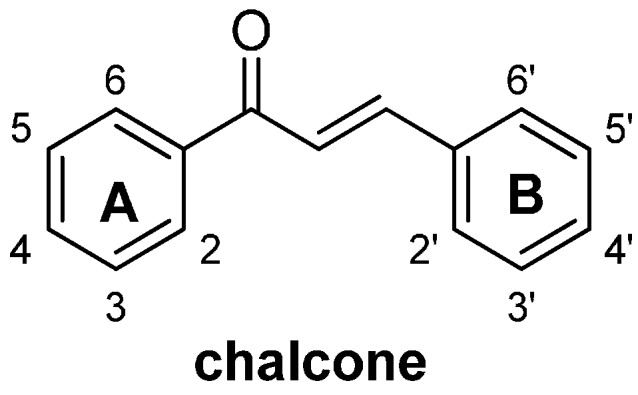
General structure of chalcones.

Chalcones (1,3-diaryl-2-propen-1-ones, Figure 1) are naturally occurring compounds that are widely distributed in a variety of plant species. Compounds belonging to this structural class have been identified as both intermediates and end products in the biosynthesis of flavonoids, and make significant contributions to the medicinal value of herbs. The ease of preparation of these compounds, as well as their potential for oral administration and safety, make them good candidates for use as therapeutic agents in drug discovery [1]. For these reasons, chalcones have attracted considerable attention from chemists and pharmacologists, and a large number of results have been reported in the literature pertaining to the biological activities of both naturally occurring and synthetic chalcone compounds, including their anti-inflammatory [2,3], antimicrobial [4,5,6], antifungal [7,8], antioxidant [9] and anticancer activities [10,11,12,13,14,15].

The antitumor properties of chalcones have received considerable attention during the last few years, because they operate via a similar mode of action to the structurally related combretastatin [16]. The frequent occurrence of polymethoxyphenyl-type moieties in naturally occurring anticancer agents, such as colchicine and combretastatin, has inspired scientists to investigate the synthesis and biological evaluation of methoxylated chalcones, and the results of these studies have shown that methoxy groups generally make a significant contribution to the cytotoxicity of these compounds [17,18,19].

To develop potential anticancer drugs based on chalcones, as well as improve our understanding of the structure-activity relationships (SARs) of these compounds, we report herein the synthesis of a series of alkoxylated chalcones. The cytotoxic activities of these 62 compounds have also been evaluated against a panel of five cancer cell lines and two normal human cell lines, including HepG2, HeLa, MCF-7, A549, SW1990, HMLE and L02 cells.

## 2. Results and Discussion

Compounds **a1**–**20** bearing a 4-OMe on their A-ring (*i.e*., their 1-phenyl moiety) were initially synthesized and evaluated in terms of their activity towards human liver carcinoma HepG2 cells using a SRB assay. This evaluation was conducted to develop a better understanding of the structural-activity relationship (SAR) of the B-ring of these compounds and the activity results (percentage rates at concentration of 10 μM) are shown in Table 1.

**Table 1:** Inhibitory activities of the 4-methoxyl chalcones **a1**–**20** against HepG2 cells (10 μM). 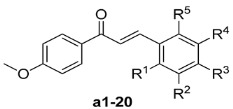

Compound	R^1^	R^2^	R^3^	R^4^	R^5^	Inhibition (%) **
**a1**	H	H	H	H	H	42.4 ± 2.0
**a2**	H	H	Br	H	H	37.6 ± 4.4
**a3**	F	H	Br	H	H	41.8 ± 5.9
**a4** *	Br	H	F	H	H	41.2 ± 4.6
**a5** *	Cl	H	Br	H	H	9.0 ± 5.6
**a6**	H	H	NO_2_	H	H	9.8 ± 5.0
**a7**	H	H	COOH	H	H	32.7 ± 3.0
**a8**	OCH_3_	H	H	H	H	61.4 ± 3.4
**a9**	H	OCH_3_	H	H	H	59.0 ± 2.9
**a10**	H	H	OCH_3_	H	H	27.7 ± 17.7
**a11**	H	H	OCH_2_CH_3_	H	H	13.1 ± 8.4
**a12**	OCH_3_	OCH_3_	H	H	H	66.3 ± 4.2
**a13**	OCH_3_	H	H	OCH_3_	H	77.0 ± 2.2
**a14** *	OCH_3_	H	H	H	OCH_3_	78.1 ± 4.7
**a15**	H	OCH_3_	OCH_3_	H	H	65.0 ± 17.6
**a16**	H	OCH_3_	H	OCH_3_	H	70.6 ± 2.0
**a17**	H	OCH_2_Ph	OCH_3_	H	H	44.1 ± 6.2
**a18**	OCH_3_	OCH_3_	OCH_3_	H	H	31.8 ± 7.3
**a19**	OCH_3_	H	OCH_3_	OCH_3_	H	38.1 ± 10.3
**a20**	H	OCH_3_	OCH_3_	OCH_3_	H	71.1 ± 1.3
**Etoposide**						63.9 ± 0.1

*: Novel compound; **: The results are shown as the average percentage of the individual mean (±SD, standard deviation) for three independent experiments.

The introduction of halogen atoms (*i.e*., compounds **a2**–**5**), a NO_2_ substituent (**a6**) and a COOH group (**a7**) on the B-ring led to a decrease in the activity of these compounds compared with the parent compound **a1**.Contrary to **a2**–**7**, compounds **a8**–**20** which were substituted with electron donating groups (*i.e*., OMe, OEt and OBn), generally exhibited moderate to high levels of inhibition. A single OMe group substituted at the 2'-position (**a8**) or 3'-position (**a9**) led to much better inhibition than that of the 4'-position (**a10**). A comparison of **a10** *vs.* **a11** and **a15** *vs.* **a17** revealed that bulky substituents seemed to be poorly tolerated at the 3' and 4'-position. Interestingly, the substitution of the B ring with two OMe groups (compounds **a12**–**16**) led to a general increase in the activity (inhibition 65.0%–78.1%), with bis-substitution at the 2' and 6' positions (**a14**) providing the best results. The trimethoxy- substituted chalcones synthesized in the current study (*i.e*., compound **a18**–**20**) showed weak anti-proliferative activity towards HepG2 cells, except for the 3',4',5'-trimethoxy compound **a20**.

It is possible to draw some conclusions from the results in Table 1, including: (1) the introduction of electron donating groups to the B-ring generally made a positive contribution to the cytotoxicity, whereas electron withdrawing groups tended to have an adverse impact on the cytotoxicity; (2) the position and size of the substituents might have a specific impact on the activity of these compounds; and (3) the presence of two OMe moiety on the B-ring led to an remarkable increase in the cytotoxic activity.

With a aim of identifying potential anticancer agents with enhanced levels of activity, as well as verifying the conclusions provided above, we took **a14** as a starting compound, and then designed and synthesized a series of chalcones **b1**–**42** bearing methoxy groups at the 2' and 6'-positions of the B-ring, whilst varying the substituents on the A-ring. The cytotoxicities of the resulting chalcones were evaluated *in vitro* against five human cancer cell lines and other two non-tumoral cell lines, including HepG2, HeLa, MCF-7, A549, SW1990, HMLE and L02, and the results are shown in Table 2.

Compounds bearing electron donating groups (*i.e*., Me, OMe, OEt, OBn, OCH_2_O, CH_2_CH_2_O, Ph, NH_2_ and OH) generally exhibited higher levels of anticancer activity than those with electron withdrawing groups (*i.e*., F, Cl, Br, NO_2_, CN and CF_3_). Furthermore, chalcones substituted with alkoxyl groups exhibited greater cytotoxic activities than those substituted with methyl, amino or hydroxyl groups (e.g., the IC_50_ values of **b6**–**11** were lower than those of **b2**–**5** and **b23**–**27)**. A comparison of **b2**–**4**, **b6**–**13** and **b24**–**26** revealed that both the position and the size of the substituents had a significant impact on the cytotoxic activities of the compounds, with the position effect appearing to be of the order *o* > *m* > *p* (confirmed by **b30**–**33**), and the size contribution following the order OMe > OEt > OBn (**b11** is an exception to this order).

A review of compounds **b30** to **b42** revealed that those with electron withdrawing groups exhibited weak cytotoxic activity, and that the cytotoxic activities of these compounds were related to the electrophilic nature of their substituents. For example, nearly all of the IC_50_ values for compounds **b30** and **b39**–**42**, which had a CN, NO_2_ or CF_3_ group on their A-ring, were greater than 30 µM.

It’s noteworthy that compound **b6**, **b8**, **b11**, **b16**, **b18**, **b22**, **b23** and **b29** exhibited the most outstanding activities among all the compounds tested in this study, with most of their *in vitro* IC_50_ values against the five human cancer lines being less than 10 µM. Compound **b22** was 20.4- and 23.6-fold as potent than etoposide against HepG2 and SW1990 cells, respectively, and compound **b29** was 11.5-fold more potent than etoposide against A549 cells. These two compounds displayed the best activity and therefore are deserving of further investigation.

All compounds were also evaluated for their cytotoxic activity against two non-tumoral human cell lines named human mammary epithelial cell (HMLE) and L02 (a human liver cell line) in Table 2 and SI values between the CC_50_ in non-tumoral cell lines and IC_50_ in tumoral cells calculated. The data indicate that about one third of the compounds (**b8**, **b11**–**13**, **b16**, **b18**–**24**, **b28**–**29**, **b31**–**32**, **b34**, **b36**–**38**) exhibit more than 2-fold better SI between the CC_50_ in L02 and IC_50_ in tumoral cells, which is similar to the value of etoposide. Among them, compounds **b11**, **b16**, **b22**, **b23** exhibited over 5-fold better SI toward L02. However, most compounds’ SI values between the CC_50_ in HMLE and IC_50_ in tumoral cells are unsatisfactory, except for **b11**, **b27**, **b29** and **b31** (2–7 fold), which is also similar to etoposide. Fortunately, compound b11, and especially b29, showed promising SI values compared with both HMLE and L02 (2.1–6.5 fold in HMLE and >33–>103.1 fold, respectively).

**Table 2:** Cytotoxicities of the 2',6'-dimethoxyl chalcones **b1**–**b42** against five human cancer cell lines and two non-tumoral cell lines. 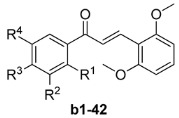

Compound	R^1^	R^2^	R^3^	R^4^	Human Cancer Cell Lines, IC_50_ (µM) & SI										Normal Human Cell Lines, CC_50_ (µM)*^b^*	
					HepG2		HeLa		MCF7		A549		SW1990		HMLE	L02
					IC_50_ *^a^*		IC_50_ *^a^*		IC_50_ *^a^*		IC_50_ *^a^*		IC_50_ *^a^*			
					SI *^c^*	SI *^d^*	SI *^e^*	SI *^f^*	SI *^g^*	SI *^h^*	SI *^i^*	SI *^j^*	SI *^k^*	SI *^l^*		
**b1 ^#^**	H	H	H	H	20.98 ± 1.75		>30		28.18 ± 0.04		>30		>30		9.47 ± 0.39	33.340.39e
					0.5	1.6	<0.3	<1.1	0.3	1.2	<0.3	<1.1	<0.3	<1.1		
**b2**	CH_3_	H	H	H	14.07 ± 1.84		18.40 ± 0.75		19.08 ± 2.11		25.47 ± 4.14		>30		14.98 ± 0.78	35.87 ± 3.40
					1.1	2.5	0.8	1.9	0.8	1.9	0.6	1.4	<0.5	<1.2		
**b3**	H	CH_3_	H	H	12.81 ± 0.50		13.28 ± 2.14		26.78 ± 4.26		20.69 ± 0.59		25.23 ± 2.00		16.20 ± 0.19	33.26 ± 1.61
					1.3	2.6	1.2	2.5	0.6	1.2	0.8	1.6	0.6	1.3		
**b4**	H	H	CH_3_	H	19.51 ± 1.28		18.60 ± 0.36		25.44 ± 0.15		22.07 ± 0.60		>30		13.77 ± 1.69	59.59 ± 5.56
					0.7	3.1	0.7	3.2	0.5	2.3	0.6	2.7	<0.5	<2.0		
**b5**	H	CH_3_	CH_3_	H	26.79 ± 0.30		20.33 ± 1.01		30.31 ± 0.29		>30		>30		15.66 ± 0.76	25.15 ± 2.71
					0.6	0.9	0.8	1.2	0.5	0.8	<0.5	<0.8	<0.5	<0.8		
**b6 ^#^**	OCH_3_	H	H	H	2.05 ± 0.22		5.00 ± 0.03		5.60 ± 0.17		6.35 ± 0.32		10.02 ± 0.14		4.01 ± 0.05	8.78 ± 1.21
					2.0	4.3	0.8	1.8	0.7	1.6	0.6	1.4	0.4	0.9		
**b7**	H	OCH_3_	H	H	6.97 ± 0.55		9.98 ± 0.37		6.37 ± 0.05		14.02 ± 0.19		15.62		8.14 ± 0.53	18.08 ± 5.82
					1.2	2.6	0.8	1.8	1.3	2.8	0.6	1.3	0.5	1.2		
**a16**			OCH_3_		9.20 ± 0.31		12.42 ± 0.34		23.10 ± 0.79		21.18 ± 0.55		17.80 ± 0.07		3.28 ± 0.31	8.87 ± 1.83
					0.4	1.0	0.3	0.7	0.1	0.4	0.2	0.4	0.2	0.5		
**b8**	OCH_2_CH_3_	H	H	H	4.23 ± 0.09		8.72 ± 0.07		8.56 ± 0.17		7.36 ± 0.37		8.20 ± 0.12		0.83 ± 0.21	39.55 ± 7.53
					0.2	9.3	0.1	4.5	0.1	4.6	0.1	5.4	0.1	4.8		
**b9**	H	OCH_2_CH_3_	H	H	7.71 ± 0.21		12.16 ± 0.25		11.59 ± 0.07		13.22 ± 0.43		10.68 ± 0.08		9.58 ± 0.44	17.42 ± 5.96
					1.2	2.3	0.8	1.4	0.8	1.5	0.7	1.3	0.9	1.6		
**b10**	H	H	OCH_2_CH_3_	H	10.92 ± 0.46		14.36 ± 0.15		19.76 ± 0.69		22.72 ± 0.57		19.47 ± 0.23		13.81 ± 3.77	36.66 ± 3.41
					1.3	3.4	1.0	2.6	0.7	1.9	0.6	1.6	0.7	1.9		
**b11**	OCH_2_Ph	H	H	H	2.76 ± 0.04		3.62 ± 0.07		4.15 ± 0.2		3.63 ± 0.13		7.43 ± 0.56		20.99 ± 1.26	38.28 ± 5.63
					7.6	13.9	5.8	10.6	5.1	9.2	5.8	10.5	2.8	5.2		
**b12**	H	OCH_2_Ph	H	H	23.59 ± 0.84		25.59 ± 1.16		29.03 ± 0.09		13.25 ± 0.02		22.30 ± 0.07		31.40 ± 1.12	>100
					1.3	>4.2	1.2	>3.9	1.1	>3.4	2.4	>7.5	1.4	>7.5		
**b13 ^#^**	H	H	OCH_2_Ph	H	21.75 ± 0.62		>30		>30		>30		>30		>100	>100
					>4.6	>4.6	-	-	-	-	-	-	-	-		
**b14**	OCH_2_Ph	H	OCH_3_	H	>30		>30		>30		>30		>30		>100	>100
					-	-	-	-	-	-	-	-	-	-		
**b15**	H	OCH_3_	OCH_2_Ph	H	>30		>30		>30		>30		>30		>100	>100
					-	-	-	-	-	-	-	-	-	-		
**b16**	H	OCH_3_	OCH_2_CH_3_	H	4.14 ± 0.11		5.02 ± 0.09		4.79 ± 0.13		5.78 ± 0.11		7.67 ± 0.04		9.15 ± 1.33	44.70 ± 8.15
					2.2	10.8	1.8	8.9	1.9	9.3	1.6	7.7	1.2	5.8		
**b17**	OCH_2_CH_3_	H	OCH_3_	H	11.97 ± 0.23		8.54 ± 0.14		7.74 ± 0.10		9.33 ± 0.30		10. 80 ± 0.50		9.03 ± 0.80	10.92 ± 0.42
					0.8	0.9	1.1	1.3	1.2	1.4	1.0	1.2	0.8	1.0		
**b18**	OCH_3_	H	H	OCH_3_	1.59 ± 0.15		4.56 ± 0.22		5.60 ± 0.06		4.16 ± 0.72		9.64 ± 0.18		2.48 ± 0.34	16.97 ± 2.73
					1.6	10.7	0.5	3.7	0.4	3.0	0.6	4.1	0.3	1.8		
**b19 ^#^**	H	OCH_3_	OCH_3_	H	7.12 ± 0.67		9.26 ± 0.04		19.67 ± 0.20		13.62 ± 0.85		13.84 ± 0.06		10.53 ± 0.41	39.75 ± 6.32
					1.5	5.6	1.1	4.3	0.5	2.0	0.8	2.9	0.8	2.9		
**b20 ^#^**	H	OCH_2_O		H	17.46 ± 0.54		16.23 ± 0.95		22.54 ± 0.37		19.50 ± 0.90		25.85 ± 0.65		14.21 ± 1.06	60.90 ± 5.61
					0.8	3.5	0.9	3.8	0.6	2.7	0.7	3.1	0.5	2.4		
**b21**	H	CH_2_CH_2_O		H	17.34 ± 0.23		21.57 ± 0.43		22.62 ± 2.10		22.26 ± 0.99		24.37 ± 0.60		14.58 ± 0.81	57.48 ± 13.57
					0.8	3.3	0.7	2.7	0.6	2.5	0.7	2.6	0.6	2.4		
**b22**	H	H	Ph	H	0.25 ± 0.03		0.87 ± 0.25		2.40 ± 0.08		1.75 ± 0.15		1.24 ± 0.08		2.21 ± 0.09	14.54 ± 6.58
					8.8	58.2	2.5	16.7	0.9	6.1	1.3	8.3	1.8	11.7		
**b23 ^#^**	H	NH_2_	H	H	5.70 ± 0.09		7.23 ± 0.07		8.56 ± 0.75		7.32 ± 0.29		10.07 ± 0.03		11.41 ± 0.76	43.18 ± 11.74
					2.0	7.6	1.6	6.0	1.3	5.0	1.6	5.9	1.1	4.3		
**b24 ^#^**	OH	H	H	H	7.77 ± 0.08		13.38 ± 0.74		10.68 ± 0.38		8.64 ± 0.31		16.11 ± 0.32		8.96 ± 0.44	24.22 ± 2.71
					1.2	3.1	0.7	1.8	0.8	2.3	1.0	2.8	0.6	1.5		
**b25**	H	OH	H	H	7.27 ± 0.13		9.56 ± 0.33		8.92 ± 0.25		12.73 ± 0.28		21.03 ± 0.43		6.31 ± 0.13	19.64 ± 1.29
					0.9	2.7	0.7	2.1	0.7	2.2	0.5	1.5	0.3	0.9		
**b26 ^#^**	H	H	OH	H	14.38 ± 0.81		12.73 ± 0.81		20.24 ± 0.30		23.75 ± 1.56		20.76 ± 0.54		12.55 ± 2.82	28.16 ± 1.69
					0.9	2.0	1.0	2.2	0.6	1.4	0.5	1.2	0.6	1.4		
**b27**	OH	H	OH	H	17.39 ± 1.01		24.84 ± 0.17		23.08 ± 0.82		17.27 ± 0.76		>30		>100	35.46 ± 5.72
					>5.8	2.0	>4.0	1.4	>4.3	1.5	>5.8	2.1	-	<1.2		
**b28 ^#^**	OH	H	OCH_3_	H	19.52 ± 0.79		17.02 ± 0.65		28.37 ± 1.25		17.37 ± 0.97		28.30 ± 0.26		16.25 ± 2.48	>100
					0.8	>5.1	1.0	>5.9	0.6	>3.5	0.9	>5.8	0.6	>3.5		
**b29**	H	OCH_3_	OH	H	0.97 ± 0.04		1.83 ± 0.08		1.79 ± 0.20		1.50 ± 0.07		3.03 ± 0.05		6.31 ± 0.13	>100
					6.5	>103.1	3.4	>54.6	3.5	>55.9	4.2	>66.7	2.1	>33.0		
**b30**	H	H	CN	H	20.45 ± 0.21		>30		>30		>30		>30		12.55 ± 2.82	>100
					0.6	>4.9	<0.4	-	<0.4	-	<0.4	-	<0.4	-		
**b31**	F	H	H	H	17.98 ± 0.88		23.33 ± 0.42		23.51 ± 0.98		27.44 ± 0.67		>30		>100	>100
					>5.6	>5.6	>4.3	>4.3	>4.3	>4.3	>3.6	>3.6	-	-		
**b32**	H	F	H	H	21.56 ± 0.97		20.67 ± 0.78		25.52 ± 0.78		27.15 ± 1.39		28.38 ± 0.27		11.91 ± 1.28	72.01 ± 12.10
					0.6	3.3	0.6	3.5	0.5	2.8	0.4	2.7	0.4	2.5		
**b33**	H	H	F	H	24.55 ± 0.36		17.41 ± 0.39		32.22 ± 1.34		>30		>30		12.73 ± 2.35	63.86 ± 13.78
					0.5	2.6	0.7	3.7	0.4	2.0	<0.4	<2.1	<0.4	<2.1		
**b34**	H	Cl	H	H	13.45		16.55		21.97		16.08		21.44		9.42 ± 1.21	46.87 ± 13.91
					0.7	3.5	0.6	2.8	0.4	2.1	0.6	2.9	0.4	2.2		
**b35 ^#^**	H	H	Cl	H	27.09 ± 0.88		18.40 ± 0.06		29.76 ± 0.80		>30		>30		17.71 ± 1.35	85.43 ± 6.86
					0.7	3.2	1.0	4.6	0.6	2.9	<0.6	<2.8	<0.6	<2.8		
**b36**	H	Br	H	H	11.30 ± 1.45		13.38 ± 0.44		23.33 ± 0.79		25.03 ± 3.17		17.13 ± 0.57		13.07 ± 1.21	61.24 ± 12.11
					1.2	5.4	1.0	4.6	0.6	2.6	0.5	2.4	0.8	3.6		
**b37**	Cl	H	Cl	H	11.73 ± 0.18		15.69 ± 1.09		30.35 ± 0.71		>30		15.36 ± 0.83		19.82 ± 1.44	>100
					1.7	>8.5	1.3	>6.4	0.7	>3.3	<0.7	-	1.3	>6.5		
**b38**	H	Cl	Cl	H	18.58 ± 1.24		17.55 ± 2.01		29.58 ± 2.13		27.63 ± 0.58		26.16 ± 0.08		28.34 ± 3.74	>100
					1.5	>5.4	1.6	>5.7	1.0	>3.4	1.0	>3.6	1.1	>3.8		
**b39**	H	NO_2_	H	H	>30		>30		>30		>30		>30		>100	>100
					-	-	-	-	-	-	-	-	-	-		
**b40**	H	H	NO_2_	H	>30		>30		>30		>30		>30		>100	>100
					-	-	-	-	-	-	-	-	-	-		
**b41**	H	CF_3_	H	H	24.37 ± 5.87		29.07 ± 3.06		>30		>30		>30		>100	>100
					>4.1	>4.1	>3.4	>3.4	-	-	-	-	-	-		
**b42**	H	CF_3_	H	CF_3_	>30		>30		>30		>30		>30		>100	>100
					-	-	-	-	-	-	-	-	-	-		
**Etoposide**					5.11 ± 0.45		3.96 ± 0.17		20.57 ± 0.34		3.23 ± 0.28		29.32 ± 1.73		6.42 ± 0.39	>100
					1.3	>19.6	1.6	>25.3	0.3	>4.9	2.0	>31	0.2	>3.4		

# Known compound; *^a^* IC_50_ values were calculated by SigmaPlot 10.0 as the compounds concentrations producing half maximal inhibition after incubating the cells with increasing concentrations up to 30 μM. Data are the mean ± SD of at least three independent experiments; *^b^* CC_50_ values were calculated by SigmaPlot 10.0 as the compounds concentrations producing half maximal inhibition after incubating the cells with increasing concentrations up to 100 μM. Data are the mean ± SD of at least three independent experiments. SI = selectivity index, given by *^c^* (CC_50_^HMLE^/IC_50_^HepG2^); *^d^* (CC_50_^L02^/IC_50_^HepG2^); *^e^* (CC_50_^HMLE^/IC_50_^Hela^); *^f^* (CC_50_^L02^/IC_50_^Hela^); *^g^* (CC_50_^HMLE^/IC_50_^MCF7^); *^h^* (CC_50_^L02^/IC_50_^MCF7^); *^i^* (CC_50_^HMLE^/IC_50_^A549^); *^j^* (CC_50_^L02^/IC_50_^A549^); *^k^* (CC_50_^HMLE^/IC_50_^SW1990^) or *^l^* (CC_50_^L02^/IC_50_^SW1990^); “-” means SI value can’t be calculated.

## 3. Experimental

### 3.1. Chemistry

#### 3.1.1. General Methods

All of the solvents and reagents used in the current study were purchased from commercial suppliers and used without further purification. Melting points were determined in open capillaries and are uncorrected. The reaction products were purified by crystallization or flash column chromatography using a mixture of petroleum ether and ethyl acetate as the eluent. ^1^H-NMR spectra were recorded on Varian Inova-400 MHz, Varian Inova-500 MHz and SYS-600 MHz instruments (Varian, Palo Alto, CA, USA). ^13^C-NMR spectra were recorded on a 600 MHz Bruker ARX-600 spectrometer (Bruker Bioscience, Billerica, MA, USA). The chemical shifts (δ) are reported in ppm relative to the internal reference standard tetramethylsilane (TMS) and the coupling constants (*J* values) have been reported in Hertz (Hz). MS data were obtained using time-of-flight mass spectrometer (TOF-MS) or Bruker microTOF-Q instrument (Bruker, Billerica, MA, USA). High resolution mass spectra (HRMS) were obtained on a Q-TOF Ultima ESI instrument (micrOTOF-Q II, Bruker Daltonics, Leipzig, Germany). Analysis by thin layer chromatography (TLC) was performed on silica gel plates (Merck, Billerica, MA, USA). Automated column chromatography was conducted over silica gel using a Companion Rf 200 automated chromatography system (Teledyne ISCO, Lincoln, NE, USA). The chalcones were prepared according to methods previously described in the literature 6, 13–15 [20,21,22].

**Scheme 1:**
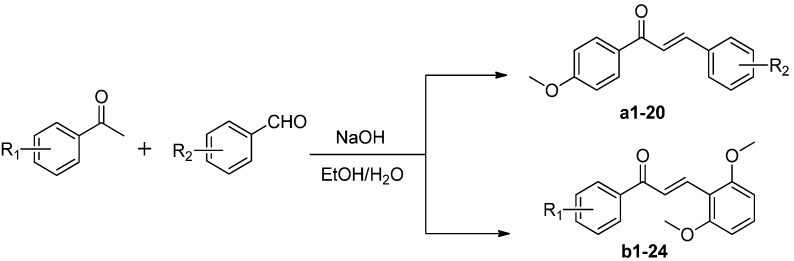
Synthesis of compounds **a1**–**20** and **b1**–**42**.

The chalcones evaluated in the current study were synthesized by the base catalyzed Claisen-Schmidt condensation reaction of the appropriately substituted acetophenones and aldehydes (Scheme 1). The chalcones prepared according to this method were formed predominantly with the (*E*)-configuration (*J*_H2-H3_ = 15–16 Hz).

#### 3.1.2. General Procedure for Synthesis of Chalcones

A 50 mL flask was charged with substituted acetophenone (5 mmol) and a solution of sodium hydroxide (10 mmol) in a 4:1 (v/v) mixture of ethanol/H_2_O (25 mL), and the resulting mixture was stirred at room temperature for 5 min. A substituted benzaldehyde (5 mmol) was then added to the reaction, and the resulting mixture was stirred at room temperature. The reaction was then monitored by TLC using ethyl acetate/petroleum ether (1:4 or 1:2 v/v) as the solvent system. Upon completion of the reaction, the crude product was filtered off and recrystallized from a mixture of dichloromethane and ethanol or purified by column chromatography over silica gel eluting with a mixture of petroleum ether and ethyl acetate to give the pure product.

#### 3.1.3. Characterization Data

*(E)-1-(4-Methoxyphenyl)-3-phenylprop-2-en-1-one* (**a1**). White solid (76%), mp: 104.1–105.3 °C ([23] 103–105 °C). ^1^H-NMR (400 MHz, DMSO-*d*_6_) δ 8.19–8.17 (m, 2H), 7.95 (d, *J* = 15.6 Hz, 1H), 7.91–7.86 (m, 2H), 7.72 (d, *J* = 15.6 Hz, 1H), 7.47–7.45 (m, 3H), 7.11–7.09 (m, 2H), 3.88 (s, 3H); MS (ESI) *m/z* = 261 (M+Na)^+^; HRMS (ESI) *m/z* calcd. for C_16_H_14_O_2_ [M+Na] 261.0896, found 261.0875 [M+Na].

*(E)-1-(4-Methoxyphenyl)-3-(4-bromophenyl)prop-2-en-1-one* (**a2**). Yellow solid (84%), mp: 153.7–155.1 °C ([24] 157 °C). ^1^H-NMR (400 MHz, DMSO-*d*_6_) δ 8.18 (d, *J* = 8.8 Hz, 2H), 7.99 (d, *J* = 15.6 Hz, 1H), 7.86 (d, *J* = 8.4 Hz, 2H), 7.68 (d, *J* = 15.6 Hz, 1H), 7.66 (d, *J* = 8.4 Hz, 2H), 7.09 (d, *J* = 8.8 Hz, 2H), 3.88 (s, 3H); MS (ESI) *m/z* = 339 (M+Na)^+^; HRMS (ESI) *m/z* calcd. for C_16_H_13_BrO_2_ [M+Na] 338.9991, found 338.9980 [M+Na].

*(E)-1-(4-Methoxyphenyl)-3-(4-bromo-2-fluorophenyl)prop-2-en-1-one* (**a3**). White solid (99%), mp: 122.2–123.6 °C. ^1^H-NMR (500 MHz, DMSO-*d*_6_) δ 8.16 (d, *J* = 9.0 Hz, 2H), 8.11 (m, 1H), 8.03 (d, *J* = 15.5 Hz, 1H), 7.73 (d, *J* = 15.5 Hz, 1H), 7.71–7.68 (m, 1H), 7.56–7.54 (m, 1H), 7.10 (d, *J* = 9.0 Hz, 2H), 3.88 (s, 3H); MS (ESI) *m/z* = 357 (M+Na)^+^.

*(E)-1-(4-Methoxyphenyl)-3-(2-bromo-4-fluorophenyl)prop-2-en-1-one* (**a4**). White solid (63%), mp: 98.7–101.0 °C. ^1^H-NMR (400 MHz, DMSO-*d*_6_) δ 8.31–8.27 (m, 1H), 8.19 (d, *J* = 9.2 Hz, 2H), 7.96 (d, *J* = 15.6 Hz, 1H), 7.92 (d, *J* = 15.6 Hz, 1H), 7.75–7.72 (m, 1H), 7.44–7.39 (m, 1H), 7.10 (d, *J* = 9.2 Hz, 2H), 3.88 (s, 3H); ^13^C-NMR (150 MHz, DMSO-*d*_6_): 187.5, 163.9, 139.8, 131.6, 131.4, 130.9, 130.6, 126.2, 125.3, 120.9, 116.2, 114.6, 56.1; MS (ESI) *m/z* = 357 (M+Na)^+^; HRMS (ESI) *m/z* calcd. for C_16_H_12_BrFO_2_ [M+Na] 356.9897, found 356.9889 [M+Na].

*(E)-1-(4-Methoxyphenyl)-3-(4-bromo-2-chlorophenyl)prop-2-en-1-one* (**a5**). White solid (86%), mp: 145.3–146.4 °C. ^1^H-NMR (500 MHz, DMSO-*d*_6_) δ 8.20–8.18 (m, 3H), 8.05 (d, *J* = 15.5 Hz, 1H), 7.92 (d, *J* = 15.5 Hz, 1H), 7.87 (s, 1H), 7.68–7.67 (m, 1H), 7.10 (m, 2H), 3.88 (s, 3H); ^13^C-NMR (150 MHz, DMSO-*d*_6_): δ 187.4, 164.0, 137.1, 135.6, 132.7, 132.3, 131.6, 131.2, 130.5, 130.4, 125.8, 124.4, 114.6, 56.1; MS (ESI) *m/z* = 351/353 (M+H)^+^.

*(E)-1-(4-Methoxyphenyl)-3-(4-nitrophenyl)prop-2-en-1-one* (**a6**).Yellow solid (63%), mp: 170.2–171.5 °C ([25] 168–169 °C). ^1^H-NMR (500 MHz, DMSO-*d*_6_) δ 8.30–8.28 (m, 2H), 8.21 (d, *J* = 9.0 Hz, 2H), 8.16–8.13 (m, 3H), 7.79 (d, *J* = 16.0 Hz, 1H), 7.11 (d, *J* = 9.0 Hz, 2H), 3.89 (s, 3H); MS (ESI) *m/z* = 284 (M+H)^+^; HRMS (ESI) *m/z* calcd. for C_16_H_13_NO_4_ [M+H] 284.0917, found 284.0916 [M+H].

*(E)-1-(4-Methoxyphenyl)-3-(4-carbonylphenyl)prop-2-en-1-one* (**a7**) [26]. Yellow solid (40%), mp: >350 °C. ^1^H-NMR (400 MHz, DMSO-*d*_6_) δ 8.18 (d, *J* = 8.8 Hz, 2H), 7.92 (d, *J* = 15.6 Hz, 1H), 7.90 (d, *J* = 8.0 Hz, 2H), 7.77 (d, *J* = 8.0 Hz, 2H), 7.71 (d, *J* = 15.6 Hz, 1H), 7.09 (d, *J* = 8.8 Hz, 2H), 3.88 (s, 3H); MS (ESI) *m/z* = 321 (M+K)^+^.

*(E)-1-(4-Methoxyphenyl)-3-(2-methoxyphenyl)prop-2-en-1-one* (**a8**). Yellow solid (82%). mp: 43.2–45.5 °C ([27] 44–45 °C). ^1^H-NMR (500 MHz, DMSO-*d*_6_) δ 8.16–8.14 (m, 2H), 8.05 (d, *J* = 15.5 Hz, 1H), 7.99–7.97 (m, 1H), 7.89 (d, *J* = 15.5 Hz, 1H), 7.47–7.44 (m, 1H), 7.13–7.09 (m, 3H), 7.06–7.03 (m, 1H), 3.91 (s, 3H), 3.88 (s, 3H); MS (ESI) *m/z* = 269 (M+H)^+^; HRMS (ESI) *m/z* calcd. for C_17_H_16_O_3_ [M+H] 269.1172, found 269.1177 [M+H].

*(E)-1-(4-Methoxyphenyl)-3-(3-methoxyphenyl)prop-2-en-1-one* (**a9**). Yellow solid (78%), mp: 95.7–97.0 °C ([28] 103–104 °C). ^1^H-NMR (500 MHz, DMSO-*d*_6_) δ 8.18 (d, *J* = 9.0 Hz, 2H), 7.95 (d, *J* = 15.5 Hz, 1H), 7.68 (d, *J* = 15.5 Hz, 1H), 7.48 (s, 1H), 7.44–7.43 (m, 1H), 7.38–7.35 (m, 1H), 7.10 (d, *J* = 9.0 Hz, 2H), 7.03–7.01 (m, 1H), 3.88 (s, 3H), 3.84 (s, 3H); MS (ESI) *m/z* = 269 (M+H)^+^; HRMS (ESI) *m/z* calcd. for C_17_H_16_O_3_ [M+H] 269.1172, found 269.1179 [M+H].

*(E)-1,3-bis(4-Methoxyphenyl)prop-2-en-1-one* (**a10**). Yellow solid (72%), mp: 97.8–100.5 °C ([28] 103–104 °C). ^1^H-NMR (400 MHz, DMSO-*d*_6_) δ 8.16 (d, *J* = 9.2 Hz, 2H), 7.85 (d, *J* = 8.8 Hz, 2H), 7.81 (d, *J* = 15.6 Hz, 1H), 7.69 (d, *J* = 15.6 Hz, 1H), 7.08 (d, *J* = 9.2 Hz, 2H), 7.02 (d, *J* = 8.8 Hz, 2H), 3.87 (s, 3H), 3.83 (s, 3H); MS (ESI) *m/z* = 291 (M+Na)^+^; HRMS (ESI) *m/z* calcd. for C_17_H_16_O_3_ [M+Na] 291.0992, found 291.0998 [M+Na].

*(E)-1-(4-Methoxyphenyl)-3-(4-ethoxyphenyl)prop-2-en-1-one* (**a11**). White solid (83%), mp: 106.3–107.9 °C ([29] 109 °C). ^1^H-NMR (500 MHz, DMSO-*d*_6_) δ 8.15 (d, *J* = 8.5 Hz, 2H), 7.84–7.78 (m, 3H), 7.68 (d, *J* = 15.5 Hz, 1H), 7.08 (d, *J* = 9.0 Hz, 2H), 7.00 (d, *J* = 8.5 Hz, 2H), 4.10 (q, *J* = 7.0 Hz, 2H), 3.87 (s, 3H), 1.35 (t, *J* = 7.0 Hz, 3H); MS (ESI) *m/z* = 283 (M+H)^+^; HRMS (ESI) *m/z* calcd. for C_18_H_18_O_3_ [M+H] 283.1329, found 283.1347 [M+H].

*(E)-1-(4-Methoxyphenyl)-3-(2,3-dimethoxyphenyl)prop-2-en-1-one* (**a12**).White solid (91%), mp: 99.8–102.0 °C ([30] 99.0–99.5 °C). ^1^H-NMR (500 MHz, DMSO-*d*_6_) δ 8.15 (d, *J* = 9.0 Hz, 2H), 7.96 (d, *J* = 15.5 Hz, 1H), 7.89 (d, *J* = 15.5 Hz, 1H), 7.63–7.61 (m, 1H), 7.16–7.15 (m, 2H), 7.09 (d, *J* = 9.0 Hz, 2H), 3.88 (s, 3H), 3.85 (s, 3H), 3.80 (s, 3H); MS (ESI) *m/z* = 299 (M+H)^+^; HRMS (ESI) *m/z* calcd. for C_18_H_18_O_4_ [M+H] 299.1278, found 299.1300 [M+H].

*(E)-1-(4-methoxyphenyl)-3-(2,5-dimethoxyphenyl)prop-2-en-1-one* (**a13**) [31]. Yellow solid (87%), mp: 54.7–56.3 °C. ^1^H-NMR (400 MHz, DMSO-*d*_6_) δ 8.16 (d, *J* = 8.8 Hz, 2H), 8.01 (d, *J* = 15.6 Hz, 1H), 7.91 (d, *J* = 15.6 Hz, 1H), 7.56–7.55 (m, 1H), 7.09 (d, *J* = 8.8 Hz, 2H), 7.07–7.00 (m, 2H), 3.87 (s, 3H), 3.85 (s, 3H), 3.80 (s, 3H); MS (ESI) *m/z* = 321 (M+Na)^+^; HRMS (ESI) *m/z* calcd. for C_18_H_18_O_4_ [M+Na] 321.1097, found 321.1098 [M+Na].

*(E)-1-(4-Methoxyphenyl)-3-(2,6-dimethoxyphenyl)prop-2-en-1-one* (**a14**). Yellow solid (70%), mp: 123.7–125.7 °C. ^1^H-NMR (400 MHz, DMSO-*d*_6_) δ 8.10 (d, *J* = 15.6 Hz, 1H), 8.04–7.99 (m, 3H), 7.42–7.38 (m, 1H), 7.10–7.08 (m, 2H), 6.77–6.75 (m, 2H), 3.92 (s, 6H), 3.87 (s, 3H); ^13^C-NMR (150 MHz, DMSO-*d*_6_): δ188.9, 163.4, 160.4, 134.5, 132.6, 131.4, 130.9, 124.1, 114.5, 112.1, 104.6, 56.5, 56.0; MS (ESI) *m/z* = 321 (M+Na)^+^; HRMS (ESI) *m/z* calcd. for C_18_H_18_O_4_ [M+Na] 321.1097, found 321.1104 [M+Na].

*(E)-1-(4-Methoxyphenyl)-3-(3,4-dimethoxyphenyl)prop-2-en-1-one* (**a15**). Yellow solid (72%), mp: 104.9–106.4 °C ([32] 93–98 °C). ^1^H-NMR (400 MHz, DMSO-*d*_6_) δ 8.17 (d, *J* = 8.8 Hz, 2H), 7.83 (d, *J* = 15.6 Hz, 1H), 7.68 (d, *J* = 15.6 Hz, 1H), 7.54–7.53 (m, 1H), 7.40–7.37 (m, 1H), 7.09 (d, *J* = 8.8 Hz, 2H), 7.03–7.01 (m, 1H), 3.87 (s, 6H), 3.82 (s, 3H); MS (ESI) *m/z* = 321 (M+Na)^+^; HRMS (ESI) *m/z* calcd. for C_18_H_18_O_4_ [M+Na] 321.1097, found 321.1083 [M+Na].

*(E)-1-(4-Methoxyphenyl)-3-(3,5-dimethoxyphenyl)prop-2-en-1-one* (**a16**) [33]. Yellow solid (85%), mp: 87.6–89.1 °C. ^1^H-NMR (500 MHz, DMSO-*d*_6_) δ 8.19 (d, *J* = 9.0 Hz, 2H), 7.94 (d, *J* = 15.5 Hz, 1H), 7.64 (d, *J* = 15.5 Hz, 1H), 7.09 (d, *J* = 9.0 Hz, 2H), 7.07–7.06 (m, 2H), 6.59–6.58 (m, 1H), 3.88 (s, 3H), 3.82 (s, 6H); ^13^C-NMR (150 MHz, DMSO-*d*_6_): 187.9, 163.7, 161.2, 143.8, 137.2, 131.5, 130.9, 123., 114.5, 107.1, 103.1, 56.0, 55.9; MS (ESI) *m/z* = 299 (M+H)^+^; HRMS (ESI) *m/z* calcd. for C_18_H_18_O_4_ [M+H]^+^ 299.1278, found 299.1298 [M+H].

*(E)-1-(4-Methoxyphenyl)-3-(3-(benzyloxy)-4-methoxyphenyl)prop-2-en-1-one* (**a17**). Yellow solid (97%), mp: 115.0–118.2 °C. ^1^H-NMR (400 MHz, DMSO-*d*_6_) δ 8.17 (d, *J* = 8.8 Hz, 2H), 7.82 (d, *J* = 15.2 Hz, 1H), 7.68–7.64 (m, 2H), 7.52–7.50 (m, 2H), 7.44–7.34 (m, 4H), 7.10 (d, *J* = 8.8 Hz, 2H), 7.06–7.04 (m, 1H), 5.19 (s, 2H), 3.87 (s, 3H), 3.83 (s, 3H); MS (ESI) *m/z* = 397 (M+Na)^+^; HRMS (ESI) *m/z* calcd. for C_22_H_24_O_4_ [M+Na]^+^ 397.1410, found 397.1407 [M+Na].

*(E)-1-(4-Methoxyphenyl)-3-(2,3,4-trimethoxyphenyl)prop-2-en-1-one* (**a18**). Yellow solid (99%), mp: 94.4–96.5 °C ([34] 94 °C). ^1^H-NMR (500 MHz, DMSO-*d*_6_) δ 8.13 (d, *J* = 9.0 Hz, 2H), 7.88 (d, *J* = 15.5 Hz, 1H), 7.81 (d, *J* = 15.5 Hz, 1H), 7.79–7.77 (m, 1H), 7.09 (d, *J* = 9.0 Hz, 2H), 6.94–6.92 (m, 1H), 3.88 (s, 3H), 3.87 (s, 3H), 3.87 (s, 3H), 3.78 (s, 3H); MS (ESI) *m/z* = 351 (M+Na)^+^; HRMS (ESI) *m/z* calcd. for C_19_H_20_O_5_ [M+Na]^+^ 351.1203, found 351.1216 [M+Na].

*(E)-1-(4-Methoxyphenyl)-3-(2,4,5-trimethoxyphenyl)prop-2-en-1-one* (**a19**). Yellow solid (94%), mp: 122.1–123.7 °C ([35] 107–110 °C). ^1^H-NMR (400 MHz, DMSO-*d*_6_) δ 8.14 (d, *J* = 8.8 Hz, 2H), 8.03 (d, *J* = 15.6 Hz, 1H), 7.76 (d, *J* = 15.6 Hz, 1H), 7.52 (s, 1H), 7.08 (d, *J* = 8.8 Hz, 2H), 6.75 (s, 1H), 3.90 (s, 3H), 3.87 (s, 3H), 3.87 (s, 3H), 3.83 (s, 3H); MS (ESI) *m/z* = 351 (M+Na)^+^; HRMS (ESI) *m/z* calcd. for C_19_H_20_O_5_ [M+Na]^+^ 351.1203, found 351.1194 [M+Na].

*(E)-1-(4-Methoxyphenyl)-3-(3,4,5-trimethoxyphenyl)prop-2-en-1-one* (**a20**). Yellow solid (84%), mp: 109.7–111.5 °C ([36] 125 °C). ^1^H-NMR (500 MHz, DMSO-*d*_6_) δ 8.18 (d, *J* = 9.0 Hz, 2H), 7.88 (d, *J* = 15.5 Hz, 1H), 7.66 (d, *J* = 15.5 Hz, 1H), 7.22 (m, 2H), 7.10 (d, *J* = 9.0 Hz, 2H), 3.88 (s, 3H), 3.87 (s, 6H), 3.72 (s, 3H); MS (ESI) *m/z* = 351 (M+Na)^+^; HRMS (ESI) *m/z* calcd. for C_19_H_20_O_5_ [M+Na]^+^ 351.1203, found 351.1183 [M+Na].

*(E)-1-Phenyl-3-(2,6-dimethoxyphenyl)prop-2-en-1-one* (**b1**). Yellow solid (84%), mp: 52.6–54.7 °C ([37] 80 °C). ^1^H-NMR (500 MHz, DMSO-*d*_6_) δ 8.13 (d, *J* = 16.0 Hz, 1H), 8.03–7.98 (m, 3H), 7.67–8.65 (m, 1H), 7.59–7.56 (m, 2H), 7.43–7.39 (m, 1H), 6.77–6.75 (m, 2H), 3.92 (s, 6H); MS (ESI) *m/z* = 291 (M+Na)^+^; HRMS (ESI) *m/z* calcd. for C_17_H_16_O_3_ [M+Na] 291.0992, found 291.1007 [M+Na].

*(E)-1-(2-Methylphenyl)-3-(2,6-dimethoxyphenyl)prop-2-en-1-one* (**b2**). Yellow solid (91%), mp: 79.8–81.4 °C. ^1^H-NMR (500 MHz, DMSO-*d*_6_) δ 7.86 (d, *J* = 16.0 Hz, 1H), 7.51 (d, *J* = 16.0 Hz, 1H), 7.49–7.47 (m, 1H), 7.44–7.38 (m, 2H), 7.34–7.31 (m, 2H), 6.74–6.73 (m, 2H), 3.85 (s, 6H), 2.36 (s, 3H); ^13^C-NMR (150 MHz, DMSO-*d*_6_): 196.8, 160.3, 140.0, 136.4, 136.3, 133.0, 131.6, 130.8, 128.7, 128.3, 126.2, 111.6, 104.7, 56.5, 20.34; MS (ESI) *m/z* = 305 (M+Na)^+^; HRMS (ESI) *m/z* calcd. for C_18_H_18_O_4_ [M+Na] 305.1148, found 305.1134 [M+Na].

*(E)-1-(3-Methylphenyl)-3-(2,6-dimethoxyphenyl)prop-2-en-1-one* (**b3**). Yellow solid (94%), mp: 102.5–103.8 °C. ^1^H-NMR (500 MHz, DMSO-*d*_6_) δ 8.12 (d, *J* = 16.0 Hz, 1H), 7.98 (d, *J* = 16.0 Hz, 1H), 7.81–7.77 (m, 2H), 7.48–7.44 (m, 2H), 7.42–7.39 (m, 1H), 6.77–6.75 (m, 2H), 3.92 (s, 6H), 2.42 (s, 3H); ^13^C-NMR (150 MHz, DMSO-*d*_6_): 190.9, 160.5, 138.7, 138.6, 135.3, 133.9, 132.8, 129.2, 128.9, 125.9, 124.3, 112.0, 104.6, 56.5, 21.41; MS (ESI) *m/z* = 305 (M+Na)^+^; HRMS (ESI) *m/z* calcd. for C_18_H_18_O_4_ [M+Na] 305.1148, found 305.1155 [M+Na].

*(E)-1-(4-Methylphenyl)-3-(2,6-dimethoxyphenyl)prop-2-en-1-one* (**b4**). Yellow solid (74%), mp: 82.7–84.5 °C. ^1^H-NMR (500 MHz, DMSO-*d*_6_) δ 8.10 (d, *J* = 16.0 Hz, 1H), 7.99 (d, *J* = 16.0 Hz, 1H), 7.91–7.89 (m, 2H), 7.42–7.37 (m, 3H), 6.77–6.75 (m, 2H), 3.92 (s, 6H), 2.40 (s, 3H); ^13^C-NMR (150 MHz, DMSO-*d*_6_): 190.2, 160.4, 143.6, 136.1, 135.0, 132.8, 129.9, 128.7, 124.1, 112.0, 104.7, 56.5, 21.64; MS (ESI) *m/z* = 305 (M+Na)^+^; HRMS (ESI) *m/z* calcd. for C_18_H_18_O_4_ [M+Na] 305.1148, found 305.1135 [M+Na].

*(E)-1-(3,4-Dimethylphenyl)-3-(2,6-dimethoxyphenyl)prop-2-en-1-one* (**b5**). Yellow solid (90%), mp: 98.9–101.1 °C. ^1^H-NMR (500 MHz, DMSO-*d*_6_) δ 8.08 (d, *J* = 16.0 Hz, 1H), 7.98 (d, *J* = 16.0 Hz, 1H), 7.75–7.72 (m, 2H), 7.41–7.38 (m, 1H), 7.33–7.32 (m, 1H), 6.77–6.75 (m, 2H), 3.92 (s, 6H), 2.32 (s, 3H), 2.31 (s, 3H); ^13^C-NMR (150 MHz, DMSO-*d*_6_): 190.3, 160.4, 142.5, 137.4, 136.5, 134.9, 132.7, 130.3, 129.5, 126.4, 124.3, 112.0, 104.7, 56.6, 20.1, 19.9; MS (ESI) *m/z* = 319 (M+Na)^+^; HRMS (ESI) *m/z* calcd. for C_19_H_20_O_3_ [M+Na] 319.1305, found 319.1302 [M+Na].

*(E)-1-(2-Methoxyphenyl)-3-(2,6-dimethoxyphenyl)prop-2-en-1-one* (**b6**) [38]. Yellow solid (84%), mp: 109.0–111.4 °C. ^1^H-NMR (500 MHz, DMSO-*d*_6_) δ 7.91 (d, *J* = 16.5 Hz, 1H), 7.71 (d, *J* = 16.5 Hz, 1H), 7.56–7.46 (m, 2H), 7.39–7.18 (m, 2H), 7.07–7.04 (m, 1H), 6.74–6.72 (m, 2H), 3.88 (s, 3H), 3.86 (s, 6H); MS (ESI) *m/z* = 321 (M+Na)^+^; HRMS (ESI) *m/z* calcd. for C_18_H_18_O_4_ [M+Na] 321.1097, found 321.1080 [M+Na].

*(E)-1-(3-Methoxyphenyl)-3-(2,6-dimethoxyphenyl)prop-2-en-1-one* (**b7**). Yellow solid (93%), mp: 93.5–95.6 °C. ^1^H-NMR (400 MHz, DMSO-*d*_6_) δ 8.12 (d, *J* = 16.0 Hz, 1H), 7.97 (d, *J* = 16.0 Hz, 1H), 7.58–7.51 (m, 1H), 7.49–7.45 (m, 1H), 7.44–7.41 (m, 2H), 7.25–7.22 (m, 1H), 6.77–6.75 (m, 2H), 3.92 (s, 6H), 3.85 (s, 3H); ^13^C-NMR (150 MHz, DMSO-*d*_6_): 190.5, 160.5, 160.0, 140.1, 135.5, 132.9, 130.5, 124.2, 121.1, 119.3, 113.0, 111.9, 104.7, 56.6, 55.7; MS (ESI) *m/z* = 321 (M+Na)^+^. HRMS (ESI) *m/z* calcd. for C_18_H_18_O_4_ [M+Na] 321.1097, found 321.1098 [M+Na].

*(E)-1-(2-Ethoxyphenyl)-3-(2,6-dimethoxyphenyl)prop-2-en-1-one* (**b8**). Yellow solid (94%). mp: 48.8–50.4 °C. ^1^H-NMR (400 MHz, DMSO-*d*_6_) δ 7.93 (d, *J* = 16.0 Hz, 1H), 7.74 (d, *J* = 16.0 Hz, 1H), 7.53–7.44 (m, 2H), 7.39–7.35 (m, 1H), 7.17–7.15 (m, 1H), 7.05–7.02 (m, 1H), 6.74–6.72 (m, 2H), 4.16 (q, *J* = 7.2 Hz, 2H), 3.86 (s, 6H), 1.31 (t, *J* = 7.2 Hz, 3H); ^13^C-NMR (150 MHz, DMSO-*d*_6_): 193.8, 160.3, 157.4, 133.5, 133., 132.5, 130.1, 130.0, 129.5, 121.0, 113.7, 111.9, 104.7, 64.4, 56.4, 14.9; MS (ESI) *m/z* = 335 (M+Na)^+^; HRMS (ESI) *m/z* calcd. for C_19_H_20_O_4_ [M+Na] 335.1254, found 335.1248 [M+Na].

*(E)-1-(3-Ethoxyphenyl)-3-(2,6-dimethoxyphenyl)prop-2-en-1-one* (**b9**). Yellow solid (42%), mp: 63.1–64.8 °C. ^1^H-NMR (400 MHz, DMSO-*d*_6_) δ 8.11 (d, *J* = 16.0 Hz, 1H), 7.95 (d, *J* = 16.0 Hz, 1H), 7.57–7.55 (m, 1H), 7.50–7.46 (m, 1H), 7.44–7.38 (m, 2H), 7.24–7.18 (m, 1H), 6.77–6.75 (m, 2H), 4.12 (q, *J* = 7.2 Hz, 2H), 3.92 (s, 6H), 1.36 (t, *J* = 7.2 Hz, 3H); ^13^C-NMR (150 MHz, DMSO-*d*_6_): 190.5, 160.5, 159.2, 140.1, 135.5, 132.8, 130.4, 124.3, 120.9, 119.7, 113.5, 112.0, 104.6, 63.7, 56.5, 15.0; MS (ESI) *m/z* = 335 (M+Na)^+^; HRMS (ESI) *m/z* calcd. for C_19_H_20_O_4_ [M+Na] 335.1254, found 335.1255 [M+Na].

*(E)-1-(4-Ethoxyphenyl)-3-(2,6-dimethoxyphenyl)prop-2-en-1-one* (**b10**). White solid (82%), mp: 99.0–101.2 °C. ^1^H-NMR (500 MHz, DMSO-*d*_6_) δ 8.09 (d, *J* = 15.5 Hz, 1H), 8.03–7.98 (m, 3H), 7.41–7.38 (m, 1H), 7.09–7.07 (m, 2H), 6.77–6.75 (m, 2H), 4.14 (q, *J* = 7.0 Hz, 2H), 3.92 (s, 6H), 1.37 (t, *J* = 7.0 Hz, 3H); ^13^C-NMR (150 MHz, DMSO-*d*_6_): 188.9, 162.7, 160.4, 134.5, 132.6, 131.2, 130.9, 124.0, 114.9, 112.1, 104.6, 64.0, 56.5, 15.0; MS (ESI) *m/z* = 335 (M+Na)^+^; HRMS (ESI) *m/z* calcd. for C_19_H_20_O_4_ [M+Na] 335.1254, found 335.1256 [M+Na].

*(E)-1-(2-(Benzyloxy>)phenyl)-3-(2,6-dimethoxyphenyl)prop-2-en-1-one* (**b11**). Yellow solid (20%), mp: 94.6–97.9 °C. ^1^H-NMR (400 MHz, DMSO-*d*_6_) δ 7.92 (d, *J* = 16.0 Hz, 1H), 7.76 (d, *J* = 16.0 Hz, 1H), 7.53–7.47 (m, 2H), 7.45–7.35 (m, 3H), 7.25–7.21(m, 4H), 7.09–7.05 (m, 1H), 6.73–6.71 (m, 2H), 5.24 (s, 2H), 3.78 (s, 6H); ^13^C-NMR(150 MHz, DMSO-*d*_6_): 193.1, 157.6, 157.4, 137.4, 133.4, 131.7, 130.6, 129.9, 129.8, 128.9, 128.8, 128.1, 127.7, 120.7, 114.0, 113.7, 104.0, 70.11, 55.7; MS (ESI) *m/z* = 397 (M+Na)^+^; HRMS (ESI) *m/z* calcd. for C_24_H_22_O_4_ [M+Na] 397.1410, found 397.1395 [M+Na].

*(E)-1-(3-(Benzyloxy)phenyl)-3-(2,6-dimethoxyphenyl)prop-2-en-1-one* (**b12**). Yellow solid (81%), mp: 108.8–110.2 °C. ^1^H-NMR (400 MHz, DMSO-*d*_6_) δ 8.11 (d, *J* = 16.0 Hz, 1H), 7.95 (d, *J* = 16.0 Hz, 1H), 7.60–7.58 (m, 1H), 7.54–7.47 (m, 4H), 7.43–7.38 (m, 3H), 7.37–7.33 (m, 1H), 7.32–7.29 (m, 1H), 6.77–7.75 (m, 2H), 5.21 (s, 2H), 3.91 (s, 6H); ^13^C-NMR (150 MHz, DMSO-*d*_6_): 190.5, 160.5, 159.1, 140.1, 137.3, 135.5, 132.9, 130.5, 128.9, 128.4, 128.2, 124.2, 121.3, 120.2, 114.0, 111.9, 104.7, 69.8, 56.6; MS (ESI) *m/z* = 397 (M+Na)^+^; HRMS (ESI) *m/z* calcd. for C_24_H_22_O_4_ [M+Na] 397.1410, found 397.1394 [M+Na].

*(E)-1-(4-(Benzyloxy)phenyl)-3-(2,6-dimethoxyphenyl)prop-2-en-1-one* (**b13**) [39]. White solid (97%), mp: 96.6–97.8 °C. ^1^H-NMR (500 MHz, DMSO-*d*_6_) δ 8.10 (d, *J* = 16.0 Hz, 1H), 8.04–7.98 (m, 3H), 7.49–7.47 (m, 2H), 7.44–7.33 (m, 4H), 7.18–7.16 (m, 2H), 6.77–6.75 (m, 2H), 5.23 (s, 2H), 3.92 (s, 6H); MS (ESI) *m/z* = 375 (M+H)^+^; HRMS (ESI) *m/z* calcd. for C_24_H_22_O_4_ [M+H] 375.1591, found 375.1591 [M+H].

*(E)-1-(2-(Benzyloxy)-4-methoxyphenyl)-3-(2,6-dimethoxyphenyl)prop-2-en-1-one* (**b14**). Yellow solid (75%), mp: 82.2–83.6 °C. ^1^H-NMR (400 MHz, DMSO-*d*_6_) δ 7.94 (d, *J* = 16.0, 1H), 7.90 (d, *J* = 16.0, 1H), 7.59–7.57 (m, 1H), 7.45–7.43 (m, 2H), 7.38–7.34 (m, 1H), 7.24–7.22 (m, 3H), 6.76–6.75 (m, 1H), 6.72–6.70 (m, 2H), 6.67–6.64 (m, 1H), 5.27 (s, 2H), 3.83 (s, 3H), 3.76 (s, 6H); MS (ESI) *m/z* = 427 (M+Na)^+^; HRMS (ESI) *m/z* calcd. for C_25_H_24_O_5_ [M+Na] 427.1516, found 427.1512 [M+Na].

*(E)-1-(4-(Benzyloxy)-3-methoxyphenyl)-3-(2,6-dimethoxyphenyl)prop-2-en-1-one* (**b15**). Yellow solid (80%), mp: 112.8–114.1 °C. ^1^H-NMR (400 MHz, DMSO-*d*_6_) δ 8.09 (d, *J* = 16.0 Hz, 1H), 8.02 (d, *J* = 16.0 Hz, 1H), 7.67–7.65 (m, 1H), 7.55–7.54 (m, 1H), 7.51–7.45 (m, 2H), 7.45–7.32 (m, 4H), 7.21–7.19 (m, 1H), 6.77–6.75 (m, 2H), 5.21 (s, 2H), 3.92 (s, 6H), 3.87 (s, 3H); ^13^C-NMR (150 MHz, DMSO-*d*_6_): 188.9, 160.4, 152.4, 149.5, 137.0, 134.5, 132.6, 131.7, 129.0, 128.5, 128.4, 124.1, 123.0, 112.9, 112.1, 111.2, 104.7, 70.4, 56.5, 56.0; MS (ESI) *m/z* = 405 (M+H)^+^; HRMS (ESI) *m/z* calcd. for C_25_H_24_O_5_ [M+H] 405.1697, found 405.1693 [M+H].

*(E)-1-(4-Ethoxy-3-methoxyphenyl)-3-(2,6-dimethoxyphenyl)prop-2-en-1-one* (**b16**). Yellow solid (91%), mp: 105.3–107.2 °C. ^1^H-NMR (400 MHz, DMSO-*d*_6_) δ 8.08 (d, *J* = 16.0 Hz, 1H), 8.02 (d, *J* = 16.0 Hz, 1H), 7.68–7.65 (m, 1H), 7.52–7.51 (m, 1H), 7.41–7.37 (m, 1H), 7.11–7.09 (m, 1H), 6.77–6.75 (m, 2H), 4.14 (q, *J* = 7.2 Hz, 2H), 3.92 (s, 6H), 3.86 (s, 3H), 1.38 (t, *J* = 7.2 Hz, 3H); ^13^C-NMR (150 MHz, DMSO-*d*_6_): 188.9, 160.4, 152.7, 149.3, 134.3, 132.5, 131.3, 124.1, 123.1, 112.1, 112.0, 111.0, 104.7, 64.4, 56.5, 55.8, 15.0; MS (ESI) *m/z* = 365 (M+Na)^+^; HRMS (ESI) *m/z* calcd. for C_20_H_22_O_5_ [M+Na] 365.1359, found 365.1350 [M+Na].

*(E)-1-(2-Ethoxy-4-methoxyphenyl)-3-(2,6-dimethoxyphenyl)prop-2-en-1-one* (**b17**). Yellow solid (65%), mp: 98.7–100.2 °C. ^1^H-NMR (400 MHz, DMSO-*d*_6_) δ 7.95 (d, *J* = 16.0 Hz, 1H), 7.89 (d, *J* = 16.0 Hz, 1H), 7.57–7.55 (m, 1H), 7.38–7.33 (m, 1H), 6.74–6.72 (m, 2H), 6.68–6.59 (m, 2H), 4.18 (q, *J* = 7.2 Hz, 2H), 3.87 (s, 6H), 3.84 (s, 3H), 1.34 (t, *J* = 7.2 Hz, 3H); ^13^C-NMR (150 MHz, DMSO-*d*_6_): 191.3, 164.1, 160.3, 159.7, 132.4, 132.3, 132.1, 129.8, 122.7, 112.3, 106.5, 104.6, 99.8, 64.5, 56.4, 56.0, 14.8; MS (ESI) *m/z* = 365 (M+Na)^+^; HRMS (ESI) *m/z* calcd. for C_20_H_22_O_5_ [M+Na] 365.1359, found 365.1357 [M+Na].

*(E)-1-(2,5-Dimethoxyphenyl)-3-(2,6-dimethoxyphenyl)prop-2-en-1-one* (**b18**). White solid (92%), mp: 121.0–122.0 °C. ^1^H-NMR (400 MHz, DMSO-*d*_6_) δ 7.91 (d, *J* = 16.0 Hz, 1H), 7.72 (d, *J* = 16.0 Hz, 1H), 7.40–7.36 (m, 1H), 7.16–7.08 (m, 2H), 7.02–7.01 (m, 1H), 6.73–6.71 (m, 2H), 3.87 (s, 6H), 3.82 (s, 3H), 3.75 (s, 3H); ^13^C-NMR (150 MHz, DMSO-*d*_6_): 193.1, 160.3, 153.6, 152.4, 134.1, 132.6, 130.3, 129.3, 118.8, 114.5, 114.4, 112.0, 104.7, 56.8, 56.5, 56.0; MS (ESI) *m/z* = 351 (M+Na)^+^; HRMS (ESI) *m/z* calcd. for C_19_H_20_O_5_Na [M+Na] 351.1203, found 351.1189 [M+Na].

*(E)-1-(3,4-Dimethoxyphenyl)-3-(2,6-dimethoxyphenyl)prop-2-en-1-one* (**b19**) [15]. Yellow solid (80%), mp: 120.5–122.7 °C. ^1^H-NMR (500 MHz, DMSO-*d*_6_) δ 8.08 (d, *J* = 16.0 Hz, 1H), 8.03 (d, *J* = 16.0 Hz, 1H), 7.69–7.67 (m, 1H), 7.52–7.51 (m, 1H), 7.40–7.38 (m, 1H), 7.13–7.11 (m, 1H), 6.77–7.75 (m, 2H), 3.92 (s, 6H), 3.87 (s, 3H), 3.85 (s, 3H); MS (ESI) *m/z* = 329 (M+H)^+^.

*(E)-1-(Benzo[d][1,3]dioxol-5-yl)-3-(2,6-dimethoxyphenyl)prop-2-en-1-one* (**b20**) [40]. White solid (95%), mp: 138.6–140.4 °C. ^1^H-NMR (500 MHz, DMSO-*d*_6_) δ 8.08 (d, *J* = 16.0 Hz, 1H), 7.96 (d, *J* = 16.0 Hz, 1H), 7.66–7.64 (m, 1H), 7.47–7.46 (m, 1H), 7.40–7.38 (m, 1H), 7.08–7.06 (m, 1H), 6.76–7.75 (m, 2H), 6.16 (s, 2H), 3.92 (s, 6H); MS (ESI) *m/z* = 313 (M+H)^+^; HRMS (ESI) *m/z* calcd. for C_18_H_16_O_5_ [M+H] 313.1071, found 313.1068 [M+H].

*(E)-1-(2,3-Dihydrobenzofuran-5-yl)-3-(2,6-dimethoxyphenyl)prop-2-en-1-one* (**b21**). Yellow solid (99%), mp: 118.8–120.2 °C. ^1^H-NMR (400 MHz, DMSO-*d*_6_) δ 8.07 (d, *J* = 15.6 Hz, 1H), 7.98 (d, *J* = 15.6 Hz, 1H), 7.91–7.90 (m, 1H), 7.87–7.84 (m, 1H), 7.41–7.37 (m, 1H), 6.91–6.89 (m, 1H), 6.77–6.75 (m, 2H), 4.65 (t, *J* = 8.8 Hz, 2H), 3.92 (s, 6H), 3.27 (t, *J* = 8.8 Hz, 2H); ^13^C-NMR (150 MHz, DMSO-*d*_6_): 188.7, 164.3, 160.3, 134.3, 132.5, 131.7, 130.4, 128.9, 126.0, 124.2, 112.1, 109.4, 104.7, 72.6, 56.5, 28.9; MS (ESI) *m/z* = 333 (M+Na)^+^; HRMS (ESI) *m/z* calcd. for C_19_H_18_O_4_ [M+Na] 333.1097, found 333.1098 [M+Na].

*(E)-1-([1,1'-Biphenyl]-4-yl)-3-(2,6-dimethoxyphenyl)prop-2-en-1-one* (**b22**). Yellow solid (70%), mp: 121.7–123.5 °C. ^1^H-NMR (500 MHz, DMSO-*d*_6_) δ 8.17 (d, *J* = 16.0 Hz, 1H), 8.10–8.08 (m, 2H), 8.06 (d, *J* = 16.0 Hz, 1H), 7.88–7.86 (m, 2H), 7.78–7.76 (m, 2H), 7.54–7.51 (m, 2H), 7.46–7.40 (m, 2H), 6.78–6.76 (m, 2H), 3.94 (s, 6H); ^13^C-NMR (150 MHz, DMSO-*d*_6_): 190.1, 160.5, 144.7, 139.5, 137.4, 135.4, 132.9, 129.6, 129.3, 128.8, 127.5, 127.4, 124.1, 112.0, 104.7, 56.6; MS (ESI) *m/z* = 345 (M+H)^+^; HRMS (ESI) *m/z* calcd. for C_23_H_20_O_3_ [M+H] 345.1485, found 345.1478 [M+H].

*(E)-1-(3-Aminophenyl)-3-(2,6-dimethoxyphenyl)prop-2-en-1-one* (**b23**) [41]. Yellow solid (67%), mp: 148.6–150.3 °C. ^1^H-NMR (600 MHz, DMSO-*d*_6_) δ 8.06 (d, *J* = 16.2 Hz, 1H), 7.92 (d, *J* = 16.2 Hz, 1H), 7.41–7.38 (m, 1H), 7.21–7.17 (m, 2H), 7.13–7.12 (m, 1H), 6.84–6.80 (m, 1H), 6.77–6.75 (m, 2H), 5.39 (s, 2H), 3.92 (s, 6H); MS (ESI) *m/z* = 306 (M+Na)^+^; HRMS (ESI) *m/z* calcd. for C_17_H_17_NO_3_ [M+Na] 306.1101, found 306.1097 [M+Na].

*(E)-1-(2-Hydroxyphenyl)-3-(2,6-dimethoxyphenyl)prop-2-en-1-one* (**b24**) [42]. Yellow solid (83%), mp: 114.2–115.9 °C. ^1^H-NMR (400 MHz, DMSO-*d*_6_) δ 12.55 (s, 1H), 8.25 (d, *J* = 15.6 Hz, 1H), 8.13 (d, *J* = 15.6 Hz, 1H), 7.96–7.94 (m, 1H), 7.59–7.52 (m, 1H), 7.45–7.41 (m, 1H), 7.03–6.99 (m, 2H), 6.78–6.76 (m, 2H), 3.94 (s, 6H); MS (ESI) *m/z* = 285 (M+H)^+^; HRMS (ESI) *m/z* calcd. for C_17_H_16_O_4_ [M+H] 285.1121, found 285.1133 [M+H].

*(E)-1-(3-Hydroxyphenyl)-3-(2,6-dimethoxyphenyl)prop-2-en-1-one* (**b25**). Yellow solid (70%), mp: 138.1–139.7 °C. ^1^H-NMR (400 MHz, DMSO-*d*_6_) δ 9.79 (s, 1H), 8.09 (d, *J* = 16.0 Hz, 1H), 7.94 (d, *J* = 16.0 Hz, 1H), 7.46–7.33 (m, 4H), 7.05–7.02 (m, 1H), 6.77–6.75 (m, 2H), 3.92 (s, 6H); ^13^C-NMR (150 MHz, DMSO-*d*_6_): 190.5, 160.5, 158.2, 140.0, 135.2, 132.8, 130.4, 124.2, 120.4, 119.5, 114.8, 112.0, 104.7, 56.6; MS (ESI) *m/z* = 307 (M+Na)^+^; HRMS (ESI) *m/z* calcd. for C_17_H_16_O_4_ [M+Na] 307.0941, found 307.0939 [M+Na].

*(E)-1-(4-Hydroxyphenyl)-3-(2,6-dimethoxyphenyl)prop-2-en-1-one* (**b26**) [43]. Yellow solid (82%), mp: 189.6–191.4 °C. ^1^H-NMR (500 MHz, DMSO-*d*_6_) δ 10.35 (s, 1H), 8.06 (d, *J* = 16.0 Hz, 1H), 7.99 (d, *J* = 16.0 Hz, 1H), 7.92–7.90 (m, 2H), 7.40–7.36 (m, 1H), 6.91–6.89 (m, 2H), 6.76–6.74 (m, 2H), 3.92 (s, 6H); MS (ESI) *m/z* = 307 (M+Na)^+^; HRMS (ESI) *m/z* calcd. for C_17_H_16_O_4_ [M+Na] 307.0941, found 307.0938 [M+Na].

*(E)-1-(2,4-Dihydroxyphenyl)-3-(2,6-dimethoxyphenyl)prop-2-en-1-one* (**b27**). Yellow solid (85%), mp: 218.7–220.2 °C. ^1^H-NMR (600 MHz, DMSO-*d*_6_) δ 13.51 (s, 1H), 10.70 (s, 1H), 8.21 (d, *J* = 15.6 Hz, 1H), 8.06 (d, *J* = 15.6 Hz, 1H), 7.88–7.86 (m, 1H), 7.43–7.40 (m, 1H), 6.77–6.76 (m, 2H), 6.46–6.44 (m, 1H), 6.30–6.29 (m, 1H), 3.93 (s, 6H); ^13^C-NMR (150 MHz, DMSO-*d*_6_): 192.8, 166.2, 165.4, 160.5, 135.1, 133.1, 132.7, 122.7, 113.6, 111.9, 108.9, 104.7, 103.2, 56.6; MS (ESI) *m/z* = 323 (M+Na)^+^; HRMS (ESI) *m/z* calcd. for C_17_H_16_O_5_ [M+Na] 323.0890, found 323.0876 [M+Na].

*(E)-1-(2-Hydroxy-4-methoxyphenyl)-3-(2,6-dimethoxyphenyl)prop-2-en-1-one* (**b28**) Yellow solid (74%), mp: 143.5–144.7 °Cs ([13] 140 °C). ^1^H-NMR (500 MHz, DMSO-*d*_6_) δ 13.55 (s, 1H), 8.21 (d, *J* = 15.5 Hz, 1H), 8.10 (d, *J* = 15.5 Hz, 1H), 7.93–7.91 (m, 1H), 7.42–7.40 (m, 1H), 6.77–6.75 (m, 2H), 6.57–6.47 (m, 2H), 3.93 (s, 6H), 3.83 (s, 3H); MS (ESI) *m/z* = 315 (M+H)^+^; HRMS (ESI) *m/z* calcd. for C_18_H_18_O_5_ [M+H] 315.1227, found 315.1243 [M+H].

*(E)-1-(4-Hydroxy-3-methoxyphenyl)-3-(2,6-dimethoxyphenyl)prop-2-en-1-one* (**b29**). Yellow solid (40%), mp: 128.7–131.2 °C. ^1^H-NMR (500 MHz, DMSO-*d*_6_) δ 10.02 (s, 1H), 8.06 (d, *J* = 16.0 Hz, 1H), 8.01 (d, *J* = 16.0 Hz, 1H), 7.59–7.57 (m, 1H), 7.53–7.52 (m, 1H), 7.40–7.36 (m, 1H), 6.94–6.92 (m, 1H), 6–6.74 (m, 2H), 3.92 (s, 6H), 3.86 (s, 3H); ^13^C-NMR (150 MHz, DMSO-*d*_6_): 188.6, 160.3, 152.1, 148.3, 134.0, 132.4, 130.4, 124.2, 123.5, 115.5, 112.2, 111.7, 104.6, 56.5, 56.0; MS (ESI) *m/z* = 337 (M+Na)^+^; HRMS (ESI) *m/z* calcd. for C_18_H_18_O_5_ [M+Na] 337.1046, found 337.1042 [M+Na].

*(E)-1-(4-Cyanophenyl)-3-(2,6-dimethoxyphenyl)prop-2-en-1-one* (**b30**). Yellow solid (95%), mp: 144.3–145.9 °C. ^1^H-NMR (500 MHz, DMSO-*d*_6_) δ 8.16 (d, *J* = 16.0 Hz, 1H), 8.13–8.11 (m, 2H), 8.04–8.02 (m, 2H), 7.96 (d, *J* = 16.0 Hz, 1H), 7.45–7.42 (m, 1H), 6.78–6.76 (m, 2H), 3.92 (s, 6H); ^13^C-NMR (150 MHz, DMSO-*d*_6_): 190.1, 160.7, 142.0, 136.8, 133.4, 133.3, 129.2, 123.6, 118.7, 115.2, 111.7, 104.7, 56.6; MS (ESI) *m/z* = 294 (M+H)^+^; HRMS (ESI) *m/z* calcd. for C_18_H_15_NO_3_ [M+H] 294.1125, found 294.1130 [M+H].

*(E)-1-(2-Fluorophenyl)-3-(2,6-dimethoxyphenyl)prop-2-en-1-one* (**b31**). Yellow solid (93%), mp: 66.6–68.1 °C. ^1^H-NMR (500 MHz, DMSO-*d*_6_) δ 8.06–8.02 (m, 1H), 7.76–7.70 (m, 2H), 7.66–7.64 (m, 1H), 7.44–7.34 (m, 3H), 6.76–6.74 (m, 2H), 3.88 (s, 6H); 190.2, 160.52, 136.0, 134.5, 133.2, 130.9, 127.9, 125.3, 117.1, 111.6, 104.7, 56.5; MS (ESI) *m/z* = 287 (M+H)^+^; HRMS (ESI) *m/z* calcd. for C_17_H_15_FO_3_ [M+H] 287.1078, found 287.1097 [M+H].

*(E)-1-(3-Fluorophenyl)-3-(2,6-dimethoxyphenyl)prop-2-en-1-one* (**b32**). Yellow solid (93%), mp: 105.4–107.3 °C. ^1^H-NMR (500 MHz, DMSO-*d*_6_) δ 8.15 (d, *J* = 16.0 Hz, 1H), 7.96 (d, *J* = 16.0 Hz, 1H), 7.86–7.84 (m, 1H), 7.72–7.70 (m, 1H), 7.64–7.62 (m, 1H), 7.53–7.51 (m, 1H), 7.43–7.41 (m, 1H), 6.78–7.76 (m, 2H), 3.93 (s, 6H); ^13^C-NMR (150 MHz, DMSO-*d*_6_): 189.6, 160.6, 140.9, 136.2, 133.2, 131.5, 124.8, 123.7, 120.2, 115.0, 111.8, 104.6, 56.6; MS (ESI) *m/z* = 287 (M+H)^+^; HRMS (ESI) *m/z* calcd. for C_17_H_15_FO_3_ [M+H] 287.1078, found 287.1086 [M+H].

*(E)-1-(4-Fluorophenyl)-3-(2,6-dimethoxyphenyl)prop-2-en-1-one* (**b33**). Yellow solid (89%), mp: 108.2–109.6 °C. ^1^H-NMR (500 MHz, DMSO-*d*_6_) δ 8.13 (d, *J* = 16.0 Hz, 1H), 8.10–8.06 (m, 2H), 7.99 (d, *J* = 16.0 Hz, 1H), 7.44–7.36 (m, 3H), 6.77–6.75 (m, 2H), 3.92 (s, 6H); ^13^C-NMR (150 MHz, DMSO-*d*_6_): 189.2, 160.5, 135.6, 135.2, 133.0, 131.6, 123.7, 116.4, 111.9, 104.7, 56.5. MS (ESI) *m/z* = 309 (M+Na)^+^; HRMS (ESI) *m/z* calcd. for C_17_H_15_FO_3_ [M+Na] 309.0897, found 309.0890 [M+Na].

*(E)-1-(3-Chlorophenyl)-3-(2,6-dimethoxyphenyl)prop-2-en-1-one* (**b34**). Yellow solid (96%), mp: 116.5–117.2 °C. ^1^H-NMR (500 MHz, DMSO-*d*_6_) δ 8.14 (d, *J* = 16.0 Hz, 1H), 7.96–7.92 (m, 3H), 7.75–7.71 (m, 1H), 7.63–7.60 (m, 1H), 7.44–7.41 (m, 1H), 6.77–6.75 (m, 2H), 3.92 (s, 6H); ^13^C-NMR (150 MHz, DMSO-*d*_6_): 189.6, 160.6, 140.5, 136.3, 134.3, 133.2, 132.9, 131.3, 128.1, 127.3, 123.6, 111.8, 104.7, 56.6; MS (ESI) *m/z* = 303 (M+H)^+^; HRMS (ESI) *m/z* calcd. for C_17_H_15_ClO_3_ [M+H] 303.0782, found 303.0782 [M+H].

*(E)-1-(4-Chlorophenyl)-3-(2,6-dimethoxyphenyl)prop-2-en-1-one* (**b35**) [44]. Yellow solid (92%), mp: 107.9–109.7 °C. ^1^H-NMR (500 MHz, DMSO-*d*_6_) δ 8.14 (d, *J* = 16.0 Hz, 1H), 8.02–7.99 (m, 2H), 7.97 (d, *J* = 16.0 Hz, 1H), 7.65–7.61 (m, 2H), 7.44–7.40 (m, 1H), 6.77–6.75 (m, 2H), 3.92 (s, 6H); MS (ESI) *m/z* = 325 (M+Na)^+^; HRMS (ESI) *m/z* calcd. for C_17_H_15_ClO_3_ [M+Na] 325.0602, found 325.0597 [M+Na].

*(E)-1-(3-Bromophenyl)-3-(2,6-dimethoxyphenyl)prop-2-en-1-one* (**b36**). Yellow solid (98%), mp: 135.7–138.2 °C. ^1^H-NMR (500 MHz, DMSO-*d*_6_) δ 8.14 (d, *J* = 16.0 Hz, 1H), 8.06–8.05 (m, 1H), 8.01–7.97 (m, 1H), 7.93 (d, *J* = 16.0 Hz, 1H), 7.87–7.85 (m, 1H), 7.56–7.53 (m, 1H), 7.44–7.40 (m, 1H), 6.77–6.75 (m, 2H), 3.92 (s, 6H); ^13^C-NMR (150 MHz, DMSO-*d*_6_): 189.5, 160.6, 140.7, 136.3, 135.8, 133.2, 131.6, 131.0, 127.7, 123.6, 122.8, 111.8, 104.7, 56.6; MS (ESI) *m/z* = 347 (M+H)^+^; HRMS (ESI) *m/z* calcd. for C_17_H_15_BrO_3_ [M+H] 347.0277, found 347.0257 [M+H].

*(E)-1-(2,4-Dichlorophenyl)-3-(2,6-dimethoxyphenyl)prop-2-en-1-one* (**b37**). Yellow solid (99%), mp: 111.1–112.7 °C. ^1^H-NMR (500 MHz, DMSO-*d*_6_) δ 7.82 (d, *J* = 16.5 Hz, 1H), 7.78 (s, 1H), 7.59–7.54 (m, 2H), 7.45 (d, *J* = 16.5 Hz, 1H), 7.42–7.40 (m, 1H), 6.75–6.73 (m, 2H), 3.85 (s, 6H); ^13^C-NMR (150 MHz, DMSO-*d*_6_): 193.6, 160.5, 138.5, 137.7, 135.9, 133.7, 131.6, 131.1, 130.1, 128.1, 128.0, 111.3, 104.7, 56.6; MS (ESI) *m/z* = 359 (M+Na)^+^; HRMS (ESI) *m/z* calcd. for C_17_H_14_Cl_2_O_3_ [M+Na] 359.0212, found 359.0215 [M+Na].

*(E)-1-(3,4-Dichlorophenyl)-3-(2,6-dimethoxyphenyl)prop-2-en-1-one* (**b38**). Yellow solid (98%), mp: 142.1–143.4 °C. ^1^H-NMR (500 MHz, DMSO-*d*_6_) δ 8.15 (d, *J* = 16.0 Hz, 1H), 8.12–8.10 (m, 1H), 7.97–7.95 (m, 1H), 7.93 (d, *J* = 16.0 Hz, 1H), 7.84–7.82 (m, 1H), 7.44–7.41 (m, 1H), 6.77–6.75 (m, 2H), 3.92 (s, 6H); ^13^C-NMR (150 MHz, DMSO-*d*_6_): 188.7, 160.6, 138.8, 136.6, 136.0, 133.3, 132.4, 131.7, 130.3, 128.7, 123.3, 111.8, 104.7, 56.6; MS (ESI) *m/z* = 359 (M+Na)^+^; HRMS (ESI) *m/z* calcd. for C_17_H_14_Cl_2_O_3_ [M+Na] 359.0212, found 359.0189 [M+Na].

*(E)-1-(3-Nitrophenyl)-3-(2,6-dimethoxyphenyl)prop-2-en-1-one* (**b39**). Yellow solid (92%), mp: 172.3–173.7 °C. ^1^H-NMR (500 MHz, DMSO-*d*_6_) δ 8.66–8.63 (m, 1H), 8.49–8.47 (m, 1H), 8.44–8.42 (m, 1H), 8.20 (d, *J* = 16.0 Hz, 1H), 8.02 (d, *J* = 16.0 Hz, 1H), 7.89–7.86 (m, 1H), 7.46–7.42 (m, 1H), 6.77–6.75 (m, 2H), 3.93 (s, 6H); ^13^C-NMR (150 MHz, DMSO-*d*_6_): 189.0, 160.7, 148.6, 139.7, 136.9, 134.7, 133.5, 131.2, 127.5, 123.2, 122.9, 111.7, 104.7, 56.6; MS (ESI) *m/z* = 336 (M+Na)^+^; HRMS (ESI) *m/z* calcd. for C_17_H_15_NO_5_ [M+Na] 336.0842, found 336.0825 [M+Na].

*(E)-1-(4-Nitrophenyl)-3-(2,6-dimethoxyphenyl)prop-2-en-1-one* (**b40**). Yellow solid (97%), mp: 147.3–148.6 °C. ^1^H-NMR (400 MHz, DMSO-*d*_6_) δ 8.39–8.36 (m, 2H), 8.21–8.16 (m, 3H), 7.98 (d, *J* = 15.6 Hz, 1H), 7.46–7.42 (m, 1H), 6.78–6.76 (m, 2H), 3.93 (s, 6H); ^13^C-NMR (150 MHz, DMSO-*d*_6_): 189.9, 160.7, 150.1, 143.5, 136.9, 133.5, 130.0, 124.4, 123.7, 111.7, 104.7, 56.6; MS (ESI) *m/z* = 314 (M+H)^+^; HRMS (ESI) *m/z* calcd. for C_17_H_15_NO_5_ [M+H] 314.1023, found 314.1022 [M+H].

*(E)-1-(3-(Trifluoromethyl)phenyl)-3-(2,6-dimethoxyphenyl)prop-2-en-1-one* (**b41**). Yellow solid (97%), mp: 145.3–146.7 °C. ^1^H-NMR (500 MHz, DMSO-*d*_6_) δ 8.31–8.29 (m, 1H), 8.19 (s, 1H), 8.17 (d, *J* = 16.0 Hz, 1H), 8.04–8.02 (m, 1H), 7.99 (d, *J* = 16.0 Hz, 1H), 7.85–7.82 (m, 1H), 7.45–7.41 (m, 1H), 6.77–6.75 (m, 2H), 3.93 (s, 6H); ^13^C-NMR (150 MHz, DMSO-*d*_6_): 189.7, 160.6, 139.3, 136.6, 133.3, 132.6, 130.8, 130.2, 129.9, 129.6, 124.8, 123.6, 111.8, 104.7, 56.6; MS (ESI) *m/z* = 337 (M+H)^+^; HRMS (ESI) *m/z* calcd. for C_18_H_15_F_3_O_3_ [M+H] 337.1046, found 337.1039 [M+H].

*(E)-1-(3,5-bis(Trifluoromethyl)phenyl)-3-(2,6-dimethoxyphenyl)prop-2-en-1-one* (**b42**). Yellow solid (99%), mp: 126.5–127.8 °C. ^1^H-NMR (500 MHz, DMSO-*d*_6_) δ 8.47 (s, 2H), 8.43 (s, 1H), 8.16 (d, *J* = 16.0 Hz, 1H), 7.95 (d, *J* = 16.0 Hz, 1H), 7.46–7.43 (m, 1H), 6.77–6.75 (m, 2H), 3.91 (s, 6H); ^13^C-NMR (150 MHz, DMSO-*d*_6_): 188.9, 160.6, 140.6, 137.5, 133.6, 131.4, 131.2, 129.0, 126.4, 124.4, 123.6, 122.6, 111.7, 104.7, 56.6; MS (ESI) *m/z* = 405 (M+H)^+^; HRMS (ESI) *m/z* calcd. for C_19_H_14_F_6_O_3_ [M+H] 405.0920, found 405.0917 [M+H].

### 3.2. Biology

#### 3.2.1. Cell Culture

HepG2 and MCF7 cells were grown in the Eagle’s minimum essential medium with Earle’s balanced salts (MEM-EBSS) medium, HeLa, A549 and L02 cells were grown in Dulbecco’s Modified Eagle Media (DMEM), SW1990 cells were grown in RPMI-1640 medium, and HMLE cell were grown in DMEM/F-12 medium. The media were supplemented with 100 U/mL penicillin, 100 mg/mL streptomycin and 10% fetal bovine serum (FBS), and incubated at 37 °C in a humidified atmosphere containing 5% CO_2_.

#### 3.2.2. *In Vitro* Anti-Proliferative Assay

Cells were seeded into 96-well plates (4000 cells/well) and incubated at 37 °C in a humidified 5% CO_2_ atmosphere. After 24 h, the cells were treated with different concentrations of the compounds for 48 h in triplicate to generate dose-response curves. Cell viability was evaluated using the sulforhodamine B (SRB) assay as previous described [45]. The IC_50_ values of the compounds were calculated using SigmaPlot10.0 software, which defined the IC_50_ value as the concentration required to inhibit cell growth by 50%. Etoposide was used as the positive control.

## 4. Conclusions

In summary, we have developed a class of alkoxylated chalcones and evaluated their cytotoxicities *in vitro* against a panel of five different cancer cell lines and two non-tumoral cell lines. Most of the chalcones displayed potent cytotoxic activities. The results of this study also revealed that chalcones bearing electron donating alkoxy groups on their A- or B-ring generally exhibited good levels of cytotoxicity, and that the position and size of the substituents had a significant impact on the cytotoxic activity, with *ortho* and *meta* substituents being beneficial to the activity and large substituents having the opposite effect. The most potent compounds were **b22** and **b29**, which had a 4-phenyl moiety and a 3-OMe-4-OH moiety, respectively. The SI values indicate that about one third of the compounds exhibit more than a 2-fold SI between the CC_50_ in L02 and IC_50_ in tumoral cells, which is similar with etoposide. Compounds **b11**, especially **b29** showed promising SI values compared with both HMLE and L02 (2.1–6.5 fold in HMLE and over 33 fold in L02, respectively). These compounds are therefore worthy of further investigation.

## Supplementary Materials

Supplementary materials can be accessed at: http://www.mdpi.com/1420-3049/19/11/17256/s1.

## Supplementary Files

Supplementary File 1
