# Supplementary material for: Synthesis and Cytotoxic Evaluation of Alkoxylated Chalcones
Source: Molecules. 2014 Oct 28;19(11):17256–78. doi: 10.3390/molecules191117256 (PMC6271338; doi:10.3390/molecules191117256)

Supplementary Materials

^{1}H-NMR spectra, ^{13}C-NMR spectra or HRMS of the synthesized compounds.

(E)-1-(4-Methoxyphenyl)-3-phenylprop-2-en-1-one (**a1**)

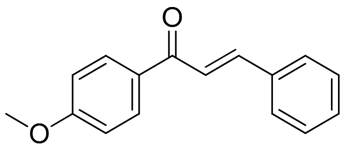

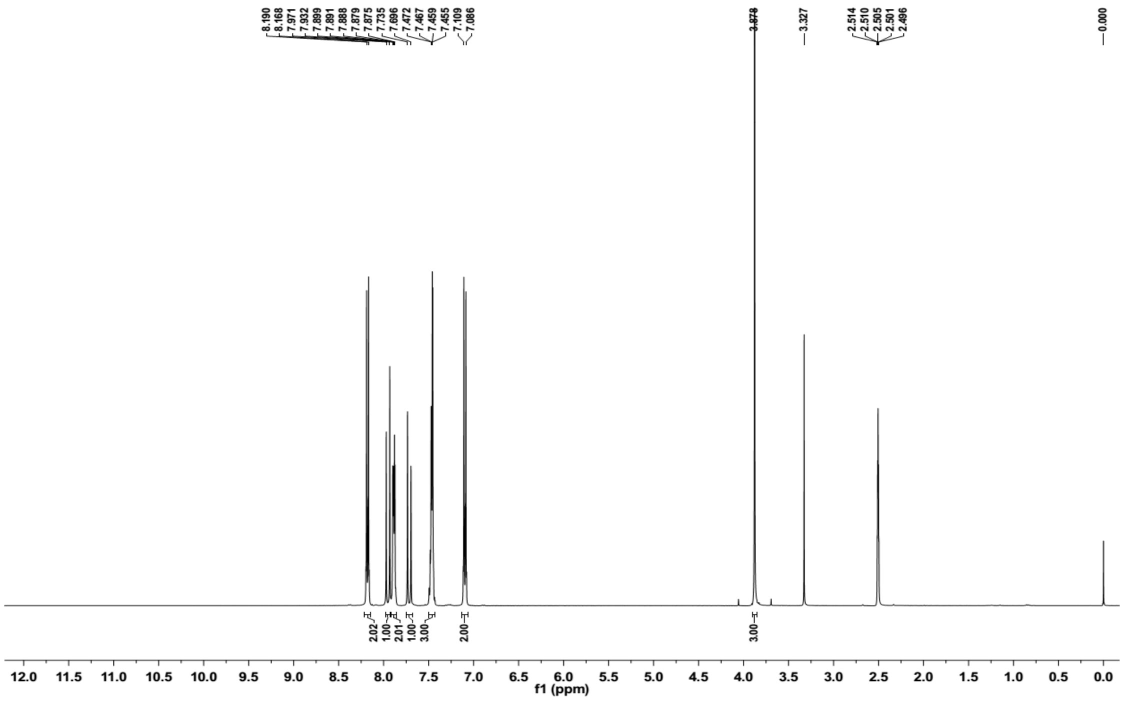

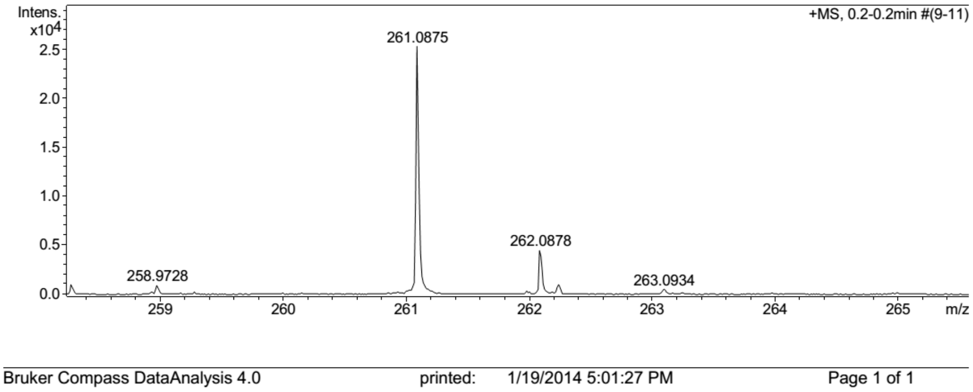

The HRMS ionization method is below, also for other compounds.

Display Report

Analysis Info

Analysis Name	D:\Data\du\wyc\a\4b.d	Acquisition Date	1/10/2014 8:50:42 PM
Method	tune_low.m	Operator	XZNU
Sample Name	TuneMix	Instrument	micrOTOF-Q 134
Comment			

Acquisition Parameter

Source Type	ESI	Ion Polarity	Positive	Set Nebulizer	0.4 Bar
Focus	Active	Set Capillary	4500 V	Set Dry Heater	180 °C
Scan Begin	50 m/z	Set End Plate Offset	-500 V	Set Dry Gas	2.0 l/min
Scan End	3000 m/z	Set Collision Cell RF	150.0 Vpp	Set Divert Valve	Source

(E)-1-(4-Methoxyphenyl)-3-(4-bromophenyl)prop-2-en-1-one (**a2**)

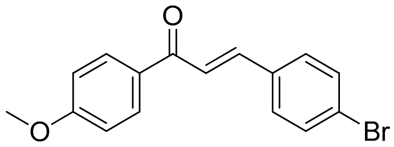

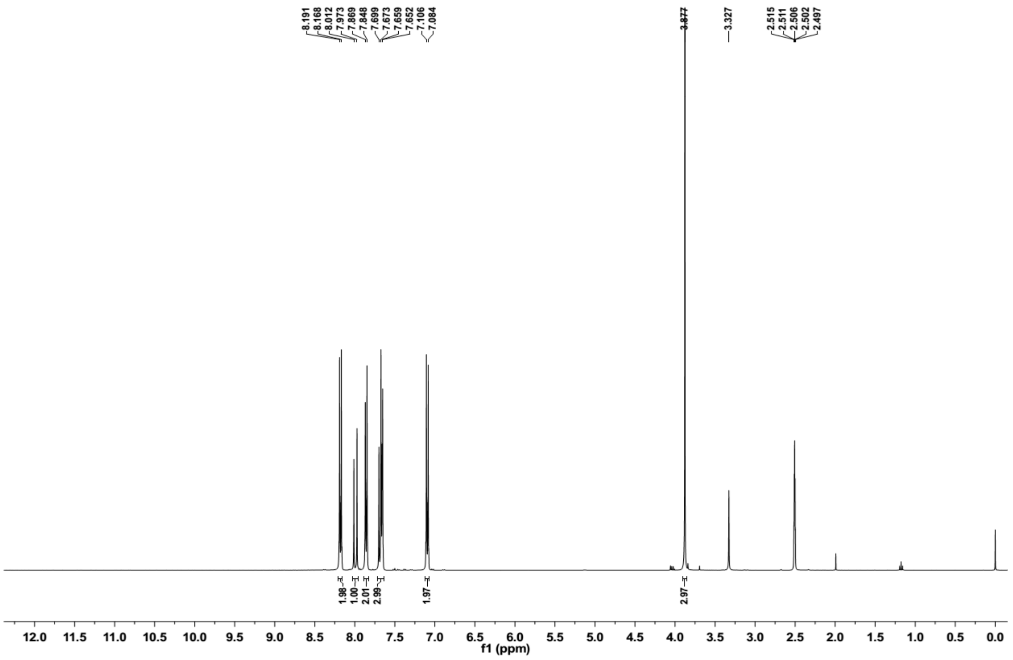

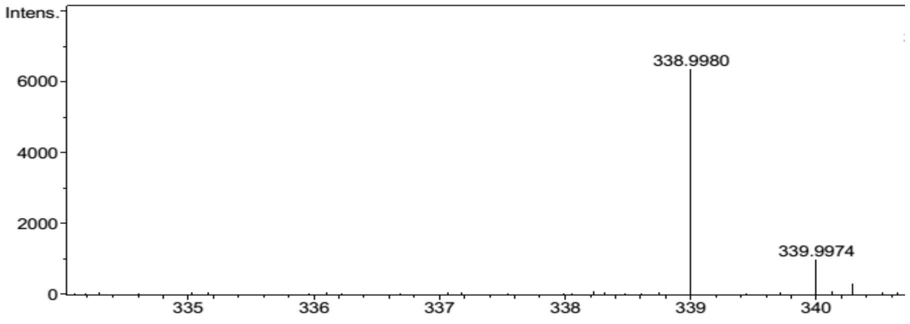

Bruker Compass DataAnalysis 4.0 printed: 1/19/2014 5:18:17 PM

(E)-1-(4-Methoxyphenyl)-3-(4-bromo-2-fluorophenyl)prop-2-en-1-one (**a3**)

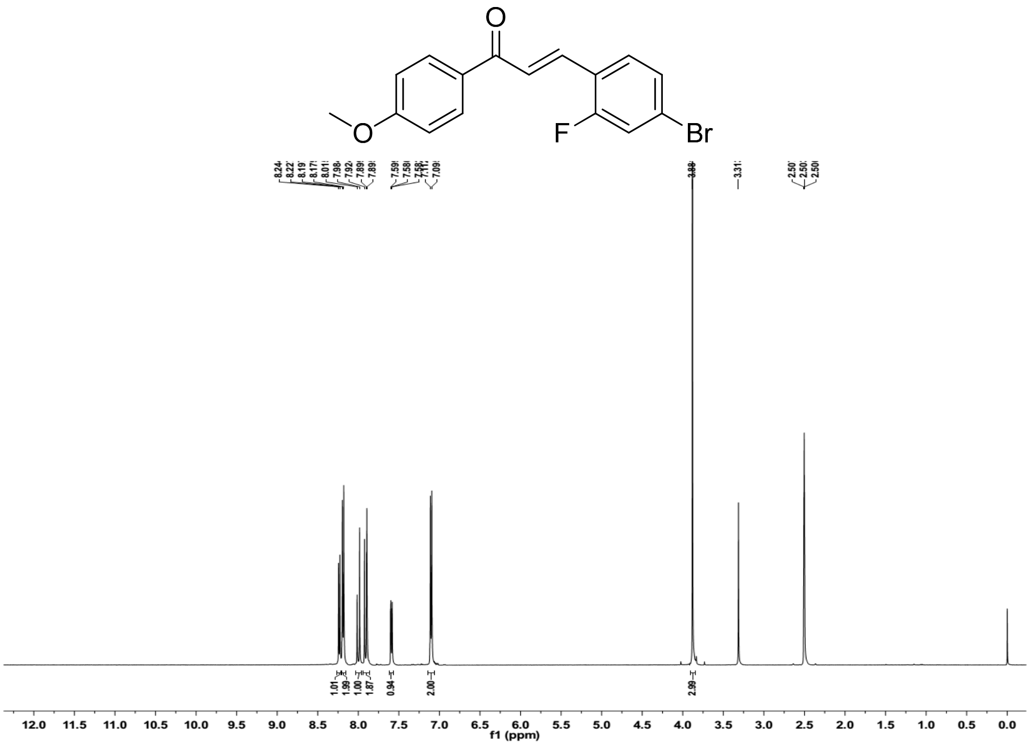

(E)-1-(4-Methoxyphenyl)-3-(2-bromo-4-fluorophenyl)prop-2-en-1-one (**a4**)

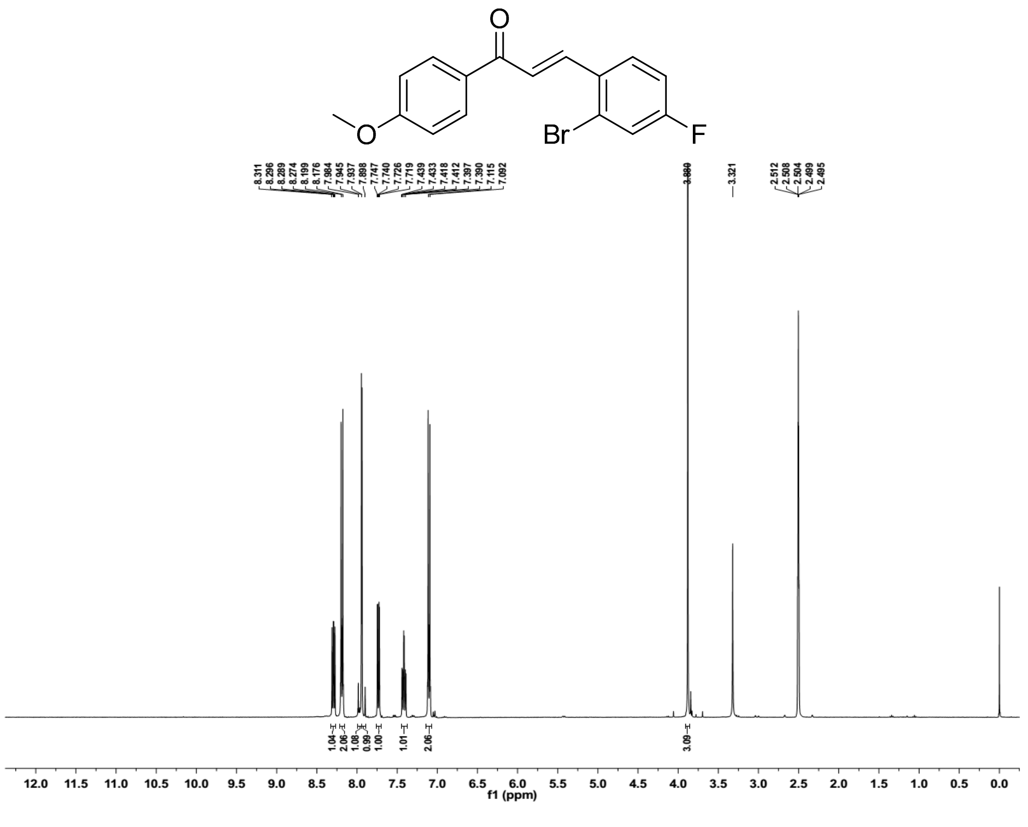

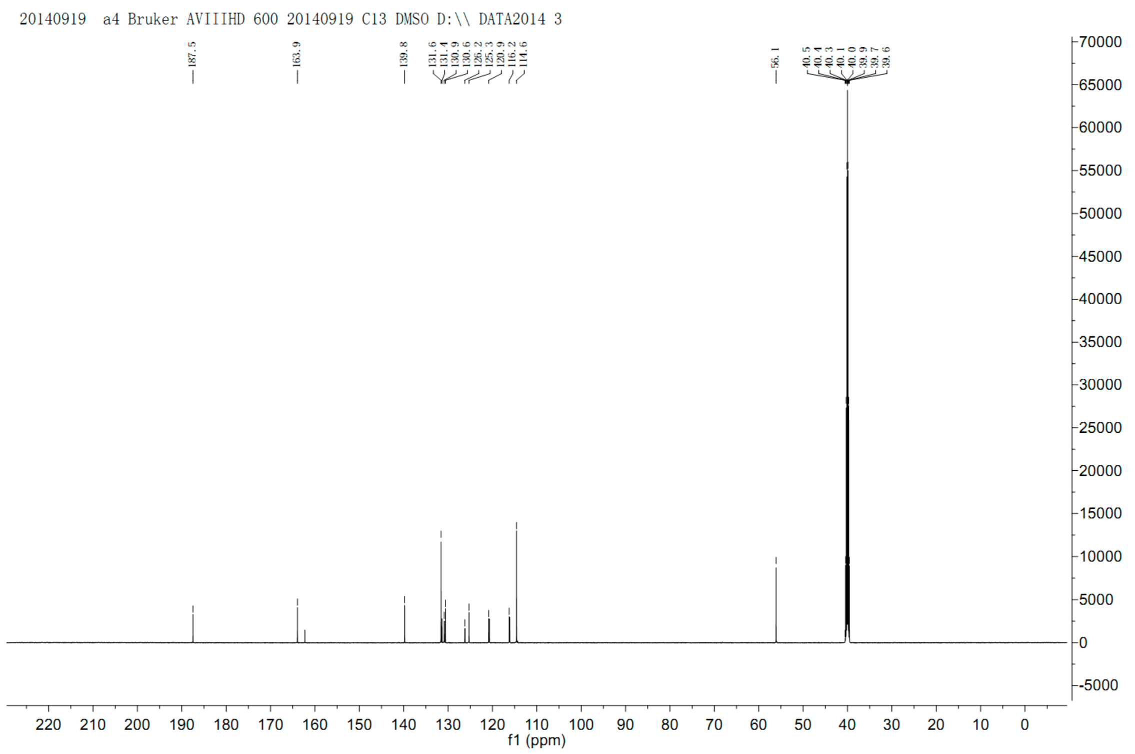

(E)-1-(4-Methoxyphenyl)-3-(4-bromo-2-chlorophenyl)prop-2-en-1-one (**a5**)

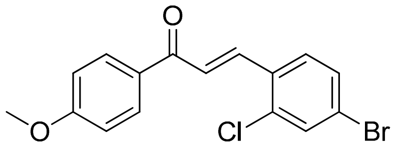

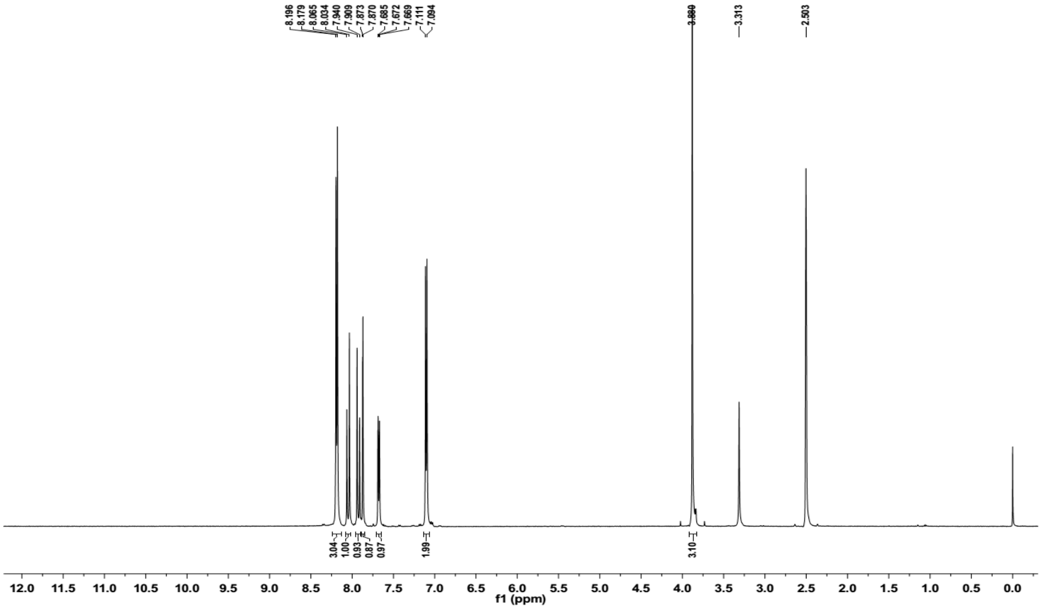

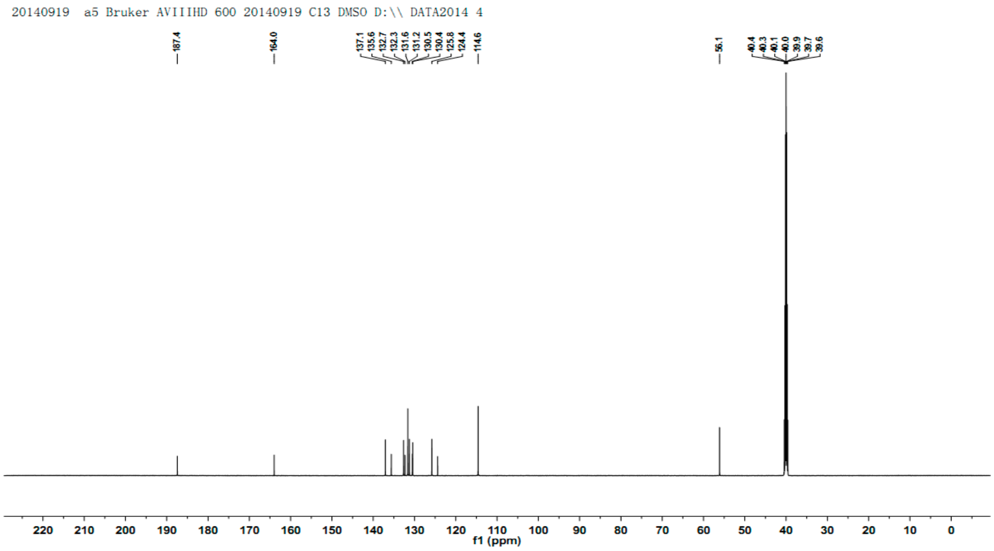

(E)-1-(4-Methoxyphenyl)-3-(4-nitrophenyl)prop-2-en-1-one (**a6**)

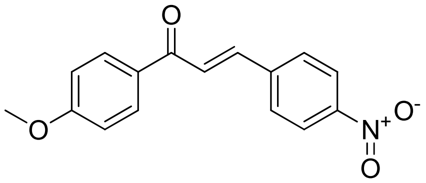

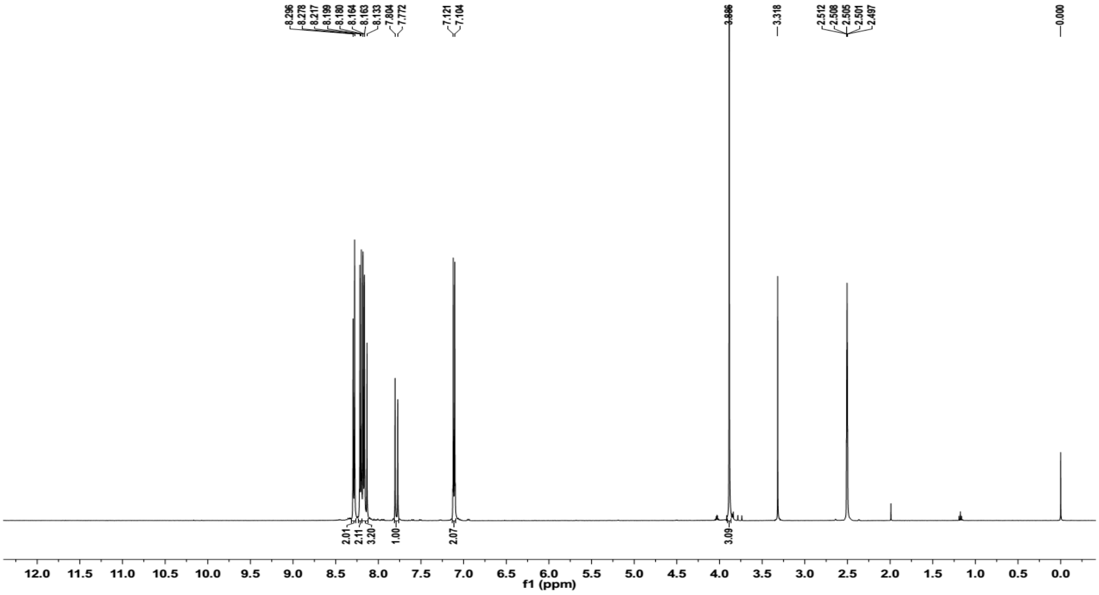

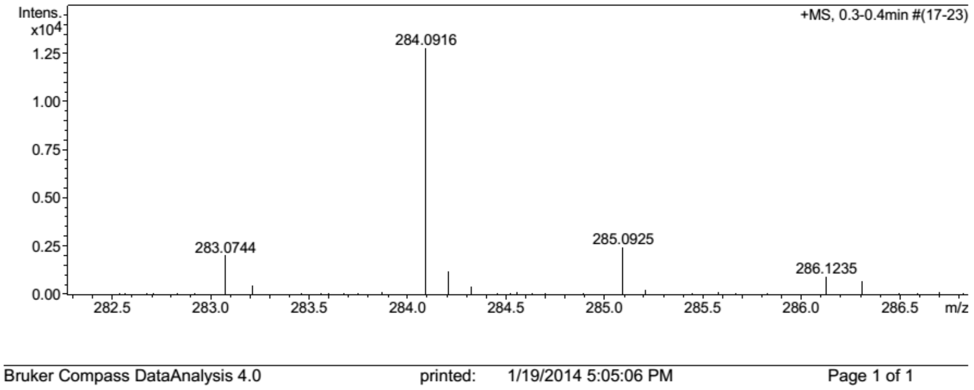

(E)-1-(4-Methoxyphenyl)-3-(4-carbonylphenyl)prop-2-en-1-one (**a7**)

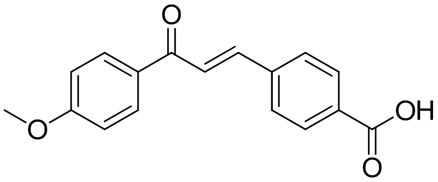

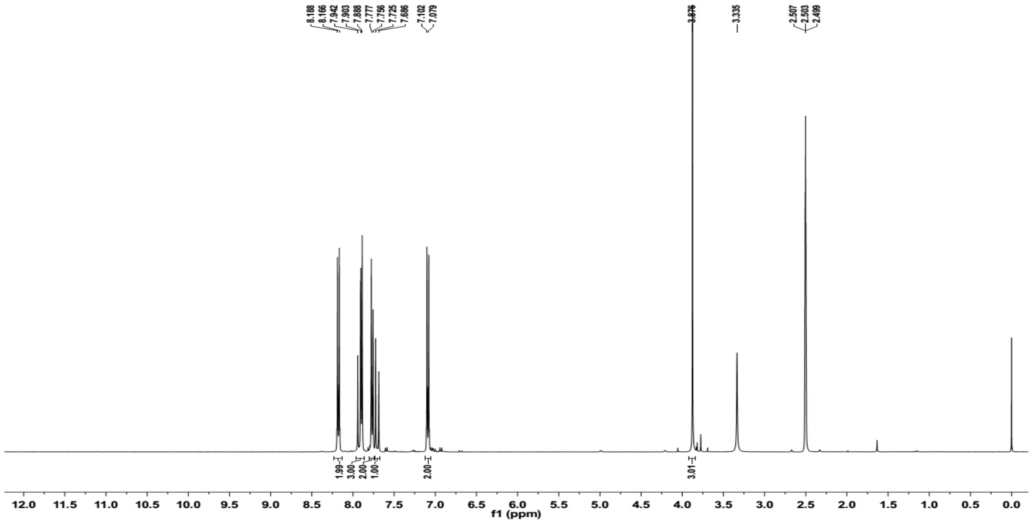

(E)-1-(4-Methoxyphenyl)-3-(2-methoxyphenyl)prop-2-en-1-one (**a8**)

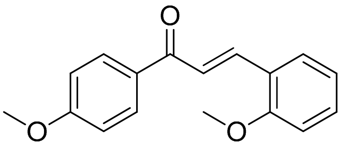

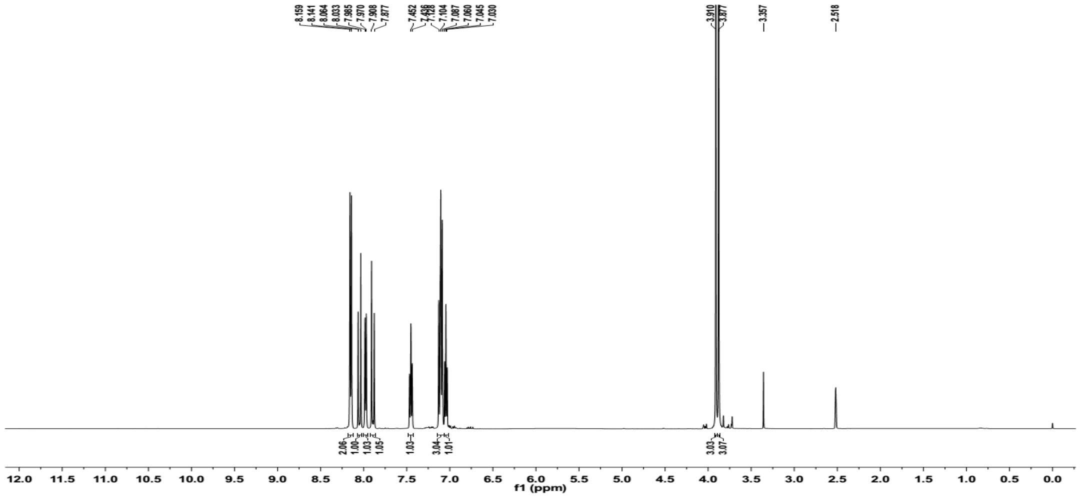

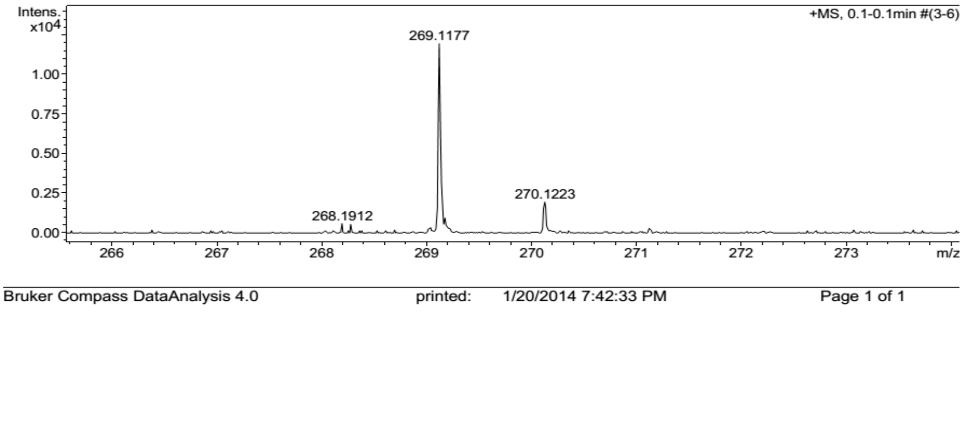

(E)-1-(4-Methoxyphenyl)-3-(3-methoxyphenyl)prop-2-en-1-one (**a9**)

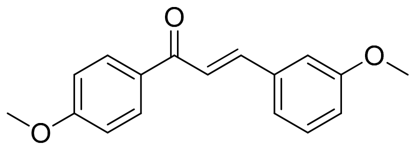

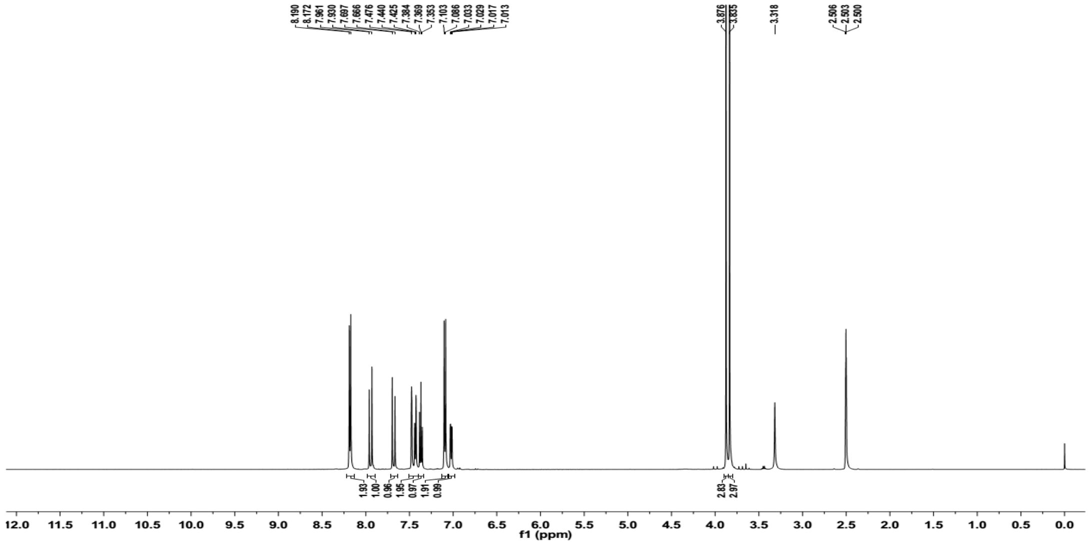

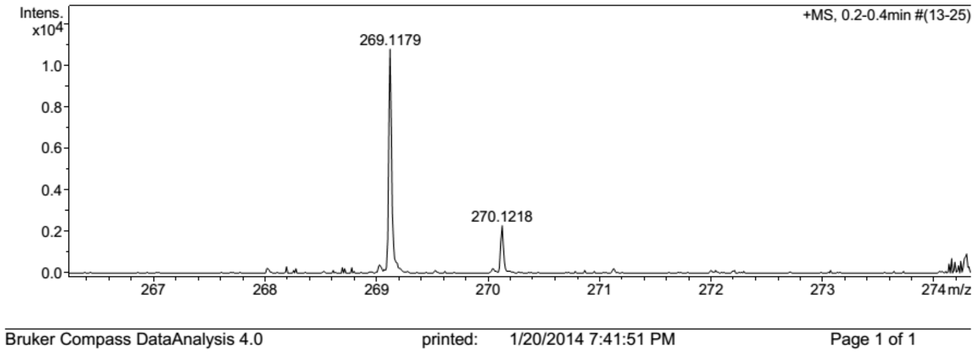

(E)-1,3-bis(4-Methoxyphenyl)prop-2-en-1-one (**a10**)

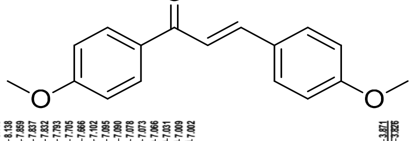

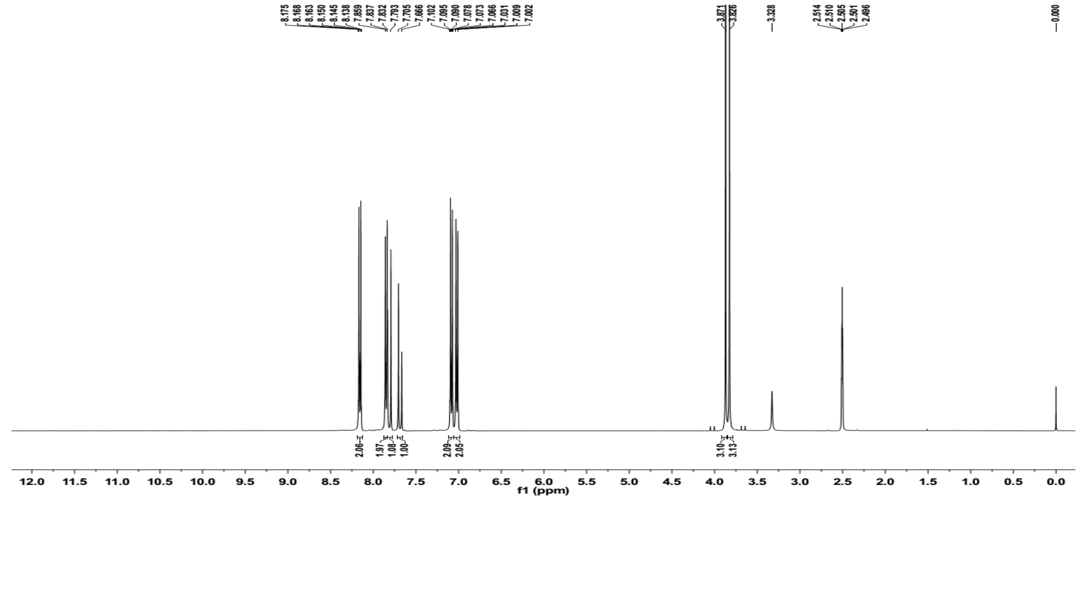

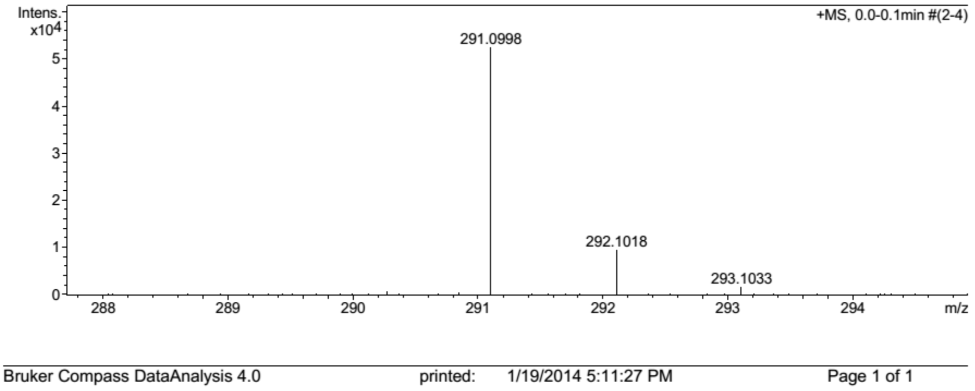

(E)-1-(4-Methoxyphenyl)-3-(4-ethoxyphenyl)prop-2-en-1-one (**a11**)

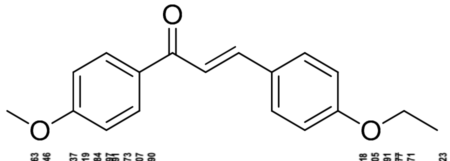

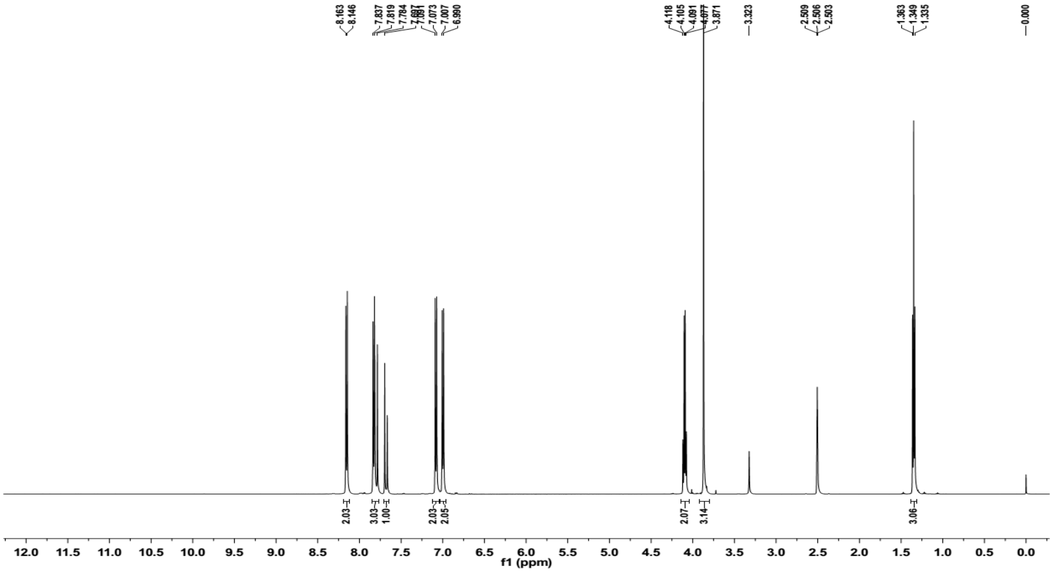

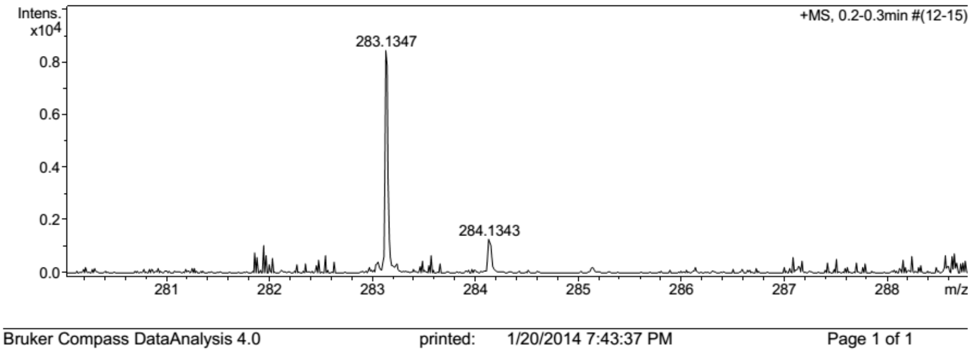

(E)-1-(4-Methoxyphenyl)-3-(2,3-dimethoxyphenyl)prop-2-en-1-one (**a12**)

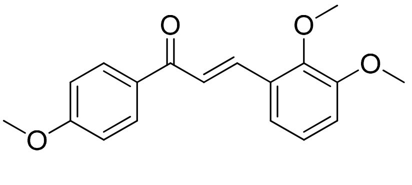

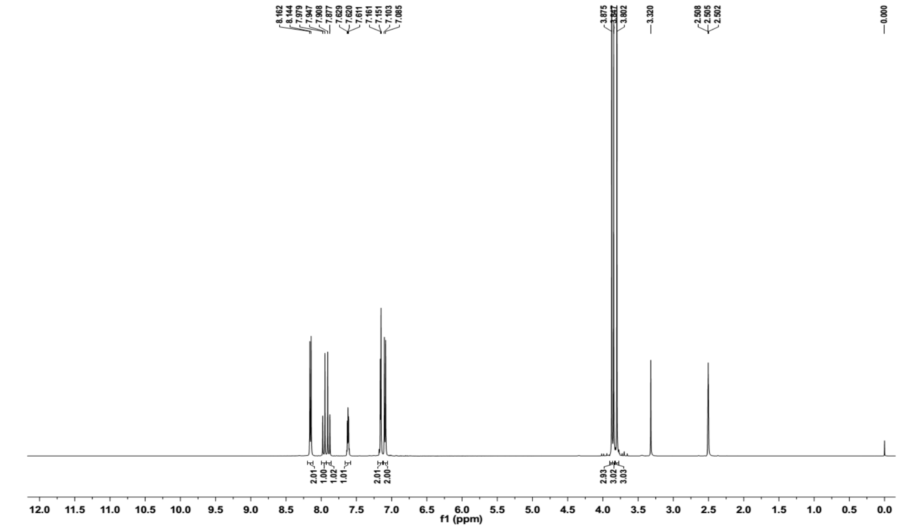

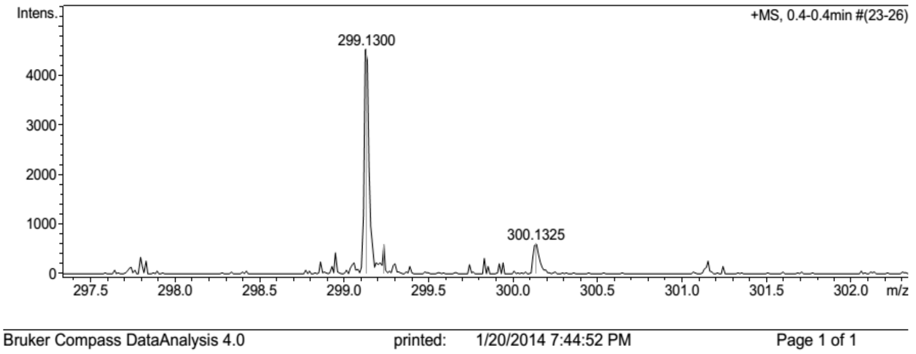

(E)-1-(4-Methoxyphenyl)-3-(2,5-dimethoxyphenyl)prop-2-en-1-one (**a13**)

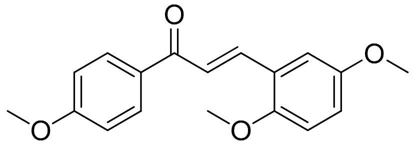

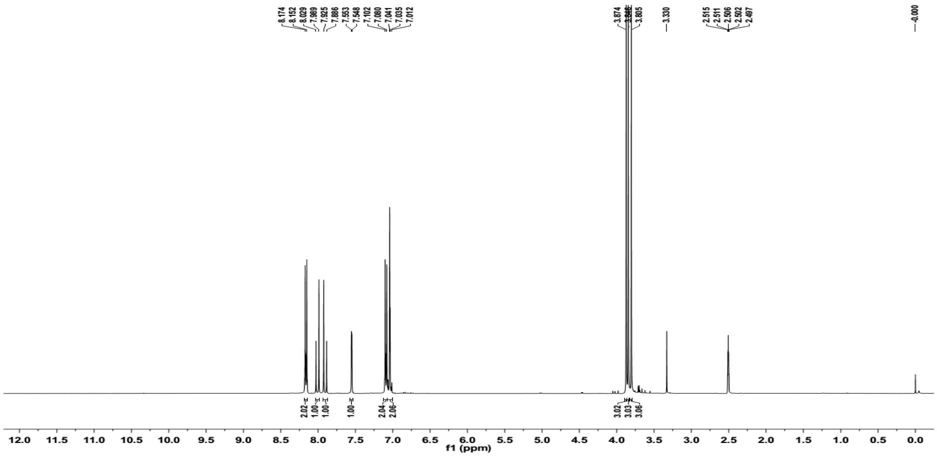

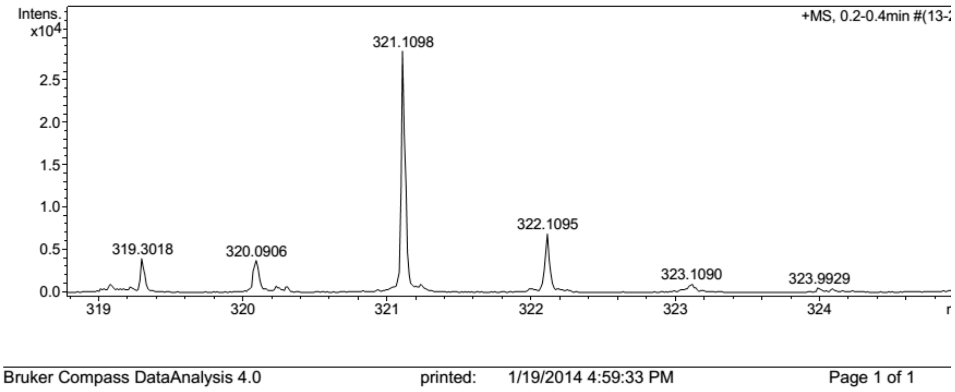

(E)-1-(4-Methoxyphenyl)-3-(2,6-dimethoxyphenyl)prop-2-en-1-one (**a14**)

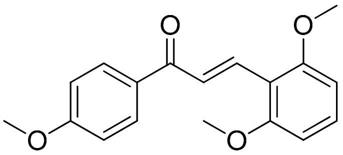

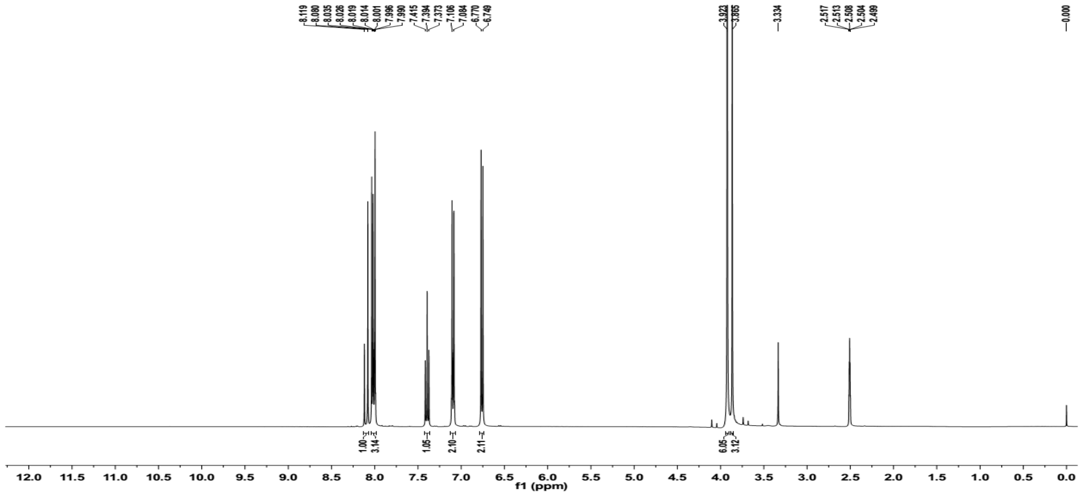

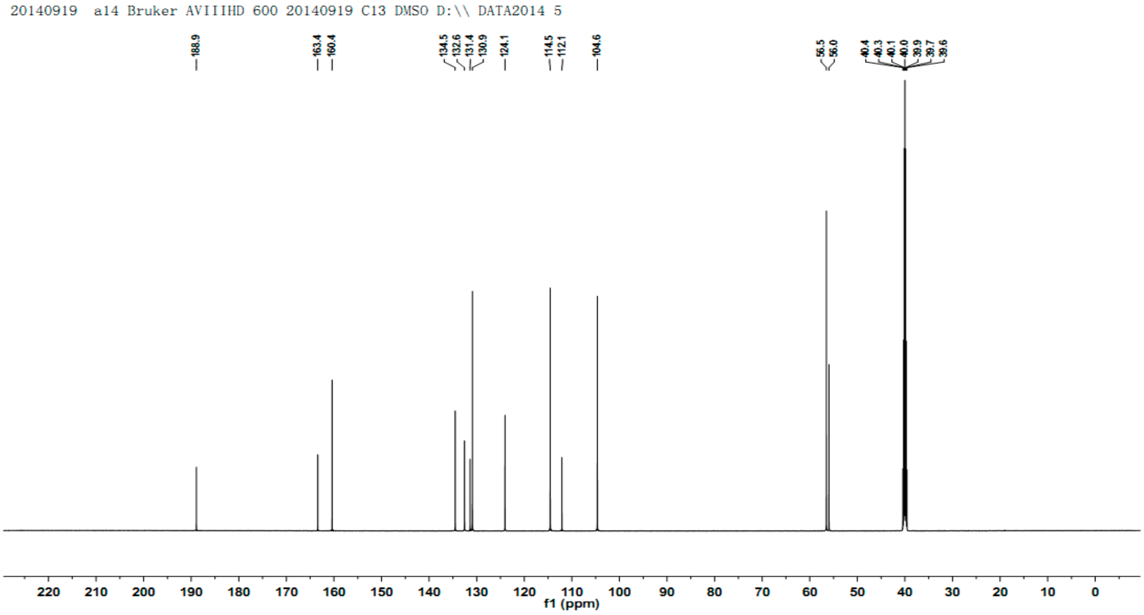

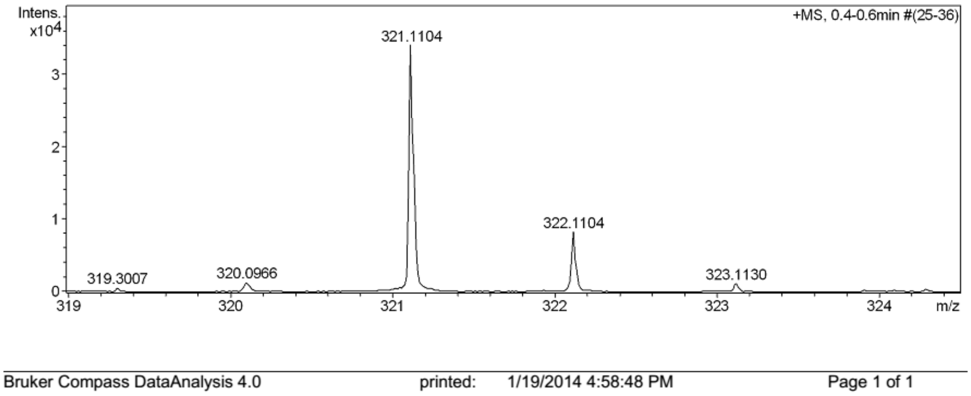

(E)-1-(4-Methoxyphenyl)-3-(3,4-dimethoxyphenyl)prop-2-en-1-one (**a15**)

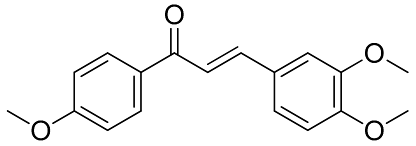

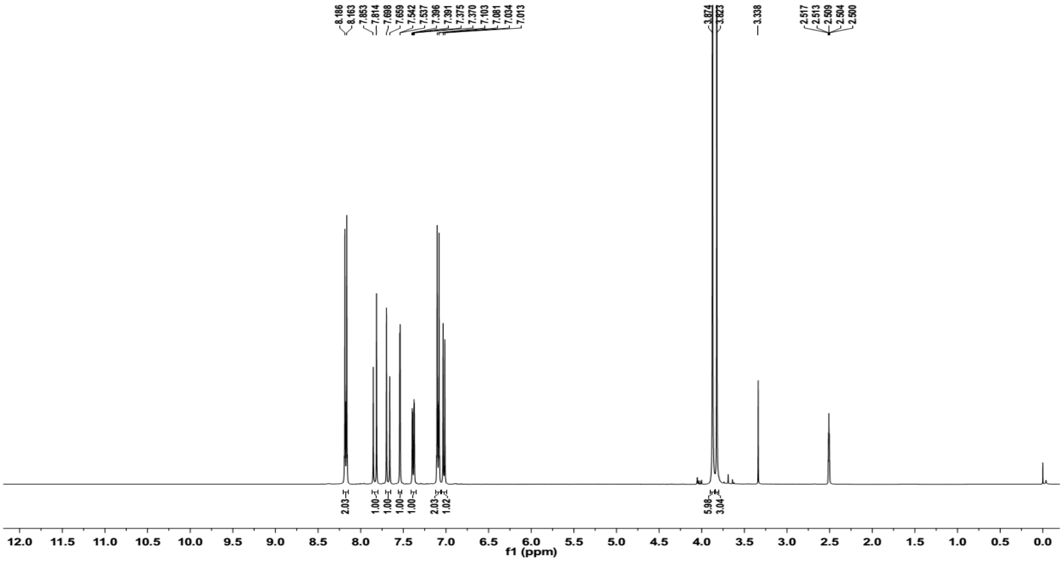

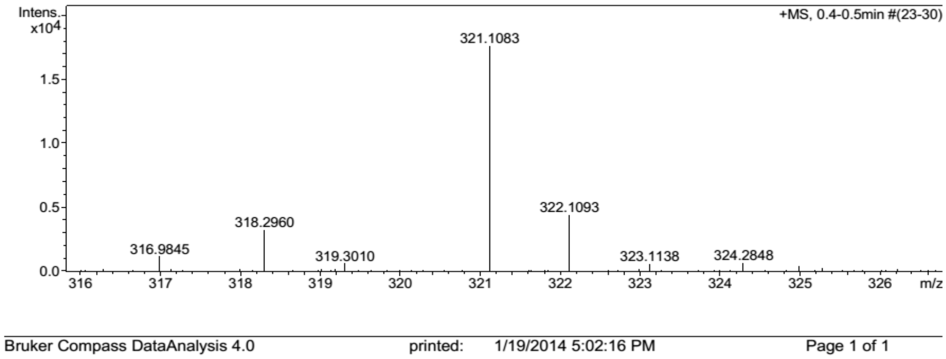

(E)-1-(4-Methoxyphenyl)-3-(3,5-dimethoxyphenyl)prop-2-en-1-one (**a16**)

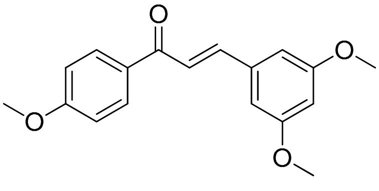

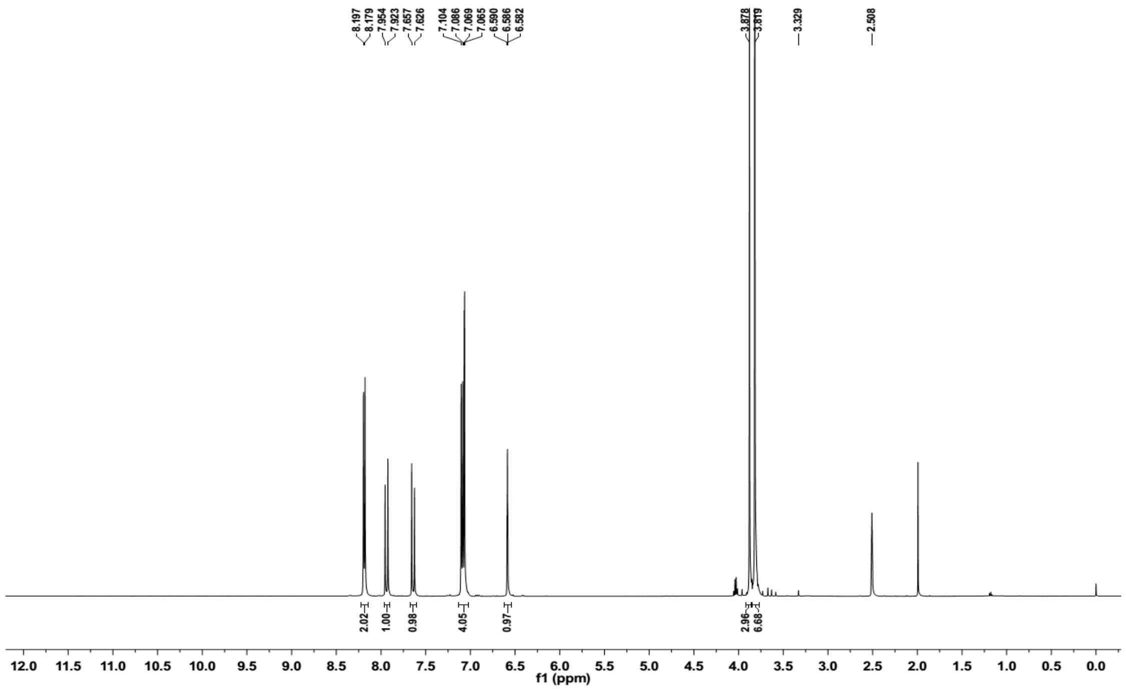

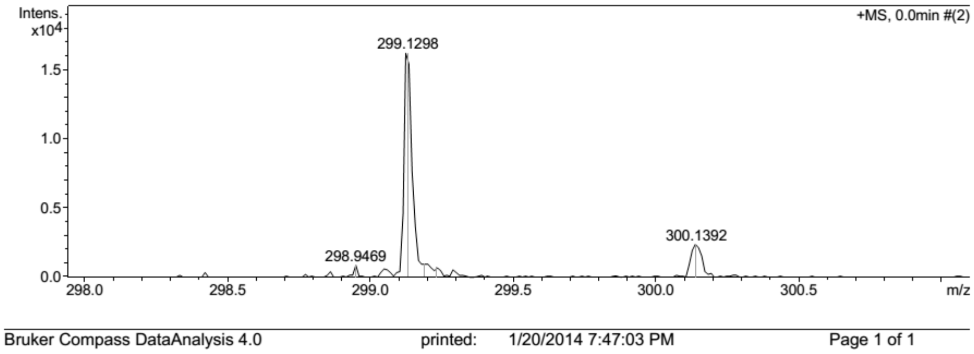

(E)-1-(4-Methoxyphenyl)-3-(3-(benzyloxy)-4-methoxyphenyl)prop-2-en-1-one (**a17**)

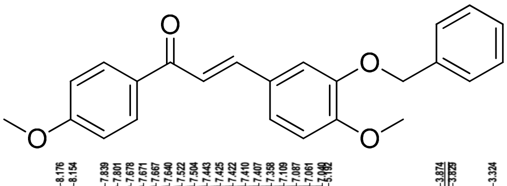

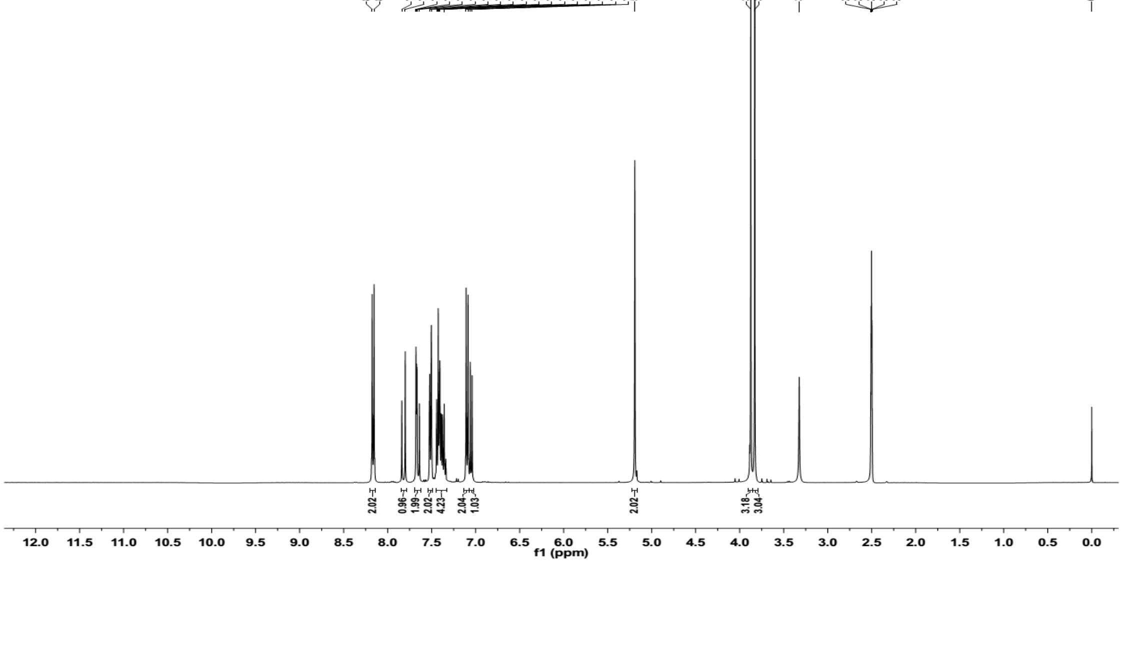

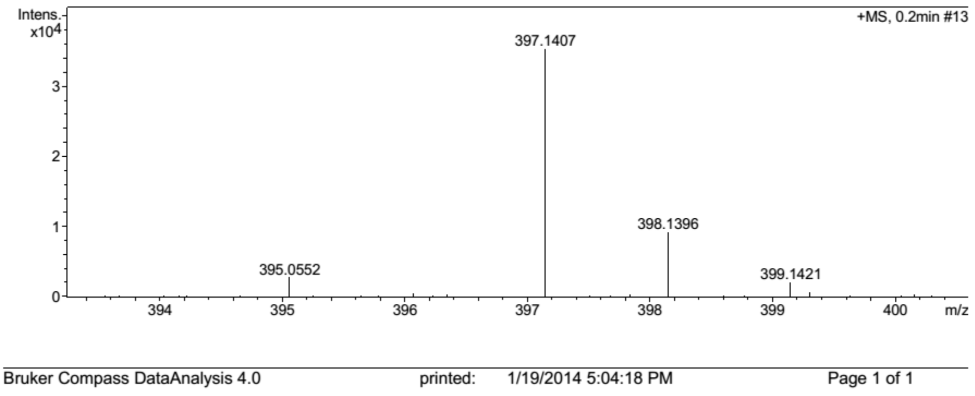

(E)-1-(4-Methoxyphenyl)-3-(2,3,4-trimethoxyphenyl)prop-2-en-1-one (**a18**)

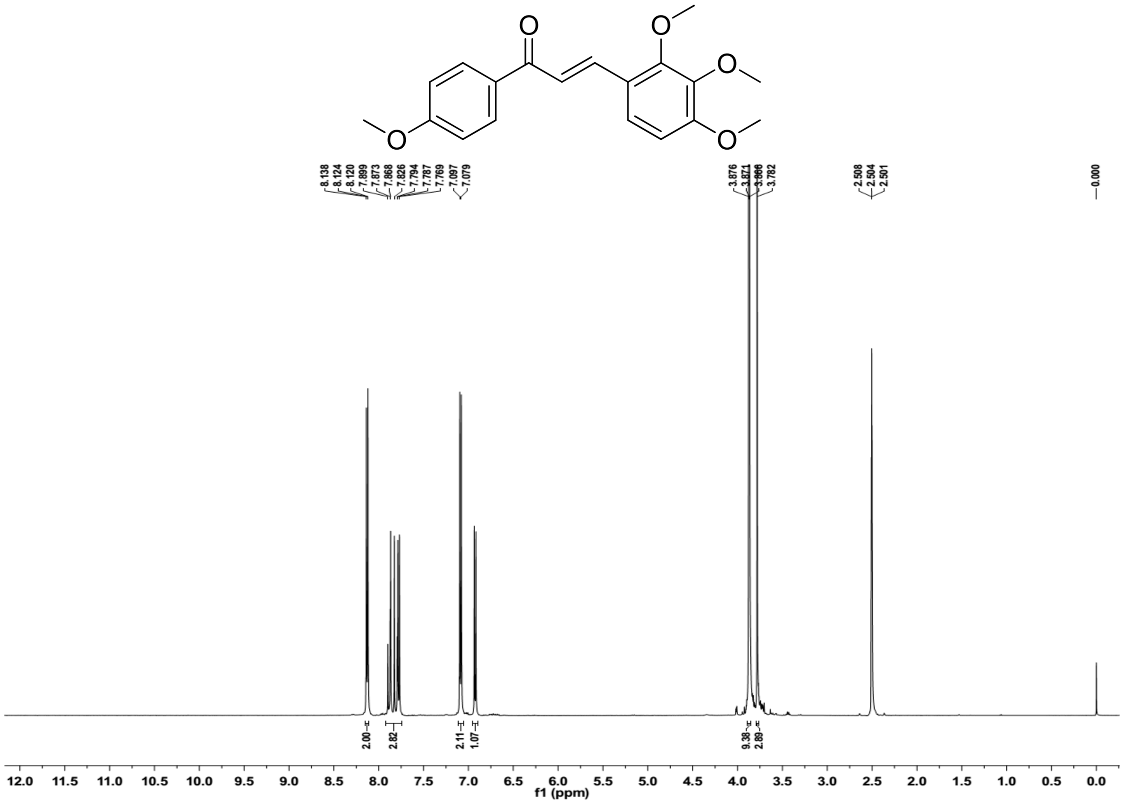

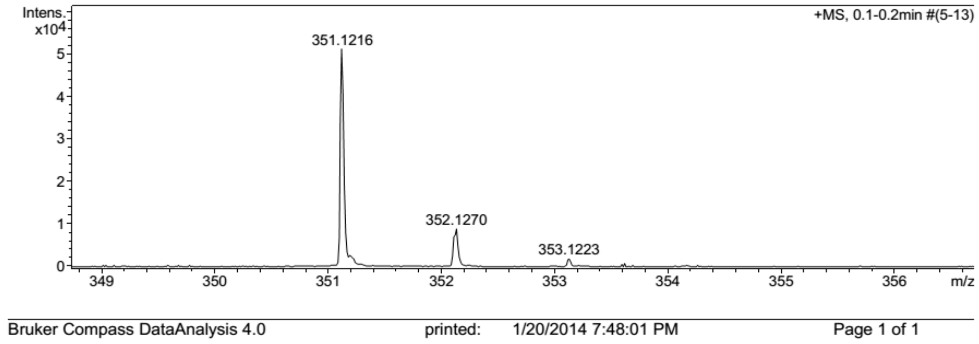

(E)-1-(4-Methoxyphenyl)-3-(2,4,5-trimethoxyphenyl)prop-2-en-1-one (**a19**)

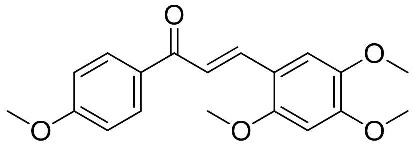

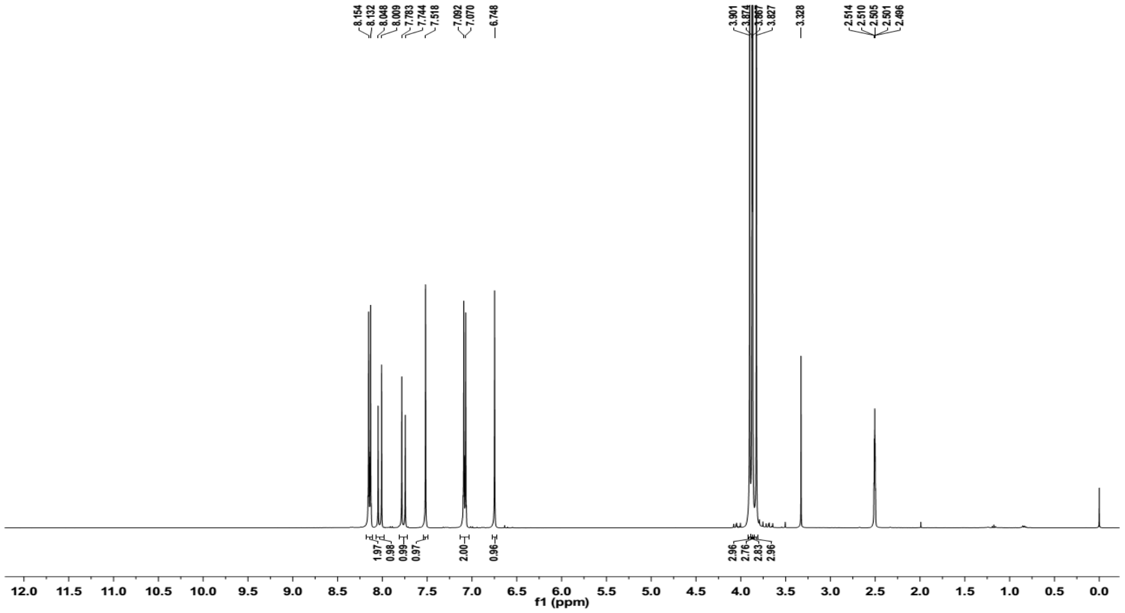

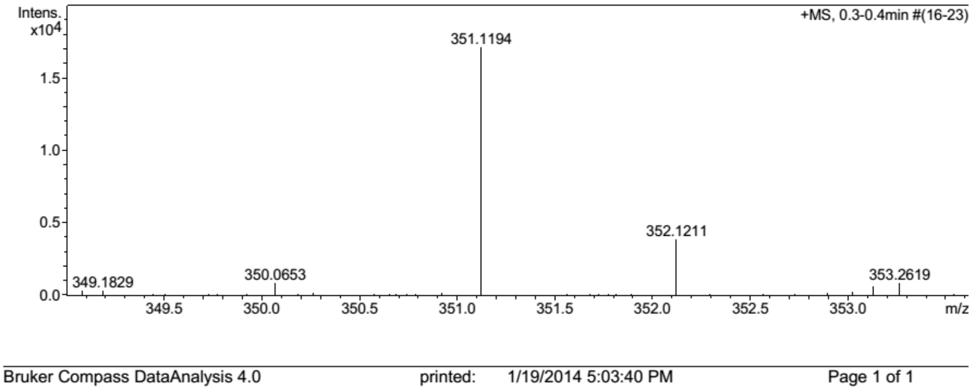

(E)-1-(4-Methoxyphenyl)-3-(3,4,5-trimethoxyphenyl)prop-2-en-1-one (**a20**)

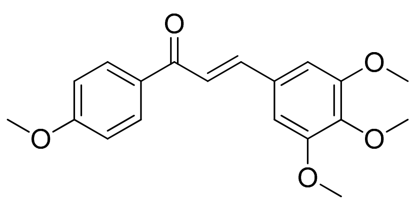

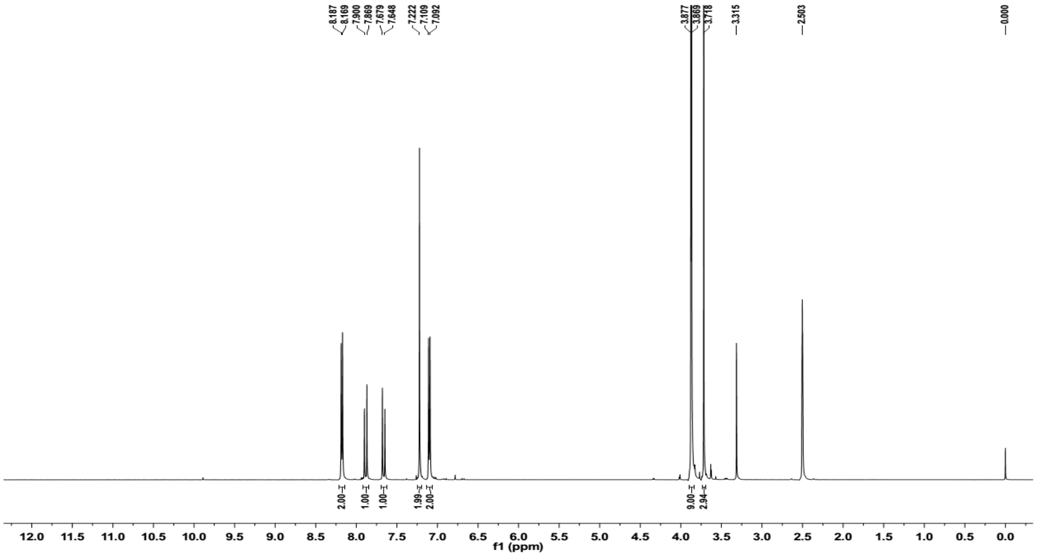

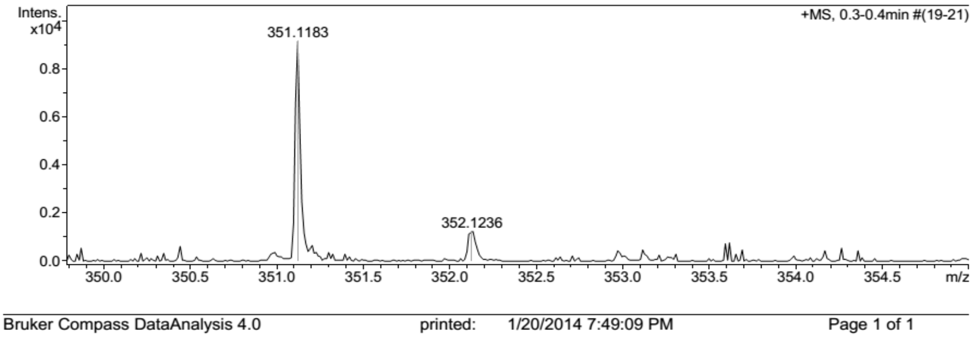

(E)-1-Phenyl-3-(2,6-dimethoxyphenyl)prop-2-en-1-one (**b1**)

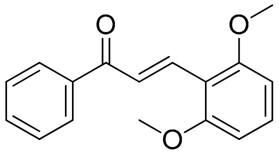

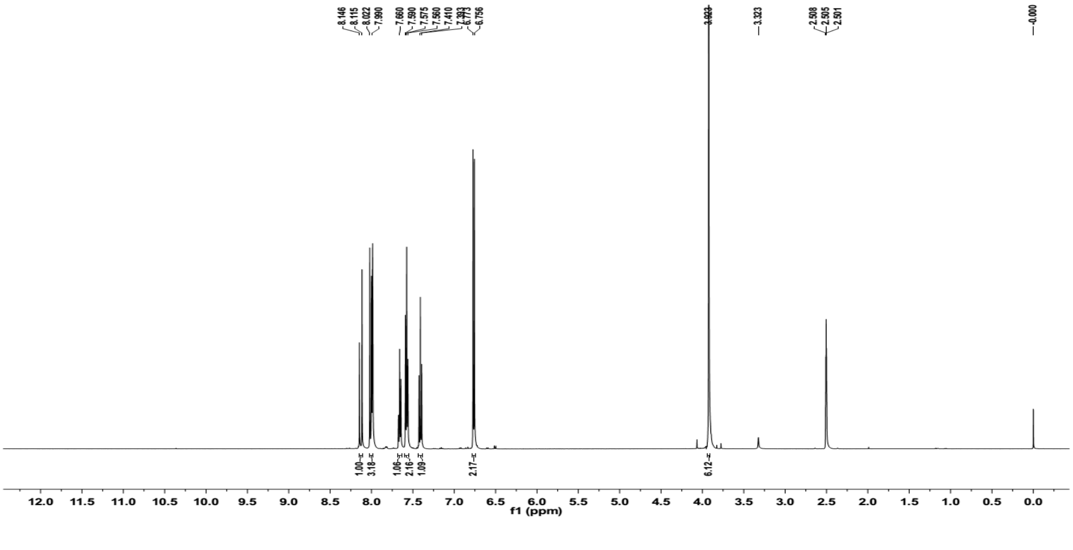

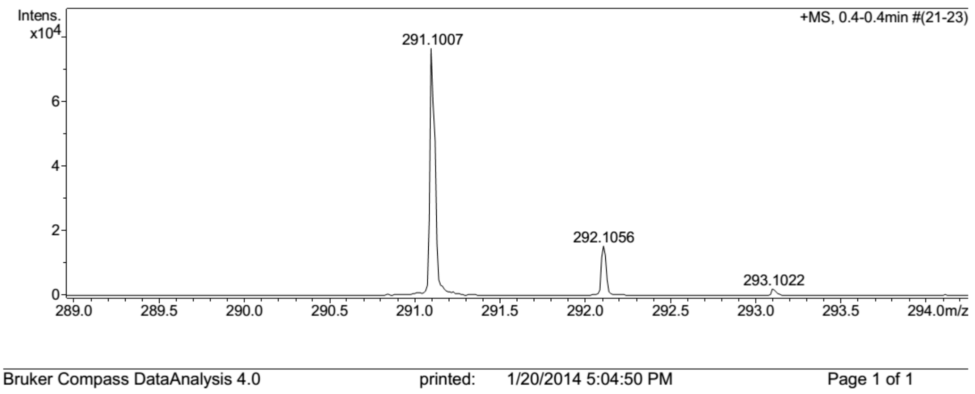

(E)-1-(2-Methylphenyl)-3-(2,6-dimethoxyphenyl)prop-2-en-1-one (**b2**)

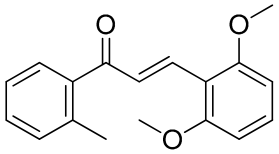

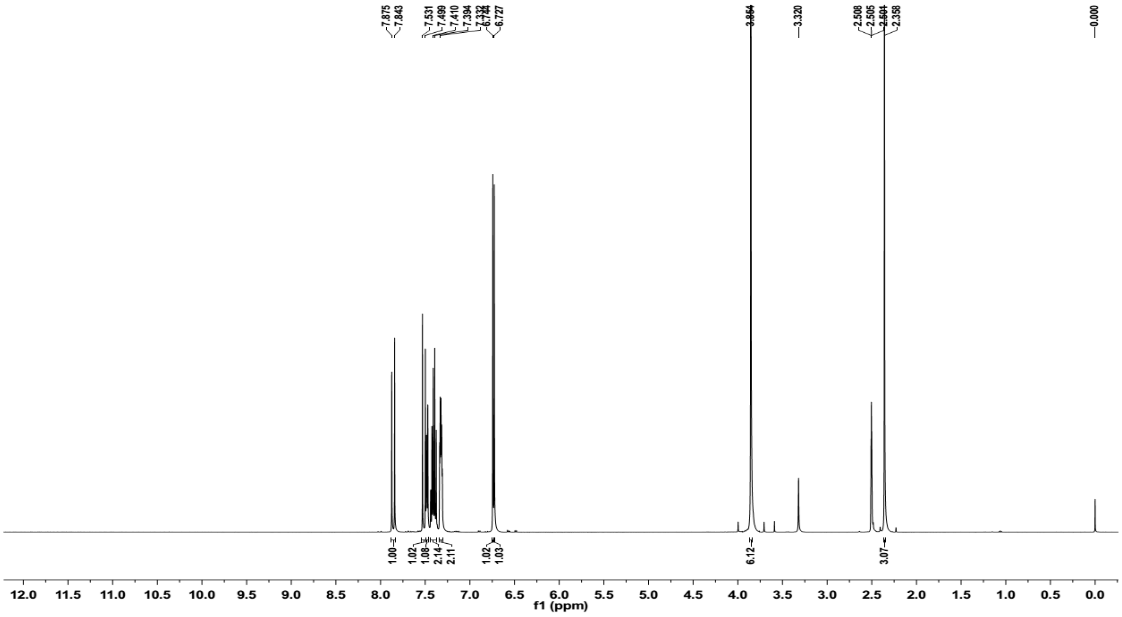

20140919 b2 Bruker AVIIIHD 600 20140919 C13 DMSO D:\\ DATA2014 6

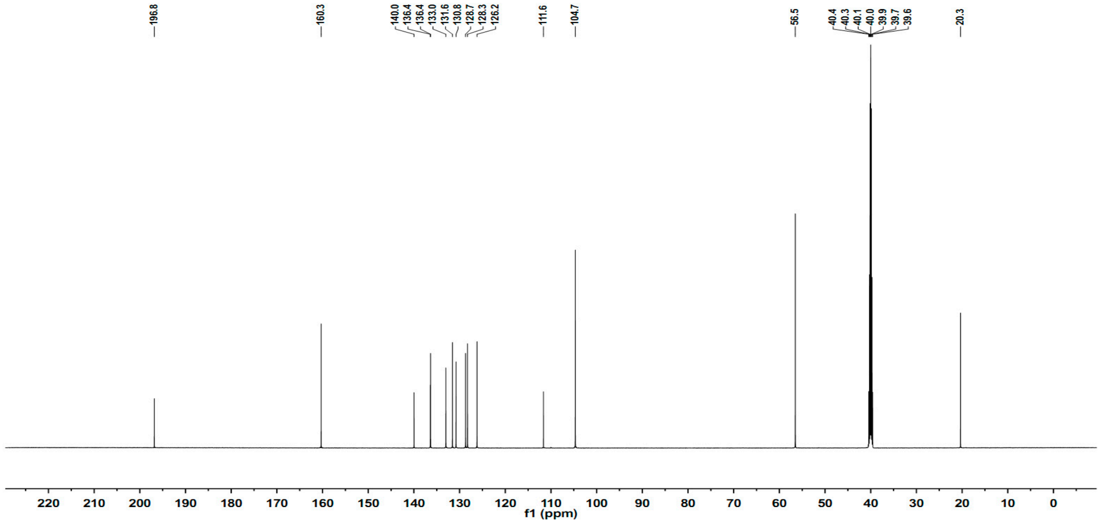

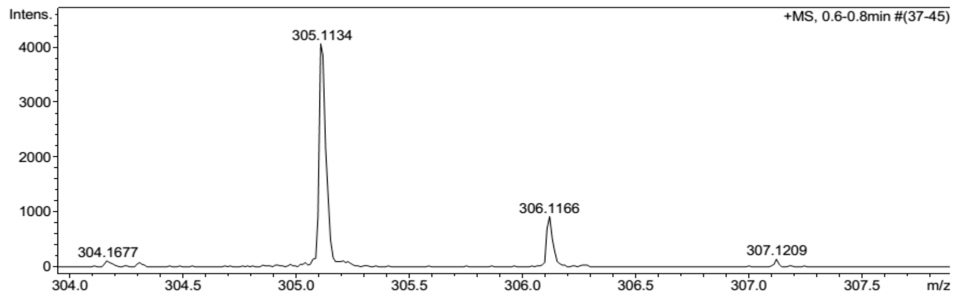

Bruker Compass DataAnalysis 4.0 printed: 1/20/2014 2:14:02 PM Page 1 of 1

(E)-1-(3-Methylphenyl)-3-(2,6-dimethoxyphenyl)prop-2-en-1-one (**b3**)

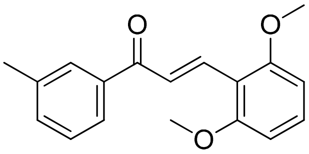

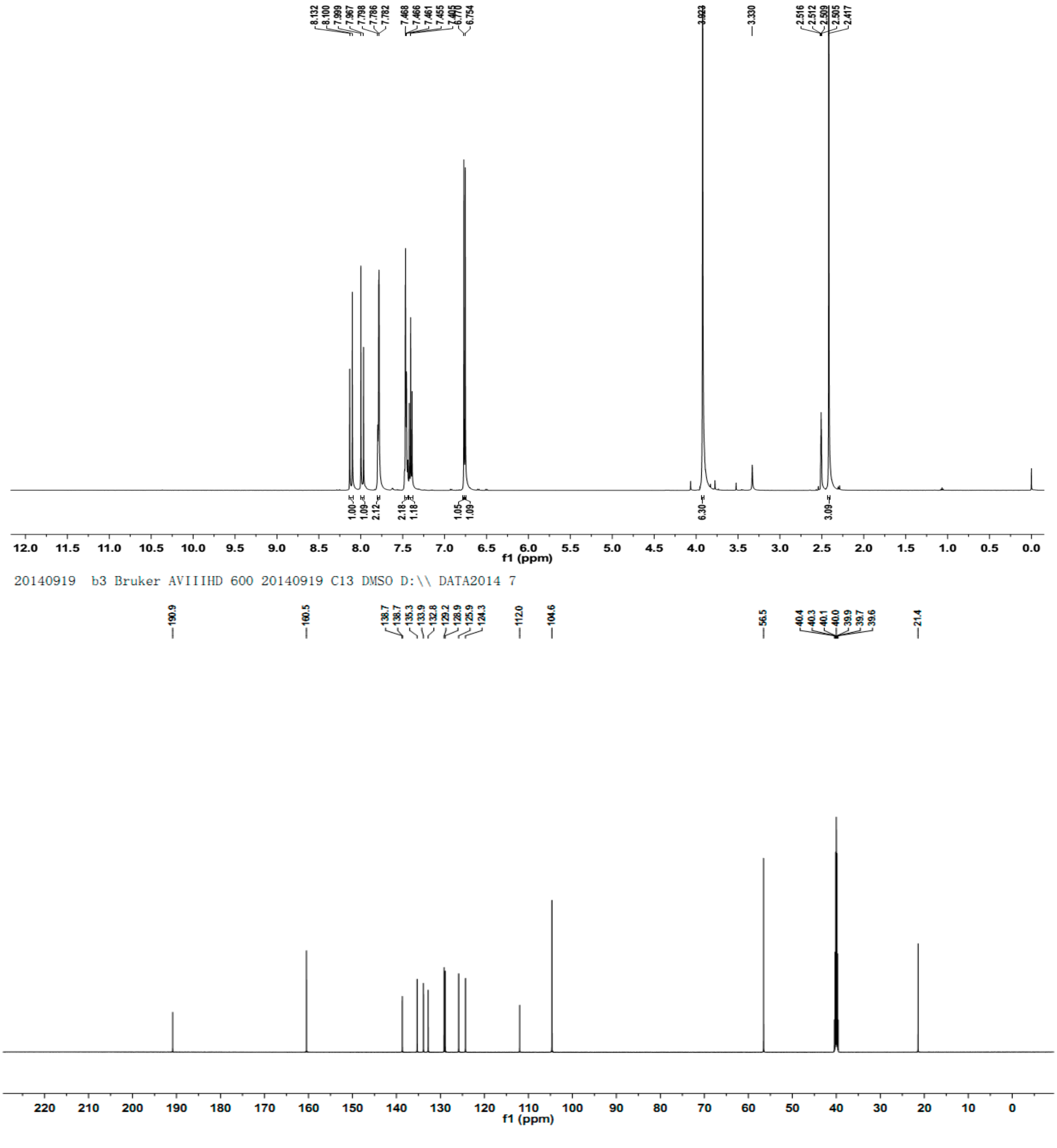

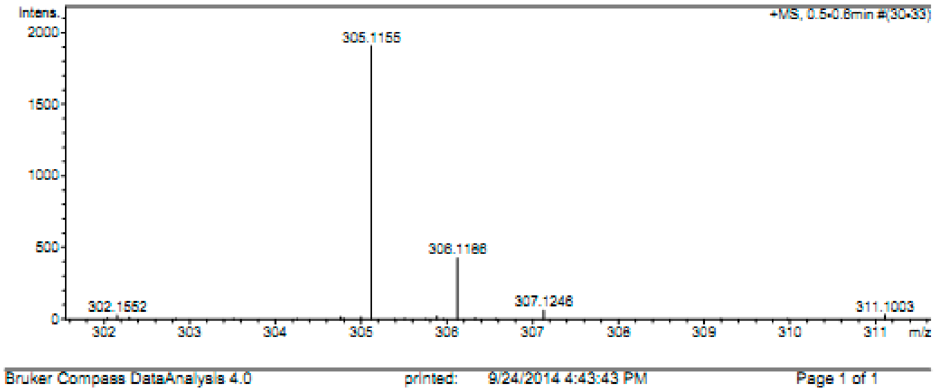

(E)-1-(4-Methylphenyl)-3-(2,6-dimethoxyphenyl)prop-2-en-1-one (**b4**)

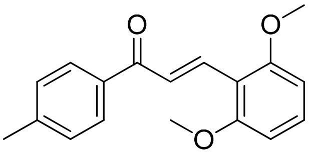

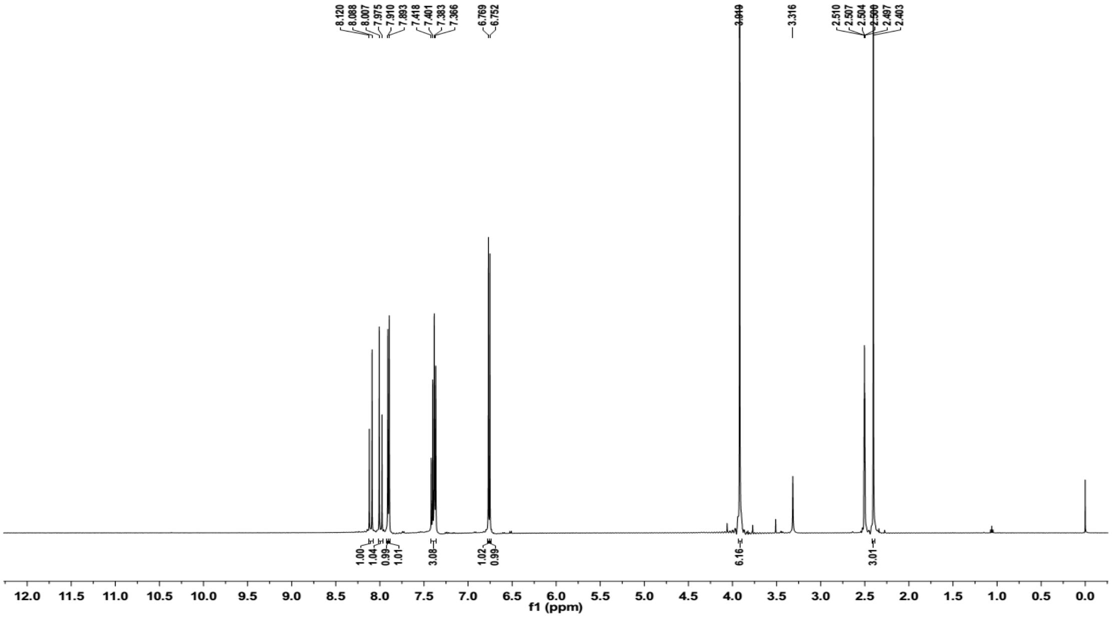

20140919 b4 Bruker AVIIIHD 600 20140919 C13 DMSO D:\\ DATA2014 8

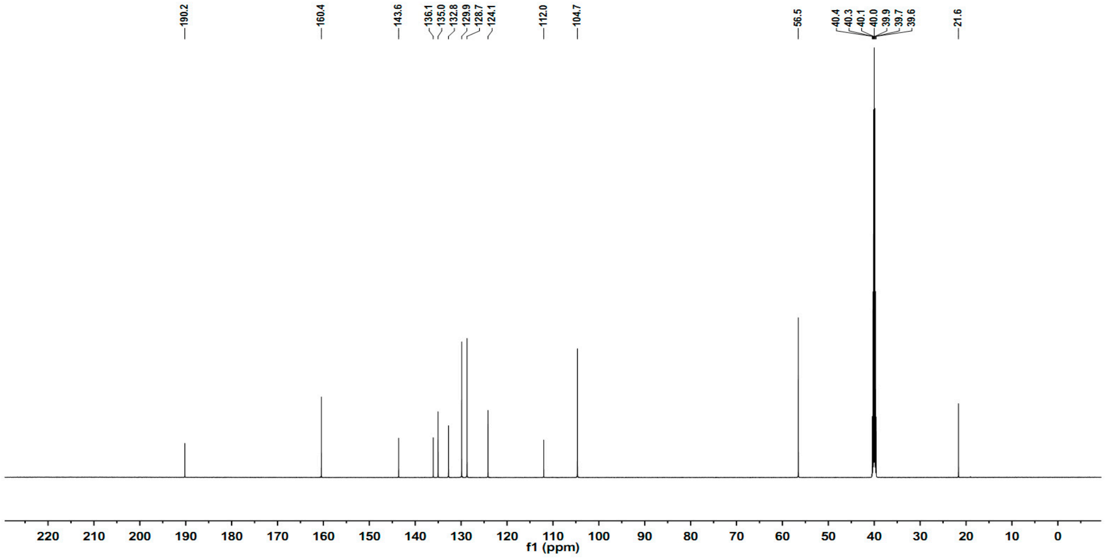

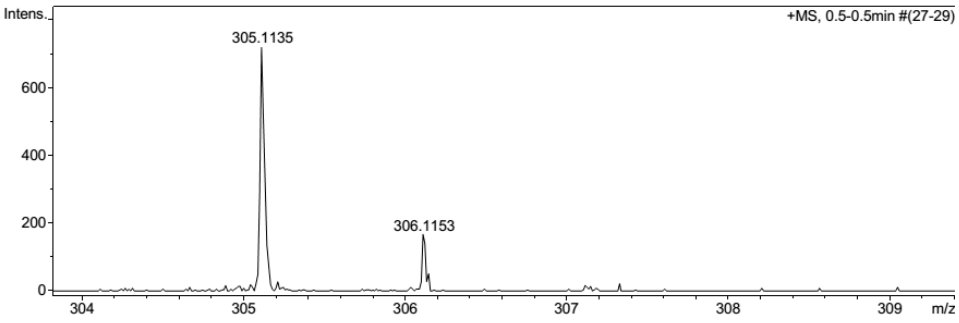

Bruker Compass DataAnalysis 4.0 printed: 1/20/2014 2:20:00 PM Page 1 of 1

(E)-1-(3,4-Dimethylphenyl)-3-(2,6-dimethoxyphenyl)prop-2-en-1-one (**b5**)

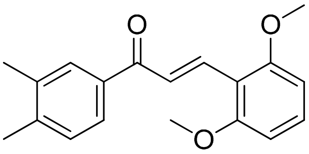

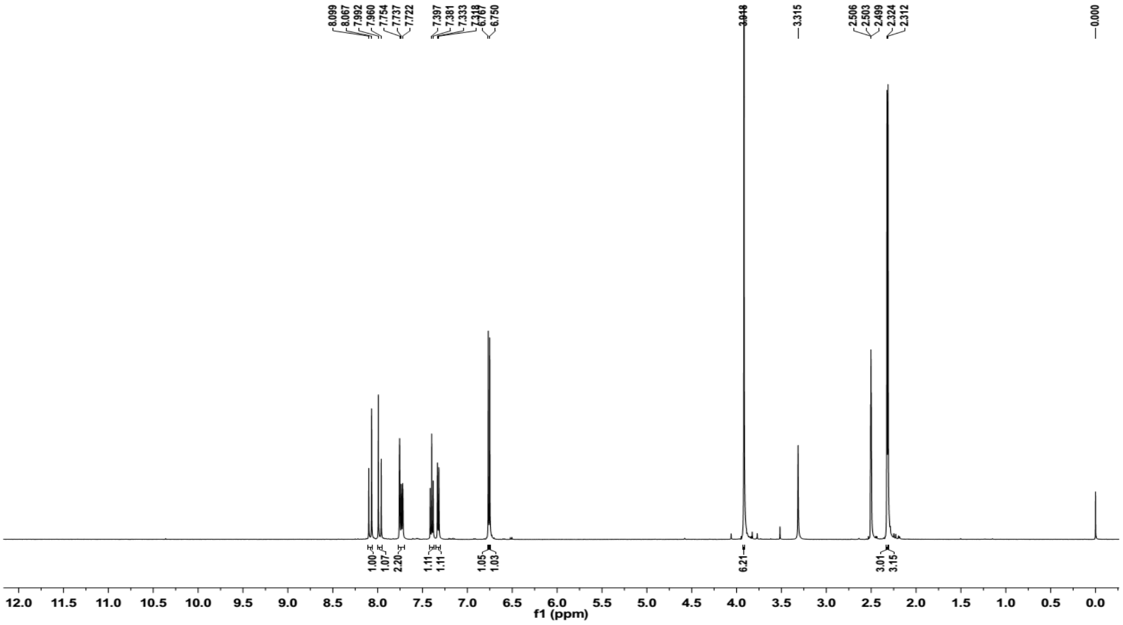

20140919 b5 Bruker AVIIIHD 600 20140919 C13 DMSO D:\\ DATA2014 9

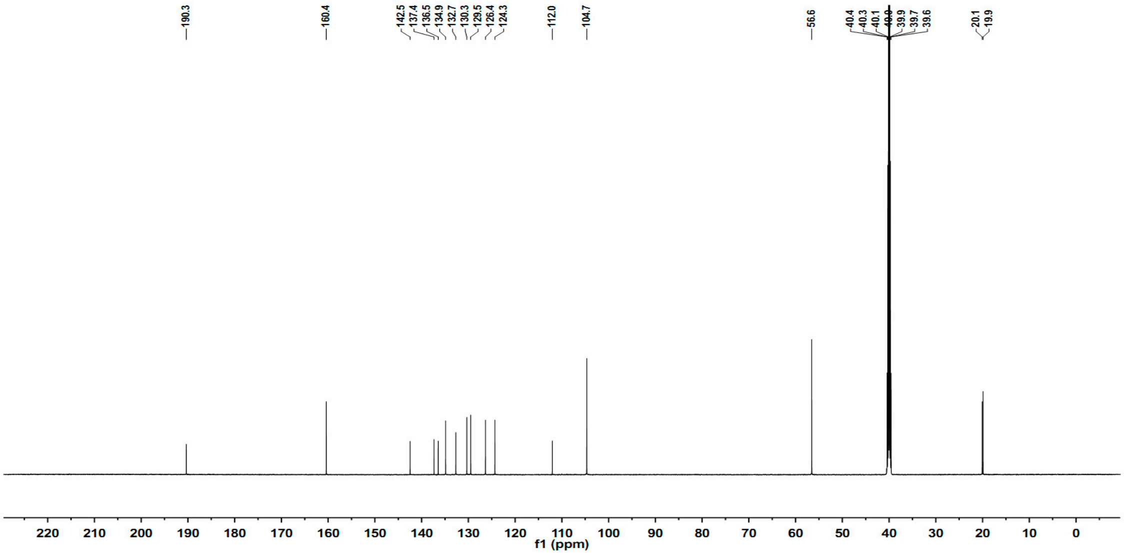

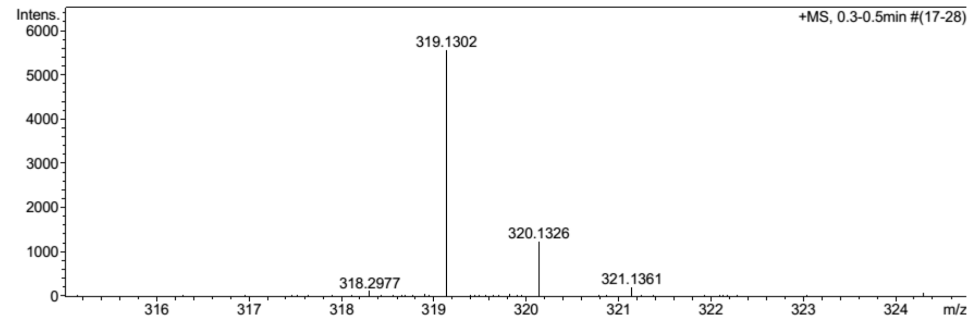

Bruker Compass DataAnalysis 4.0 printed: 1/20/2014 2:21:05 PM Page 1 of 1

(E)-1-(2-Methoxyphenyl)-3-(2,6-dimethoxyphenyl)prop-2-en-1-one (**b6**)

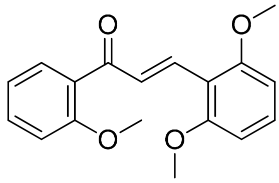

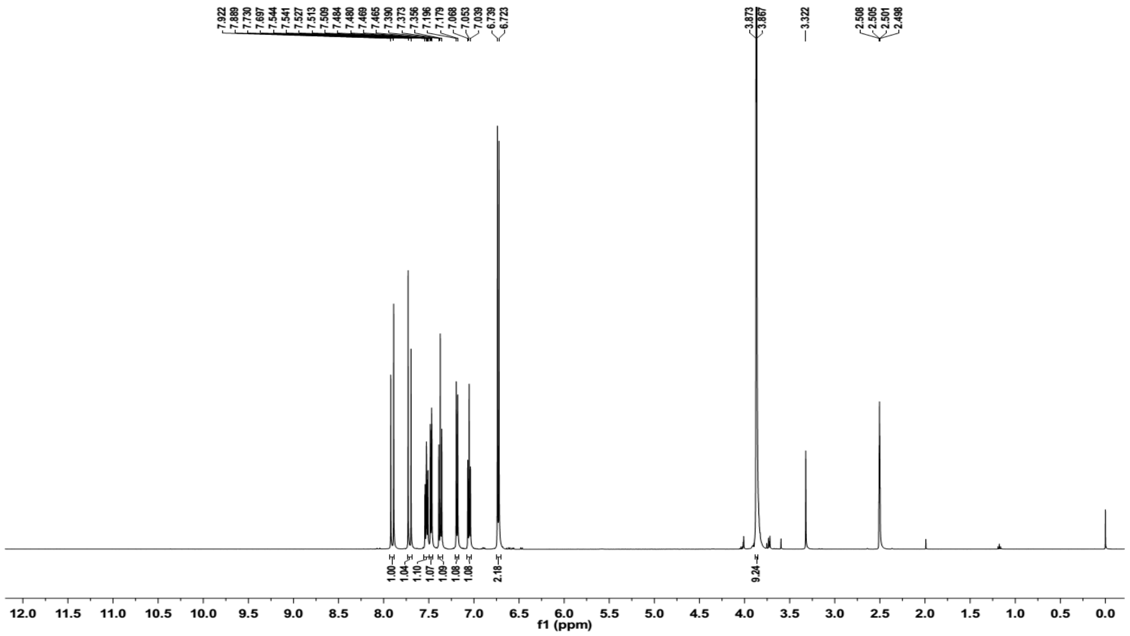

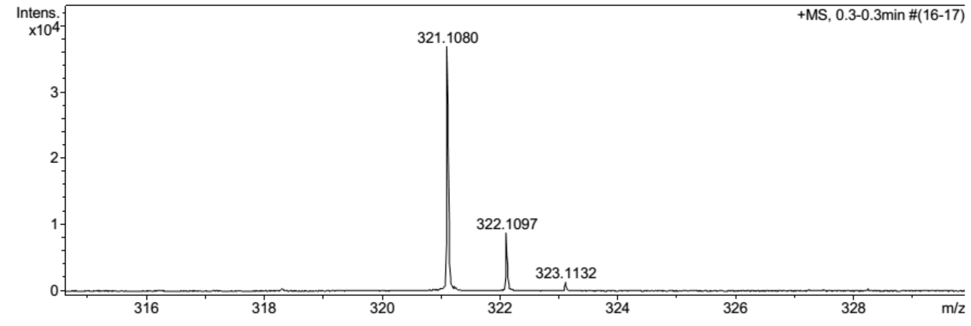

Bruker Compass DataAnalysis 4.0 printed: 1/20/2014 2:11:08 PM Page 1 of 1

(E)-1-(3-Methoxyphenyl)-3-(2,6-dimethoxyphenyl)prop-2-en-1-one (**b7**)

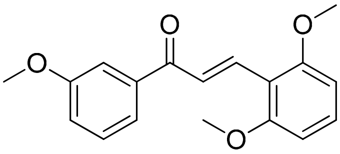

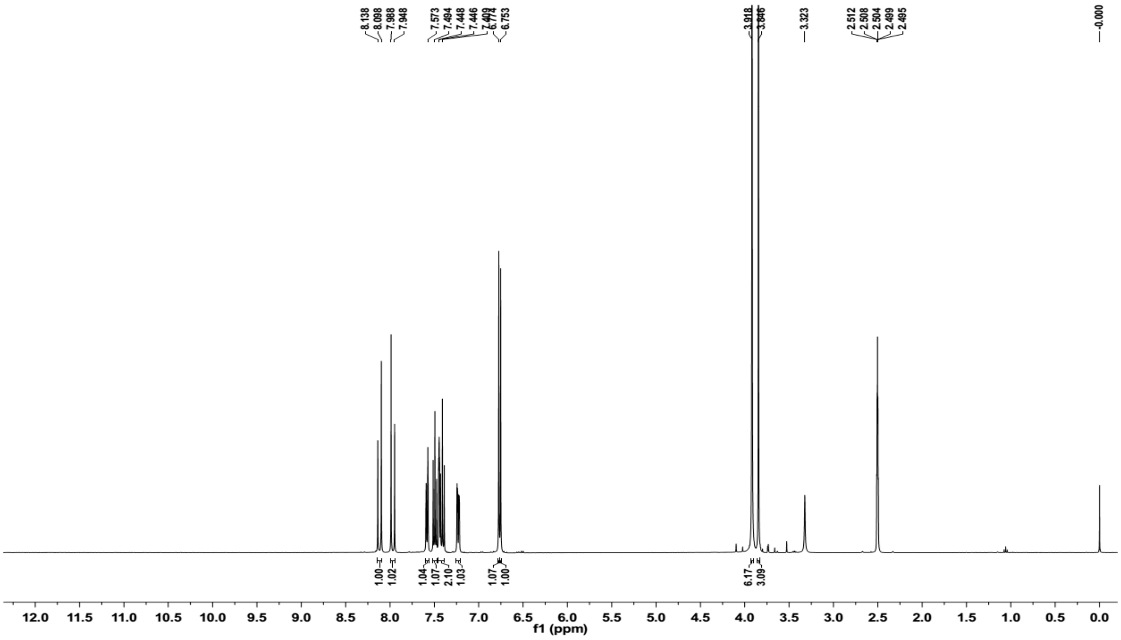

20140919 b7 Bruker AVIIIHD 600 20140919 C13 DMSO D:\\ DATA2014 10

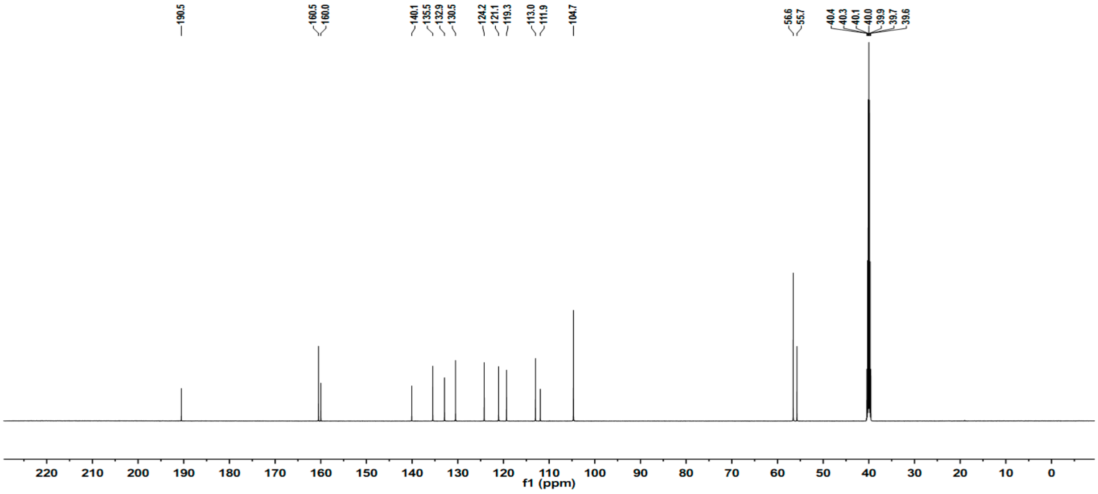

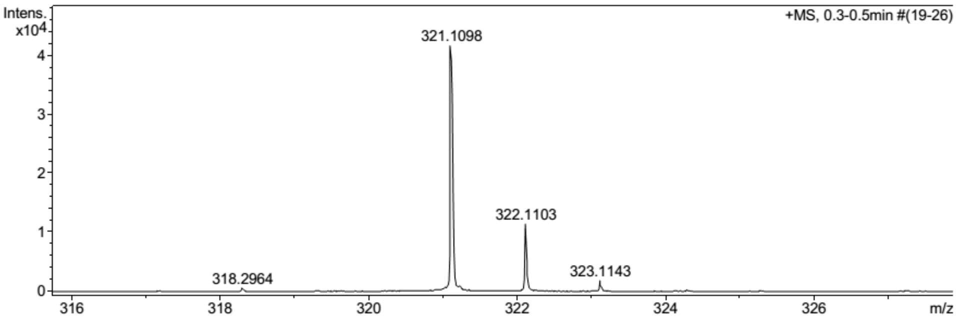

Bruker Compass DataAnalysis 4.0 printed: 1/20/2014 2:10:14 PM Page 1 of 1

(E)-1-(2-Ethoxyphenyl)-3-(2,6-dimethoxyphenyl)prop-2-en-1-one (**b8**)

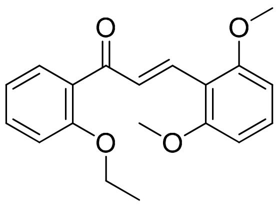

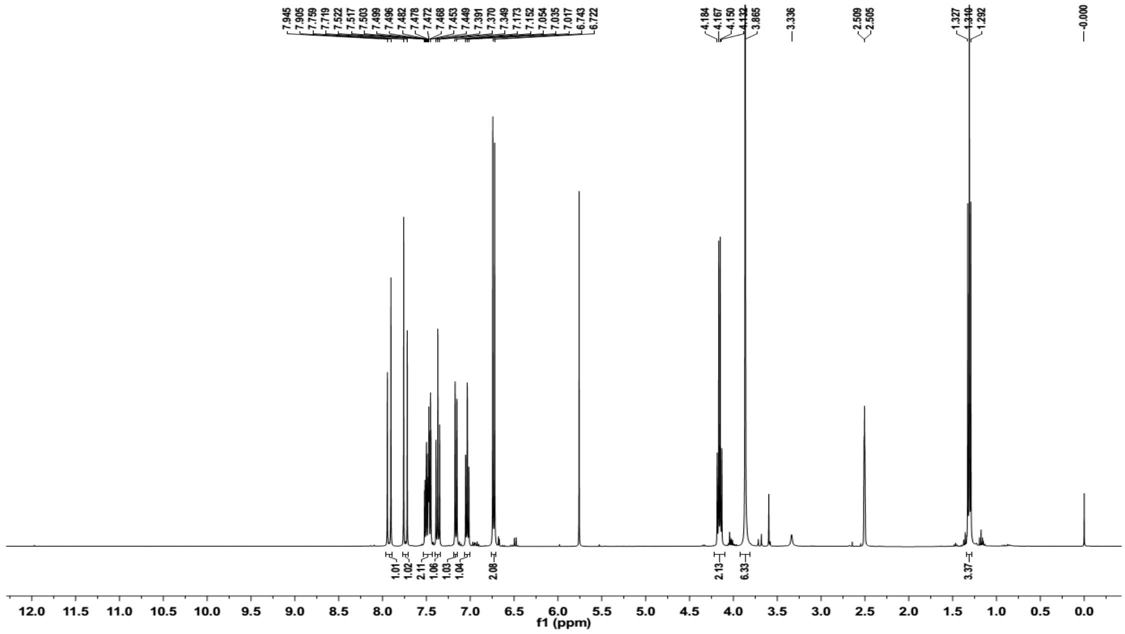

20140919 b8 Bruker AVIIIHD 600 20140919 C13 DMSO D:\\ DATA2014 12

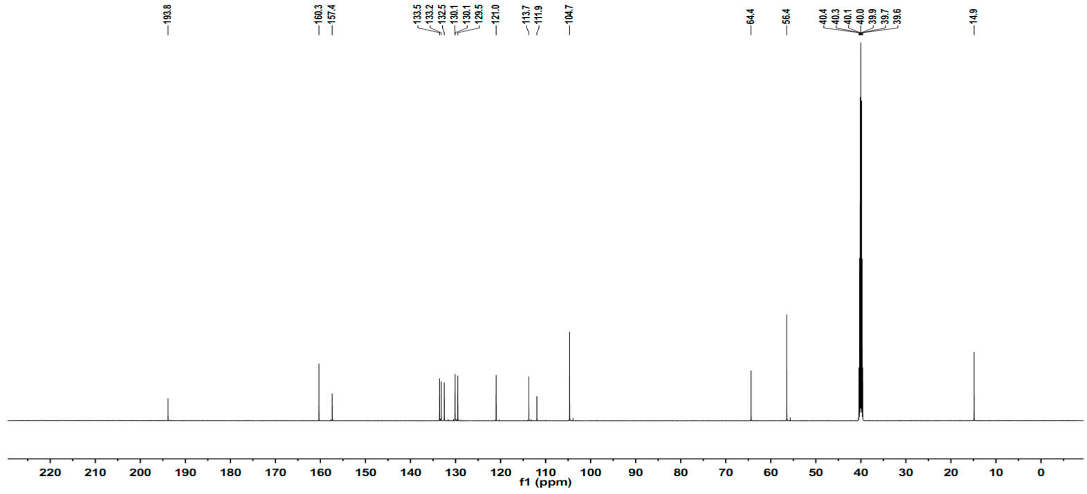

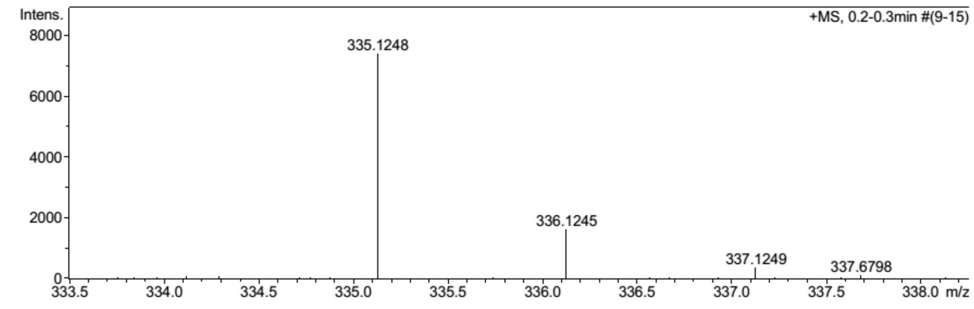

Bruker Compass DataAnalysis 4.0 printed: 1/27/2014 8:27:37 AM Page 1 of 1

(E)-1-(3-Ethoxyphenyl)-3-(2,6-dimethoxyphenyl)prop-2-en-1-one (**b9**)

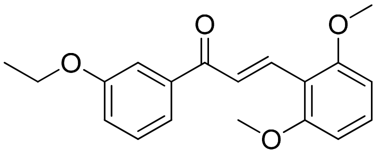

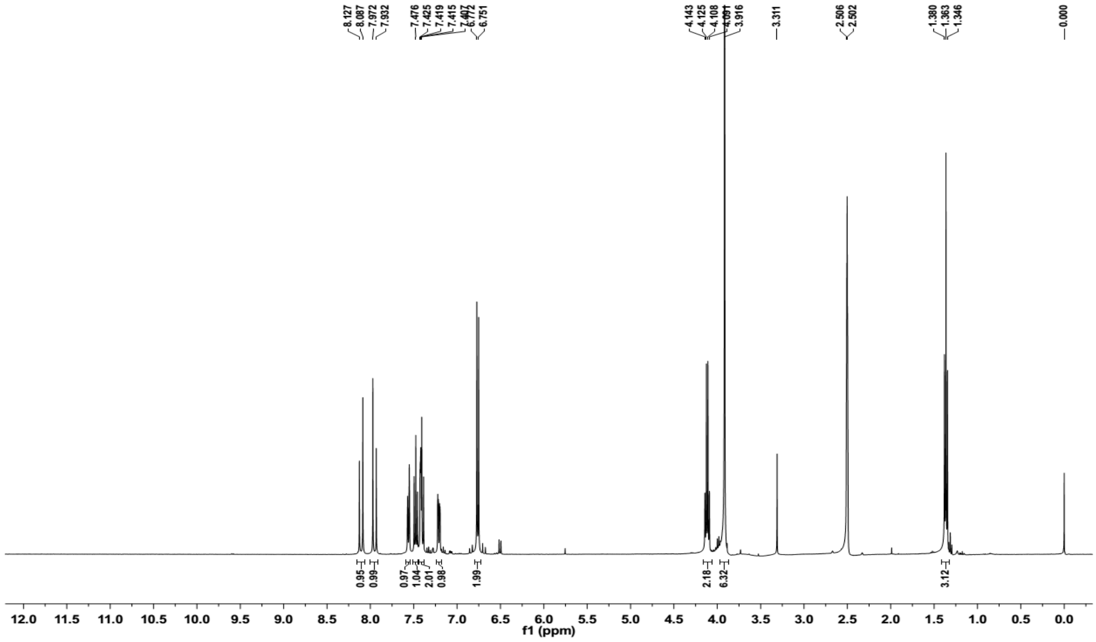

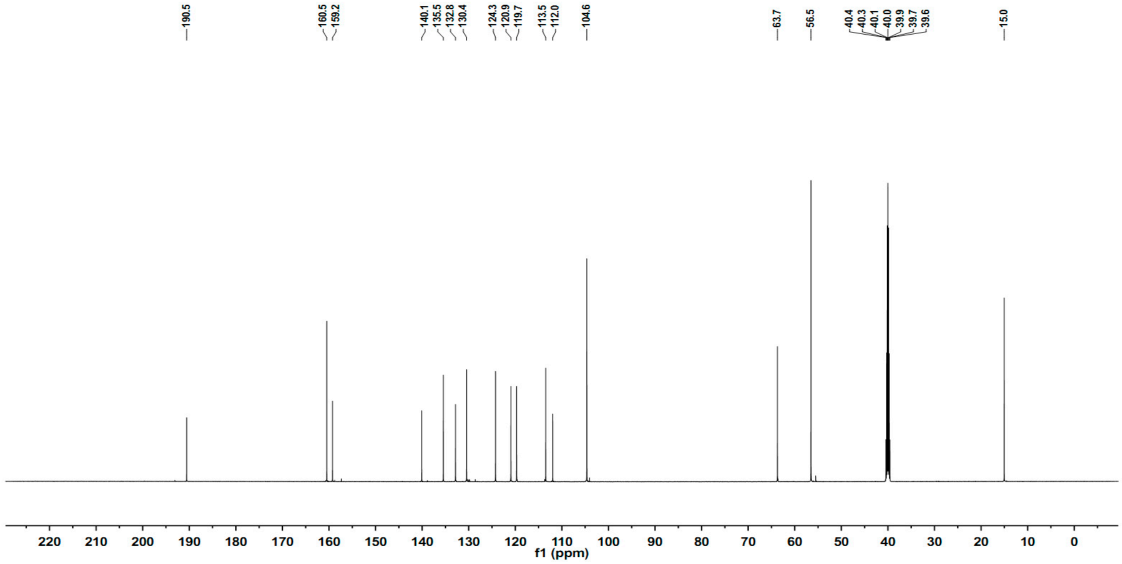

20140919 b9 Bruker AVIIIHD 600 20140919 C13 DMSO D:\\ DATA2014 13

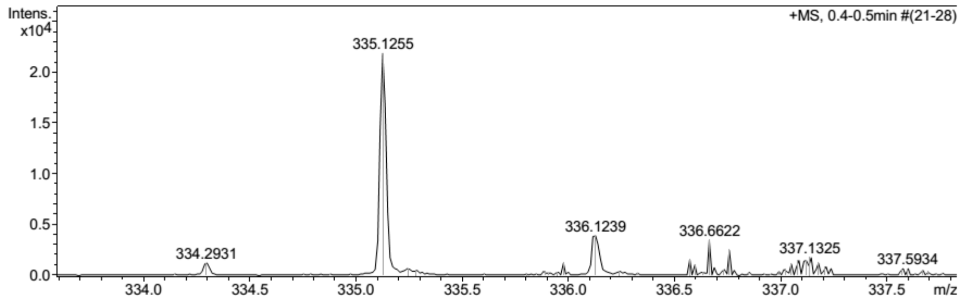

Bruker Compass DataAnalysis 4.0 printed: 1/27/2014 8:25:19 AM Page 1 of 1

(E)-1-(4-Ethoxyphenyl)-3-(2,6-dimethoxyphenyl)prop-2-en-1-one (**b10**)

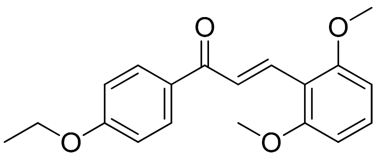

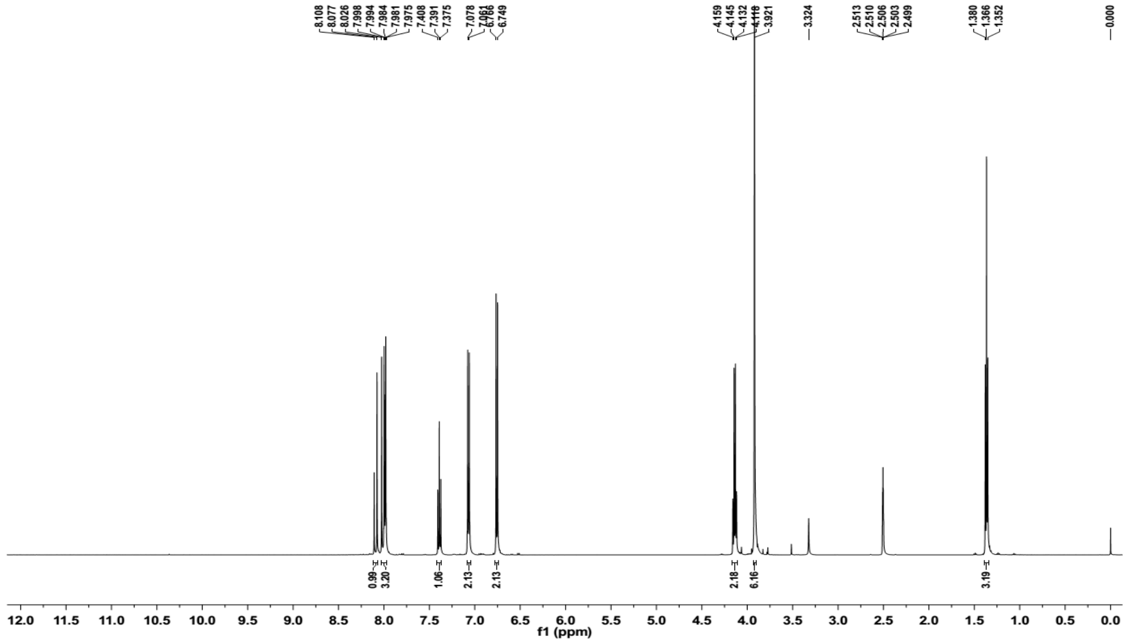

20140919 b10 Bruker AVIIIHD 600 20140919 C13 DMSO D:\\ DATA2014 14

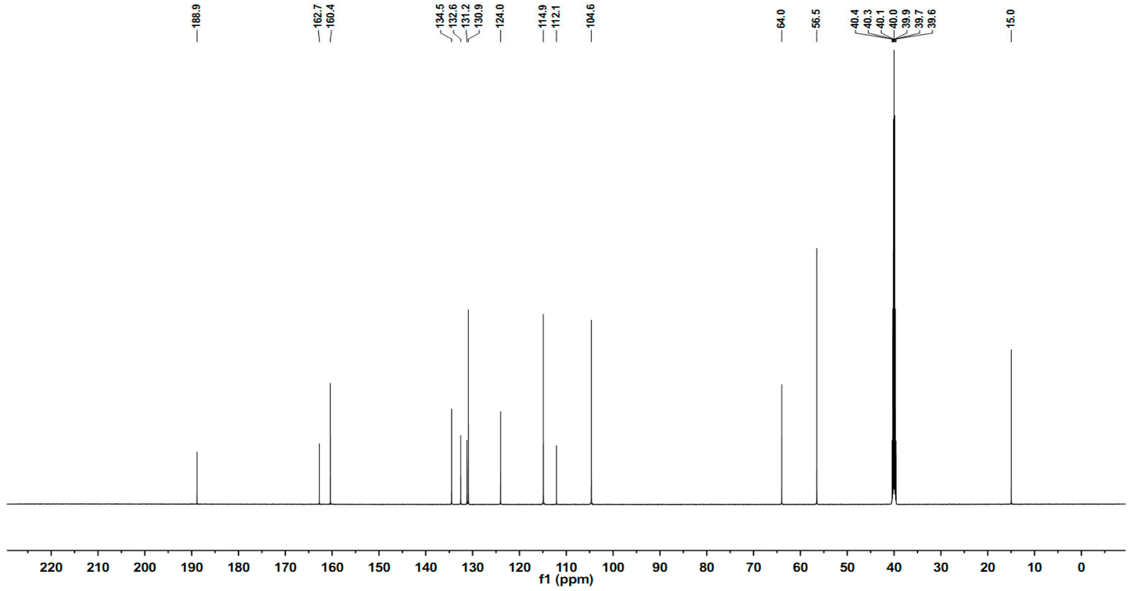

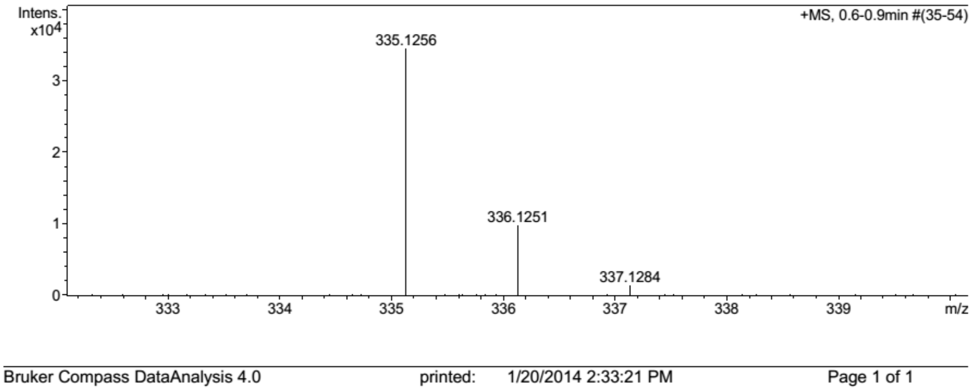

(E)-1-(2-(Benzyloxy)phenyl)-3-(2,6-dimethoxyphenyl)prop-2-en-1-one (**b11**)

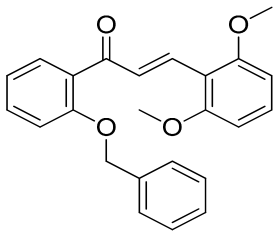

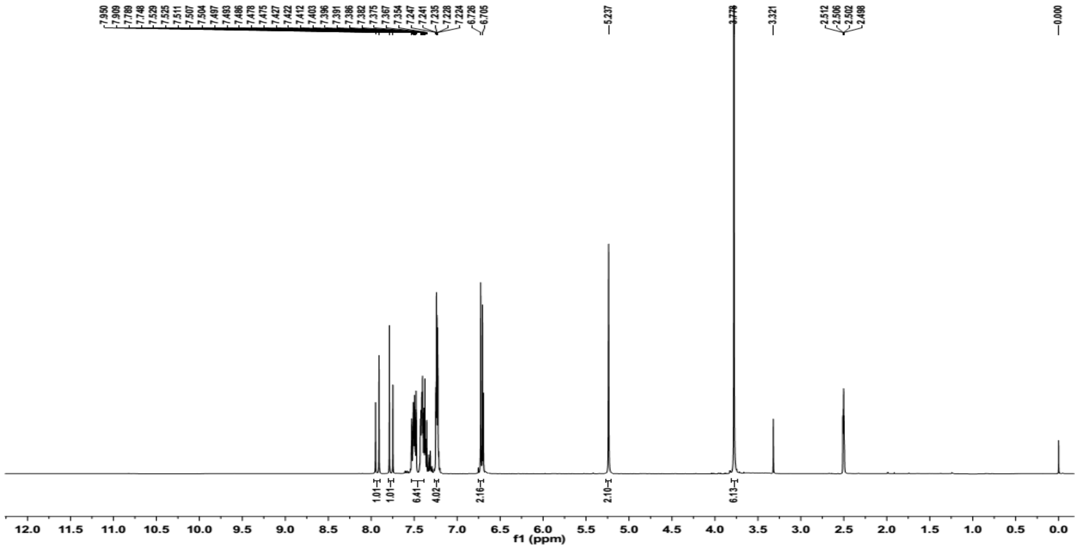

20140919 b11 Bruker AVIIIHD 600 20140919 C13 DMSO D:\\ DATA2014 15

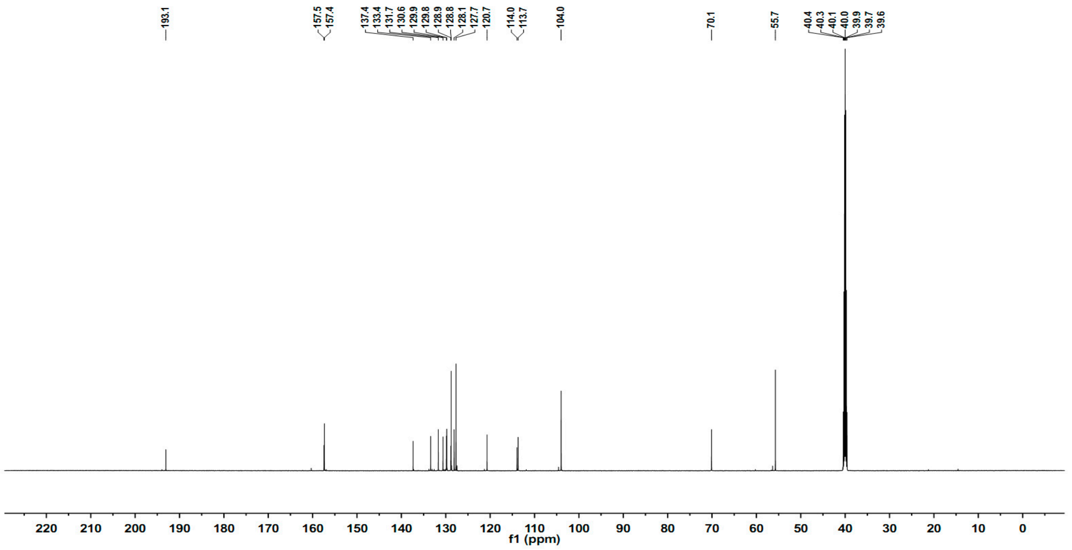

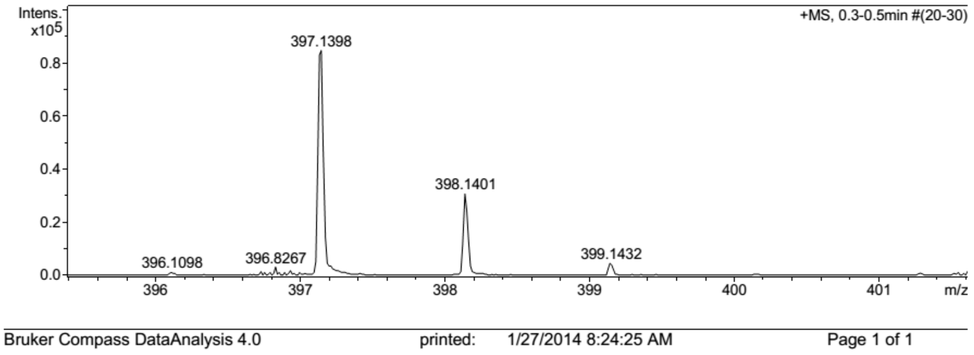

(E)-1-(3-(Benzyloxy)phenyl)-3-(2,6-dimethoxyphenyl)prop-2-en-1-one (**b12**)

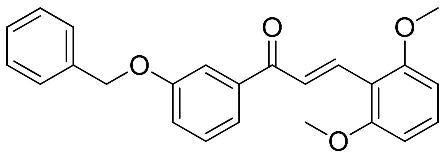

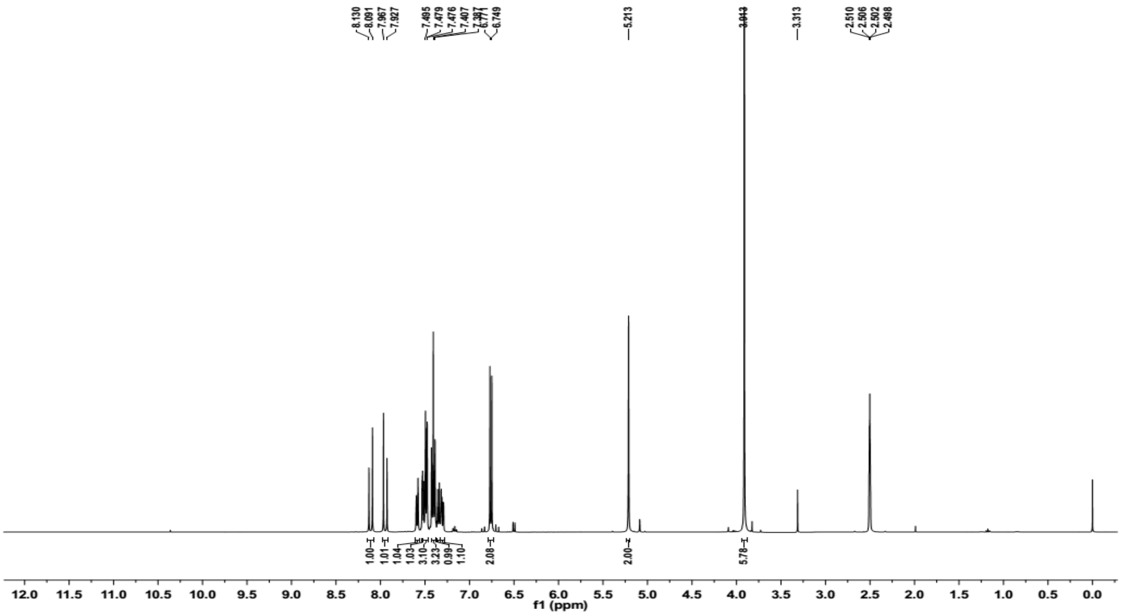

20140919 b12 Bruker AVIIIHD 600 20140919 C13 DMSO D:\\ DATA2014 16

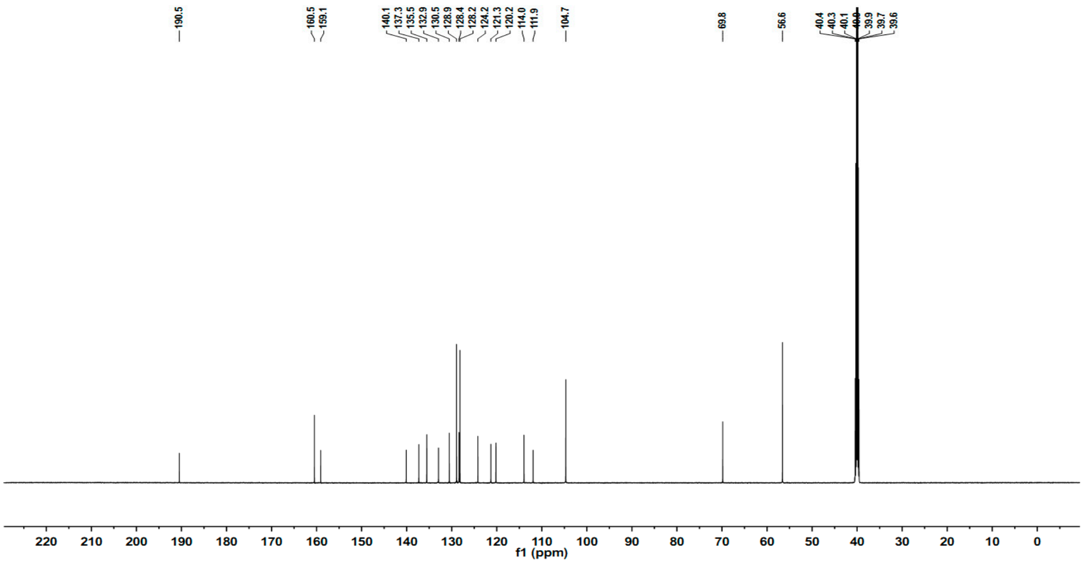

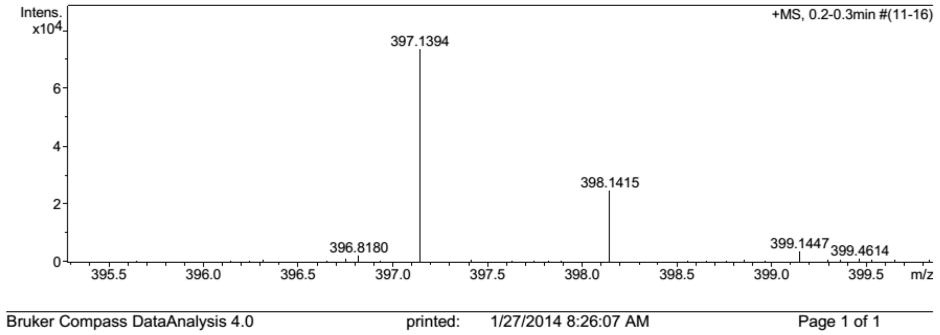

(E)-1-(4-(Benzyloxy)phenyl)-3-(2,6-dimethoxyphenyl)prop-2-en-1-one (**b13**)

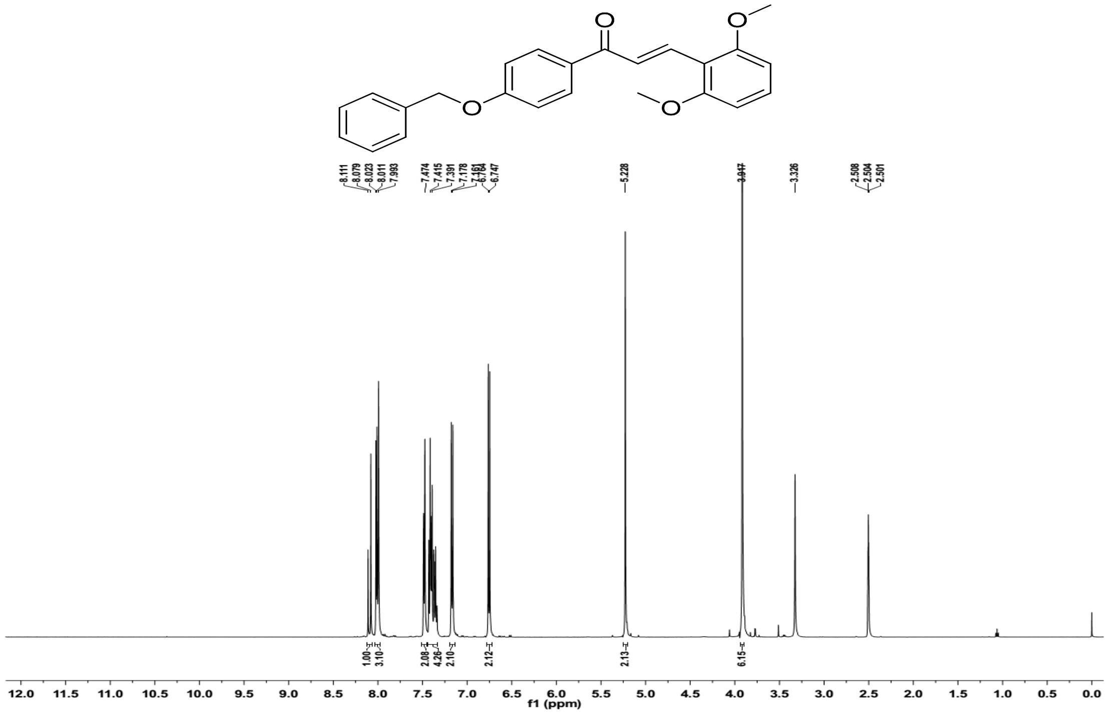

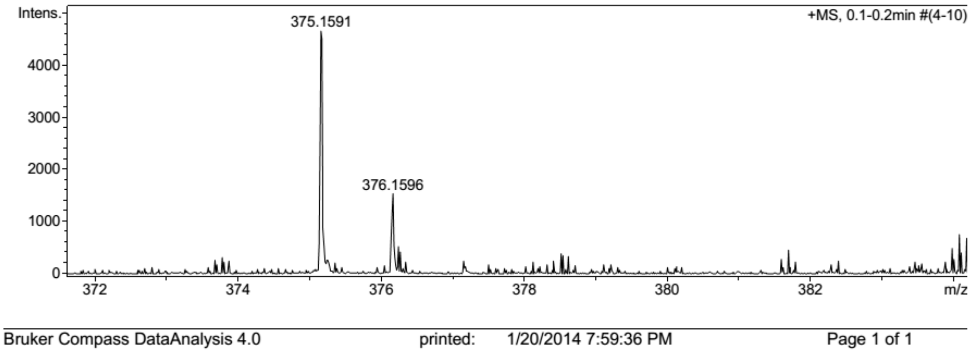

(E)-1-(2-(Benzyloxy)-4-methoxyphenyl)-3-(2,6-dimethoxyphenyl)prop-2-en-1-one (**b14**)

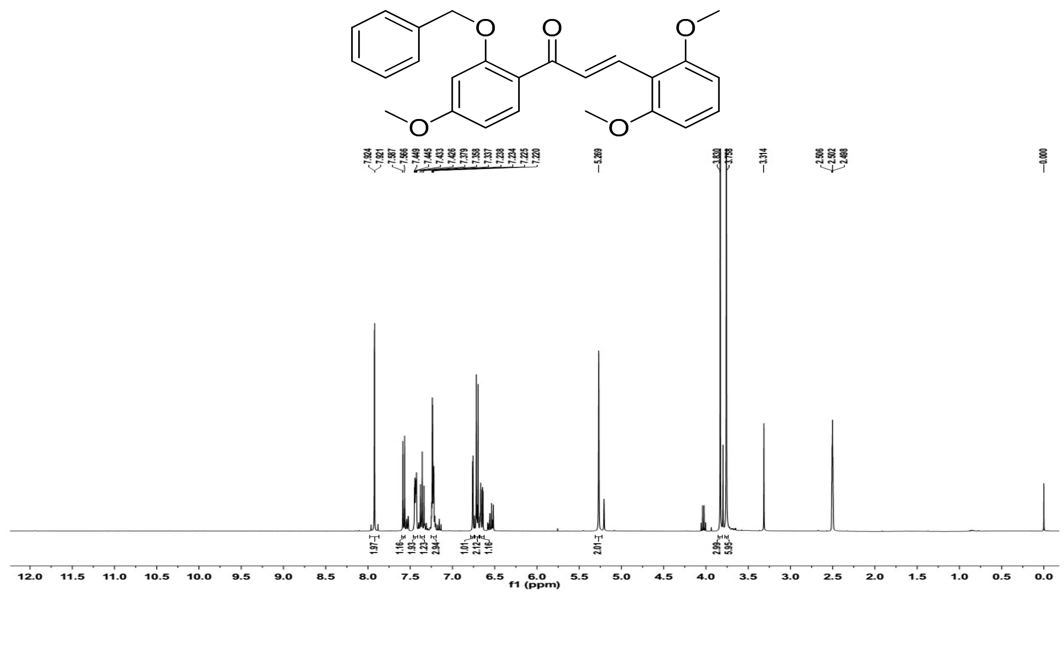

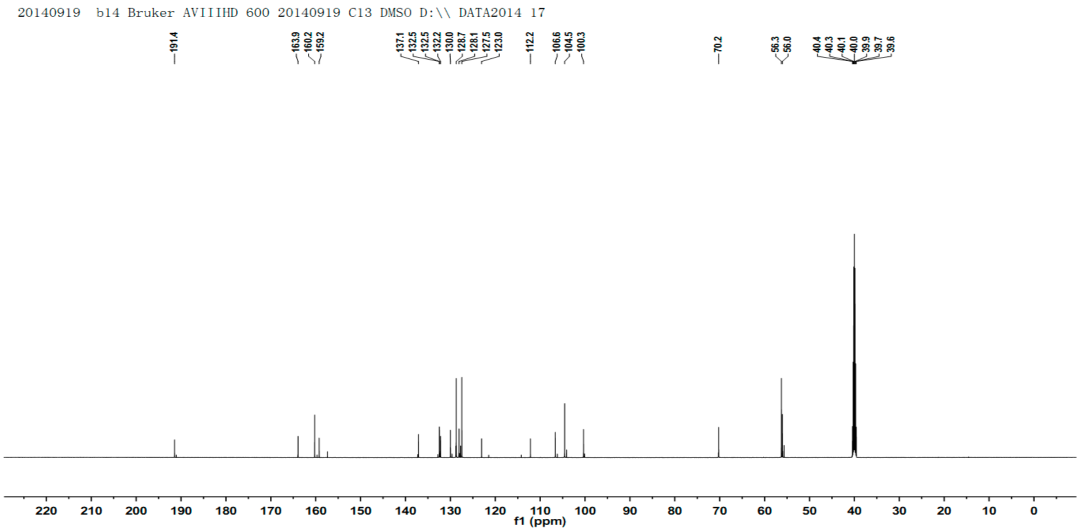

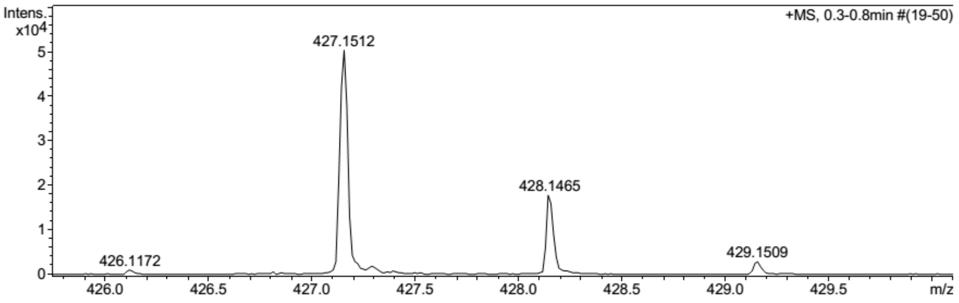

Bruker Compass DataAnalysis 4.0 printed: 1/27/2014 8:22:25 AM Page 1 of 1

(E)-1-(4-(Benzyloxy)-3-methoxyphenyl)-3-(2,6-dimethoxyphenyl)prop-2-en-1-one (**b15**)

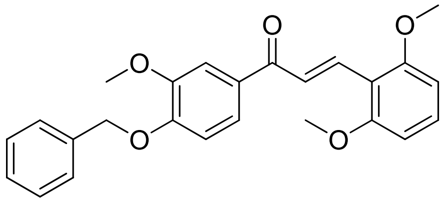

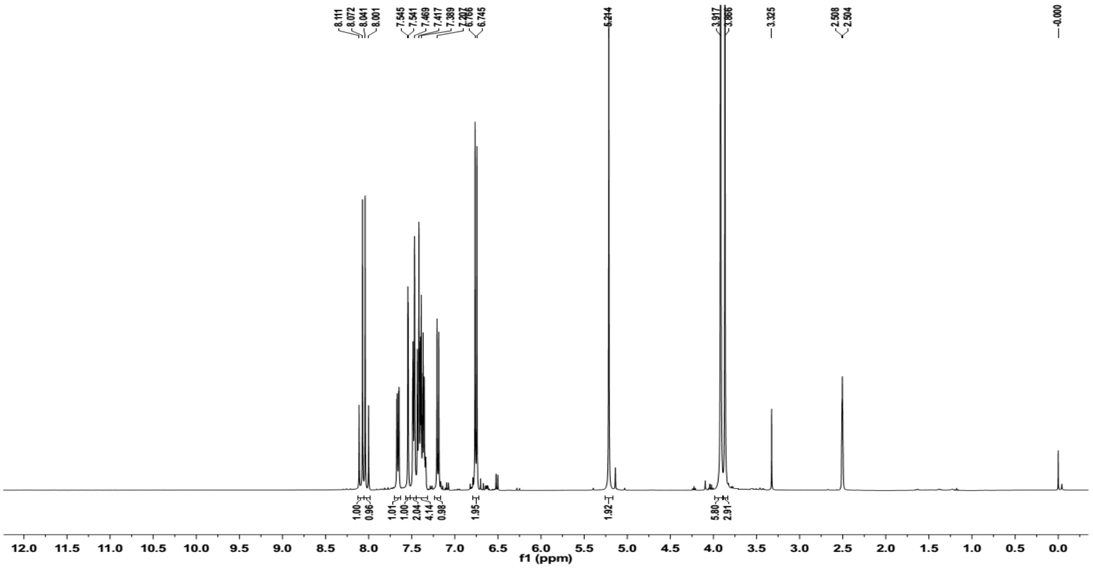

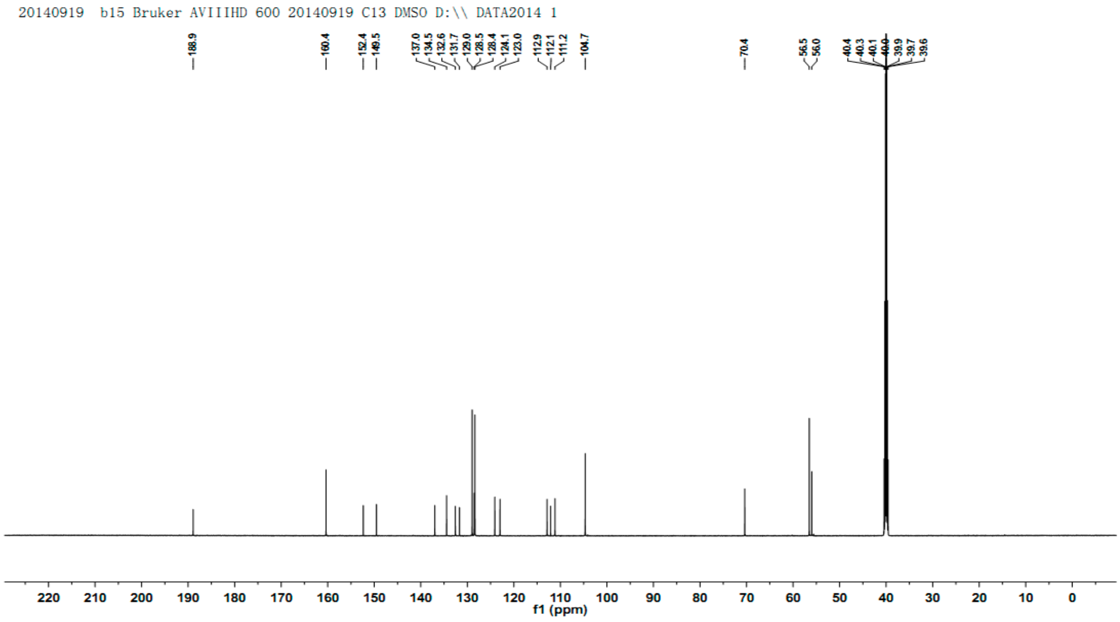

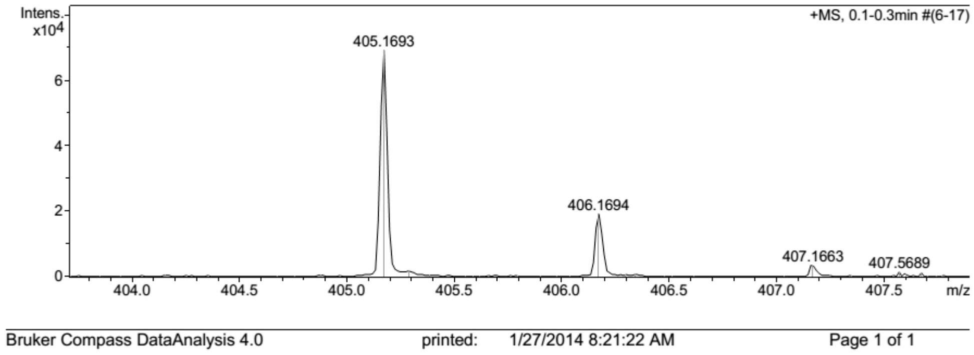

(E)-1-(4-Ethoxy-3-methoxyphenyl)-3-(2,6-dimethoxyphenyl)prop-2-en-1-one (**b16**)

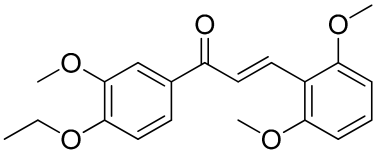

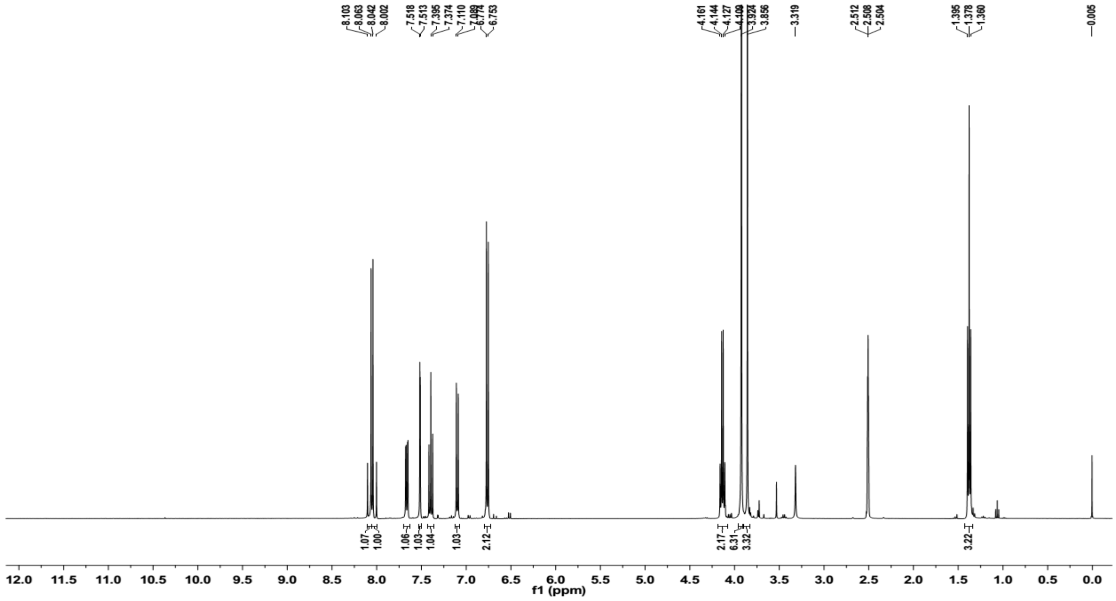

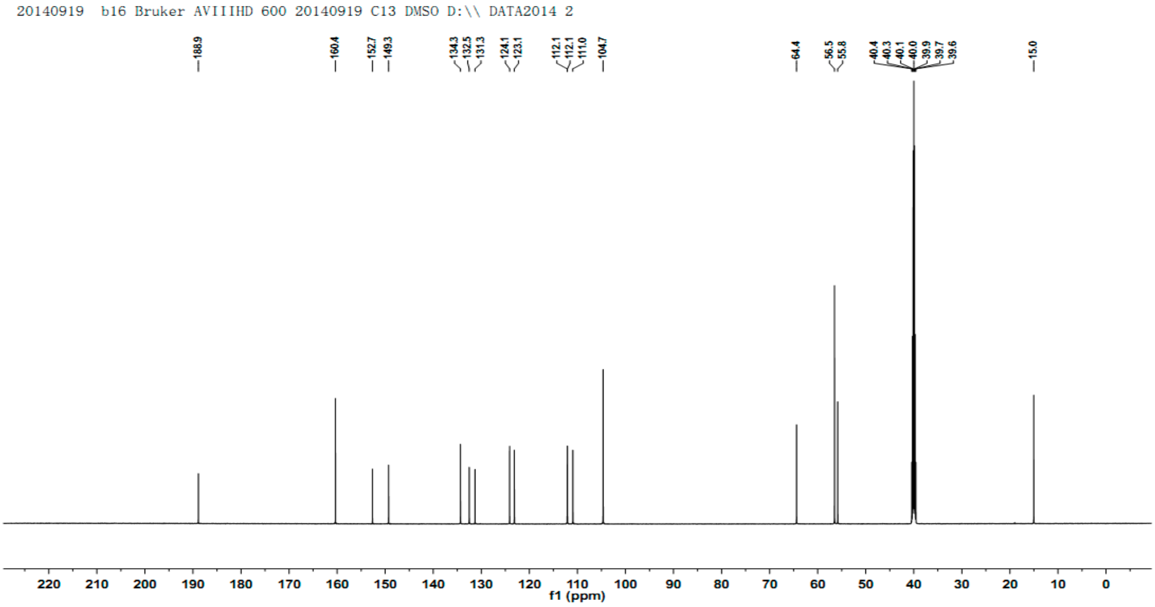

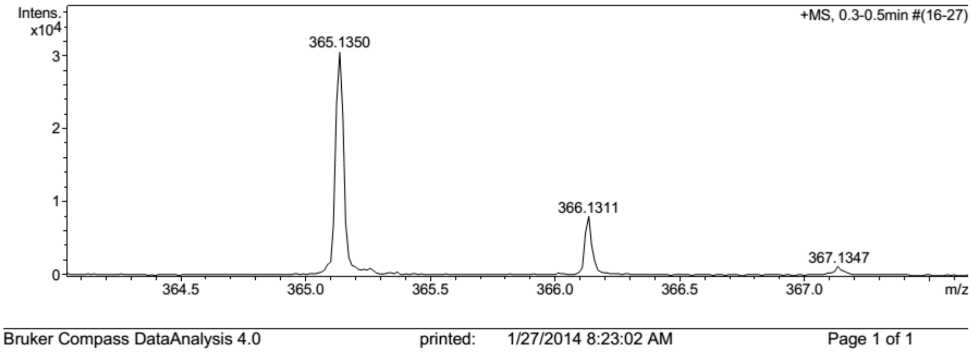

(E)-1-(2-Ethoxy-4-methoxyphenyl)-3-(2,6-dimethoxyphenyl)prop-2-en-1-one (**b17**)

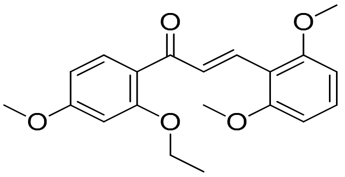

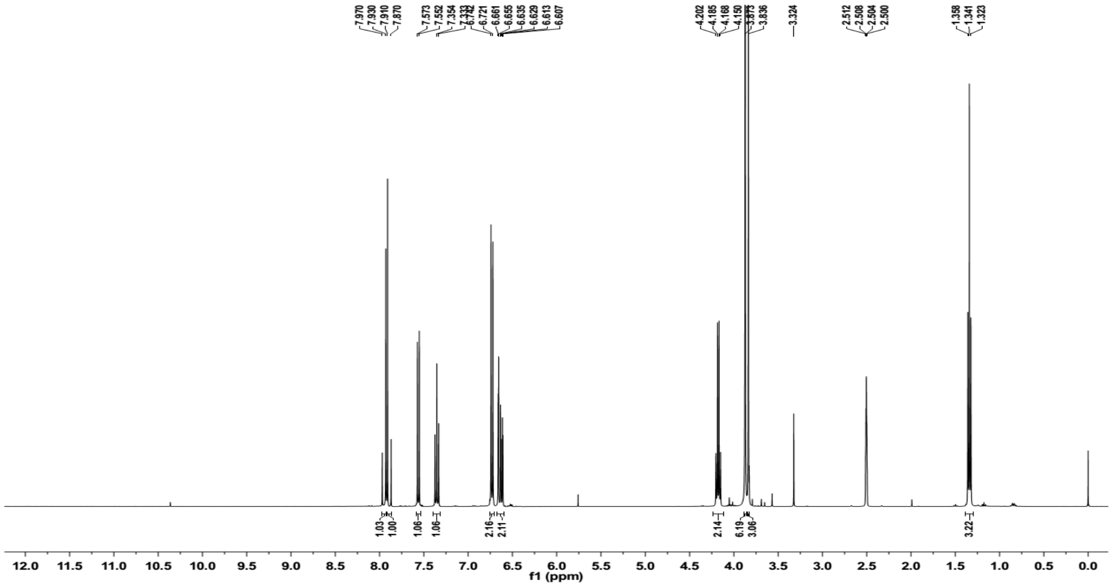

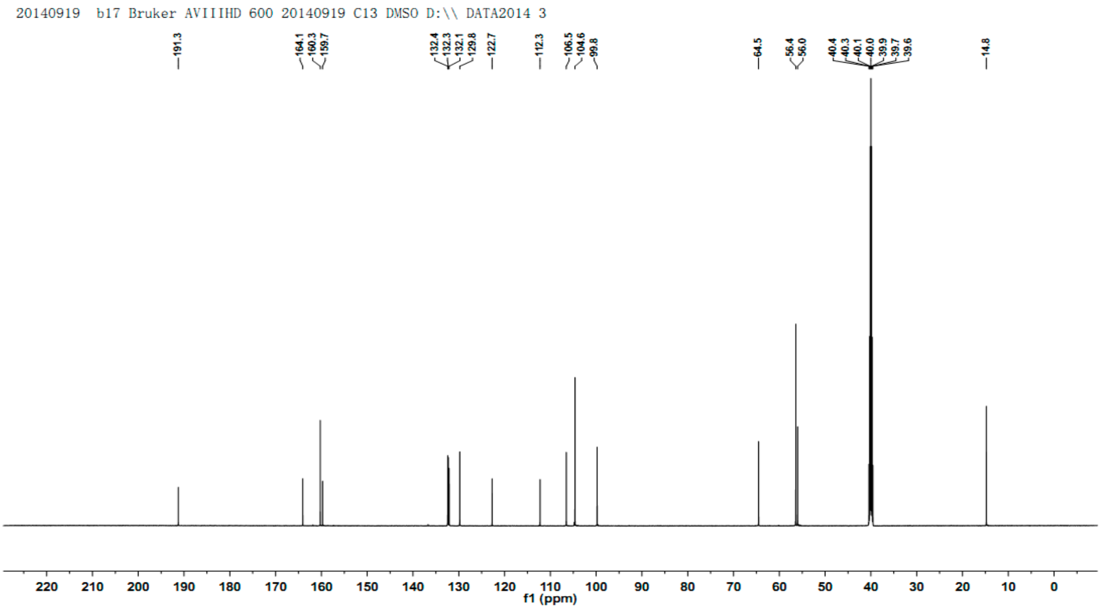

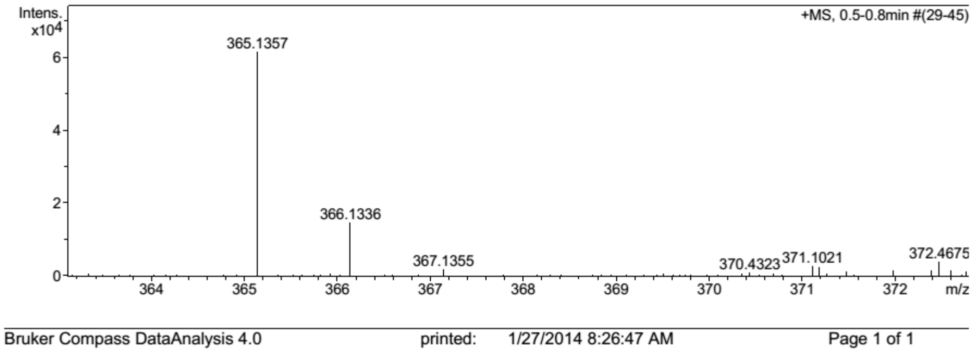

(E)-1-(2,5-Dimethoxyphenyl)-3-(2,6-dimethoxyphenyl)prop-2-en-1-one (**b18**)

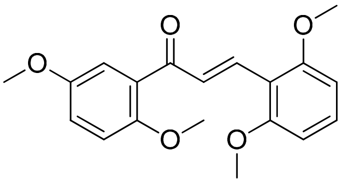

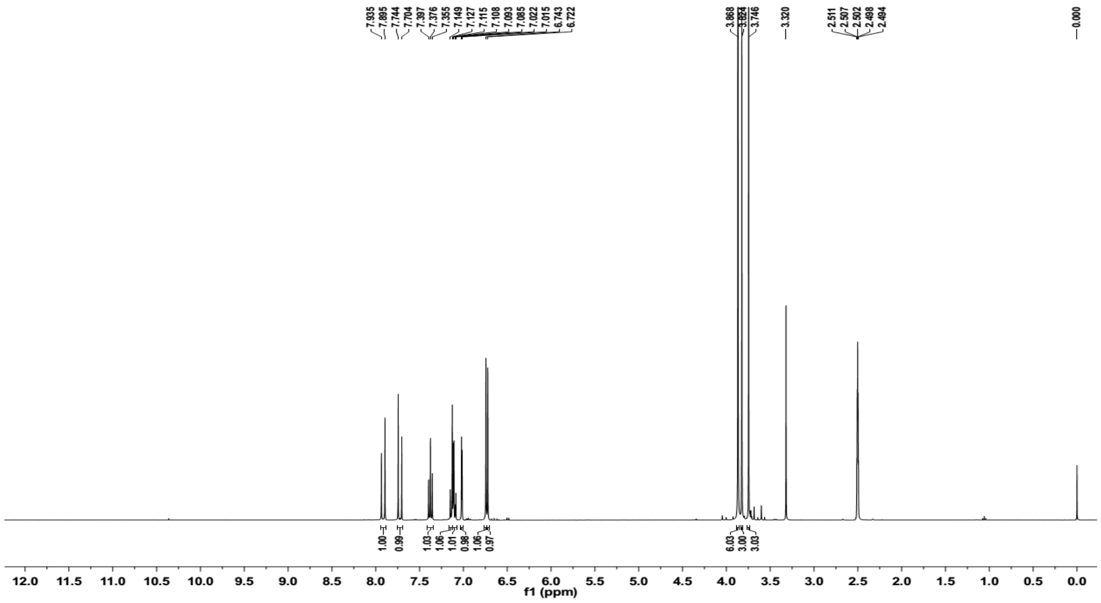

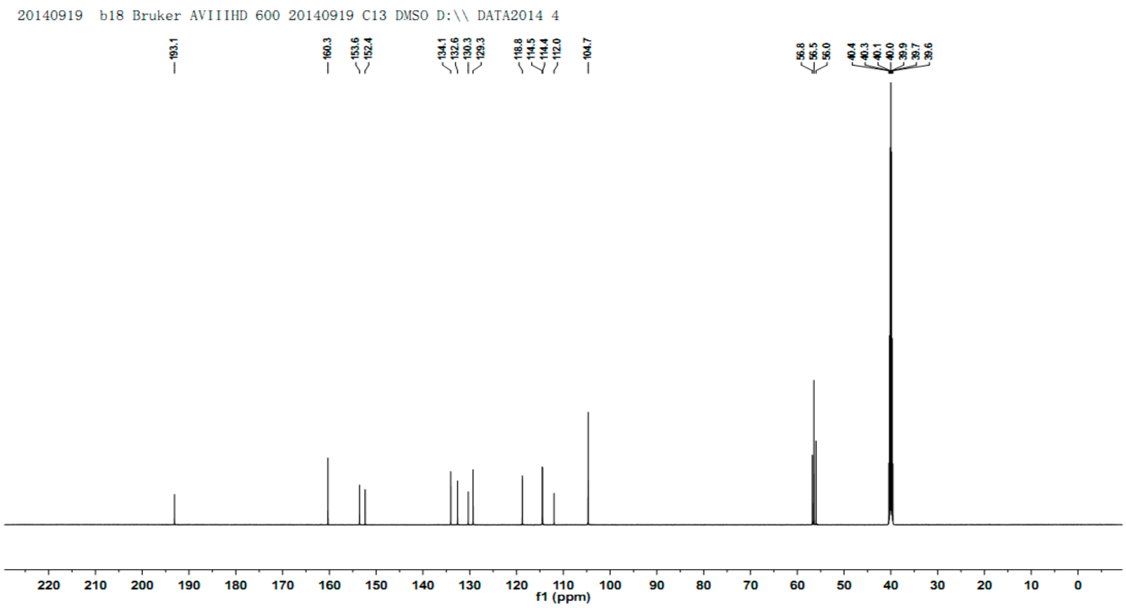

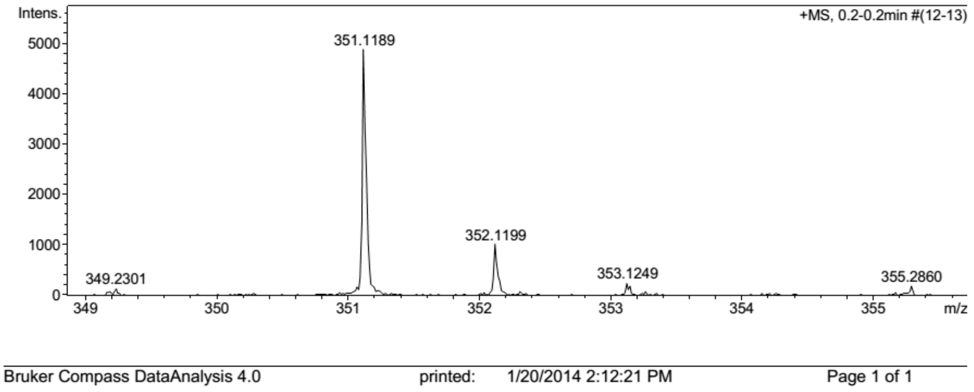

(E)-1-(3,4-Dimethoxyphenyl)-3-(2,6-dimethoxyphenyl)prop-2-en-1-one (**b19**)

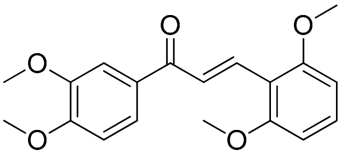

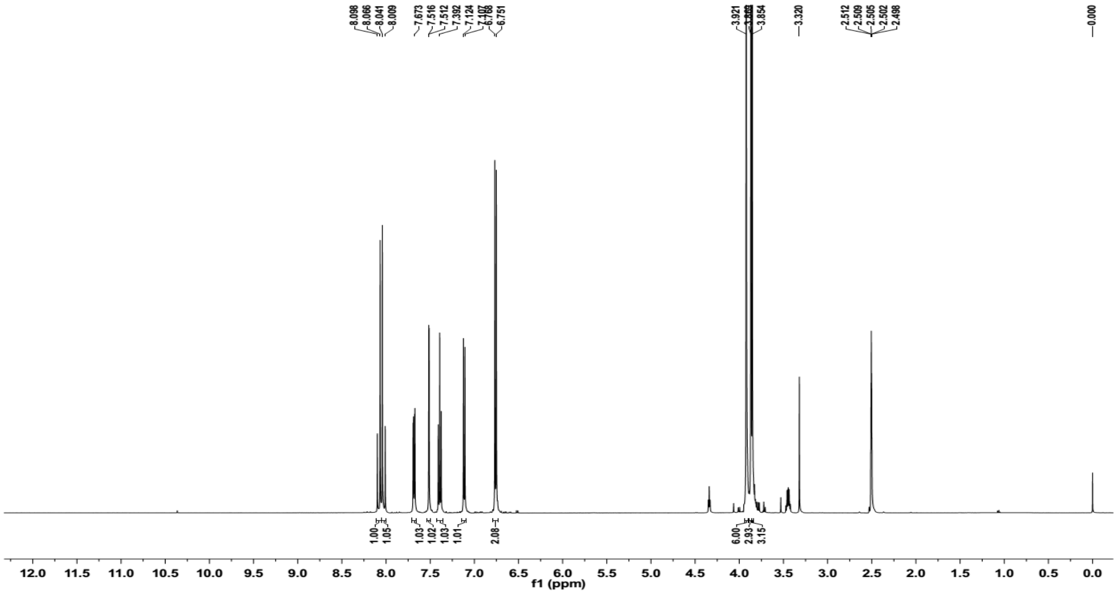

(E)-1-(Benzo[d][1,3]dioxol-5-yl)-3-(2,6-dimethoxyphenyl)prop-2-en-1-one (**b20**)

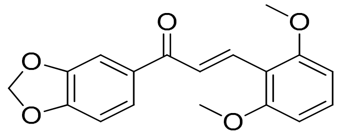

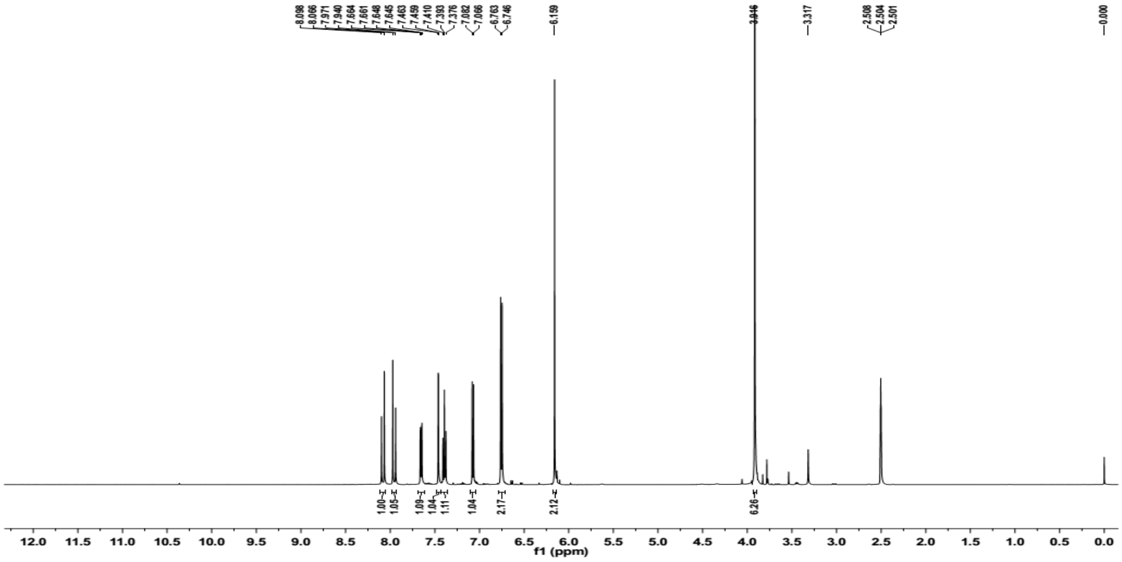

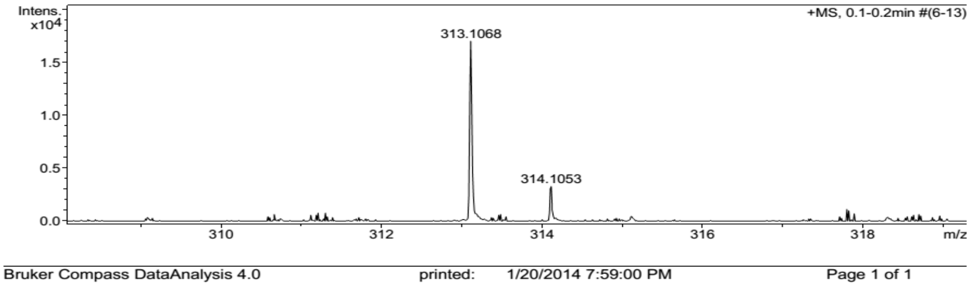

(E)-1-(2,3-Dihydrobenzofuran-5-yl)-3-(2,6-dimethoxyphenyl)prop-2-en-1-one (**b21**)

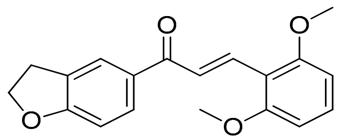

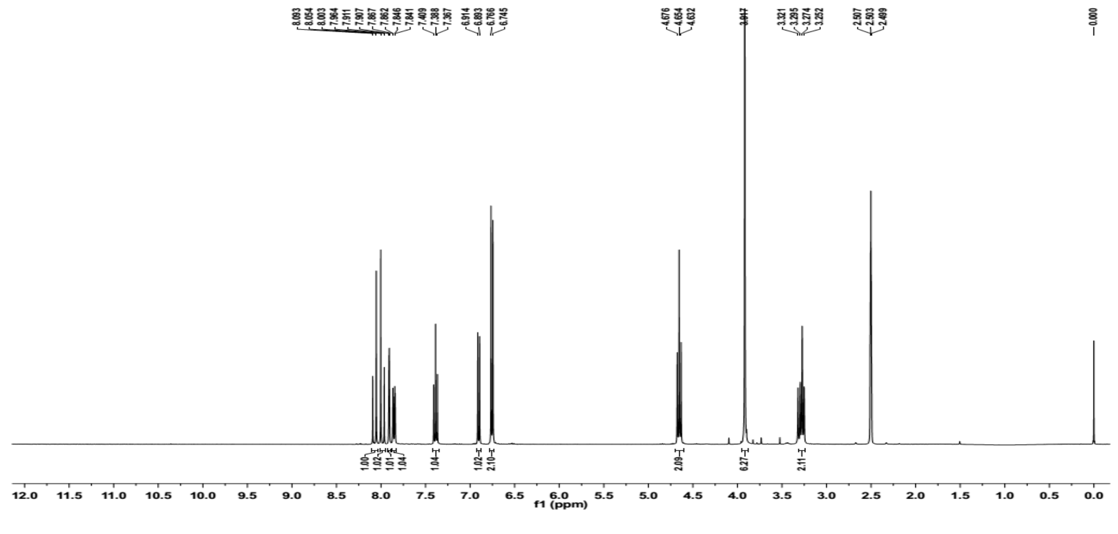

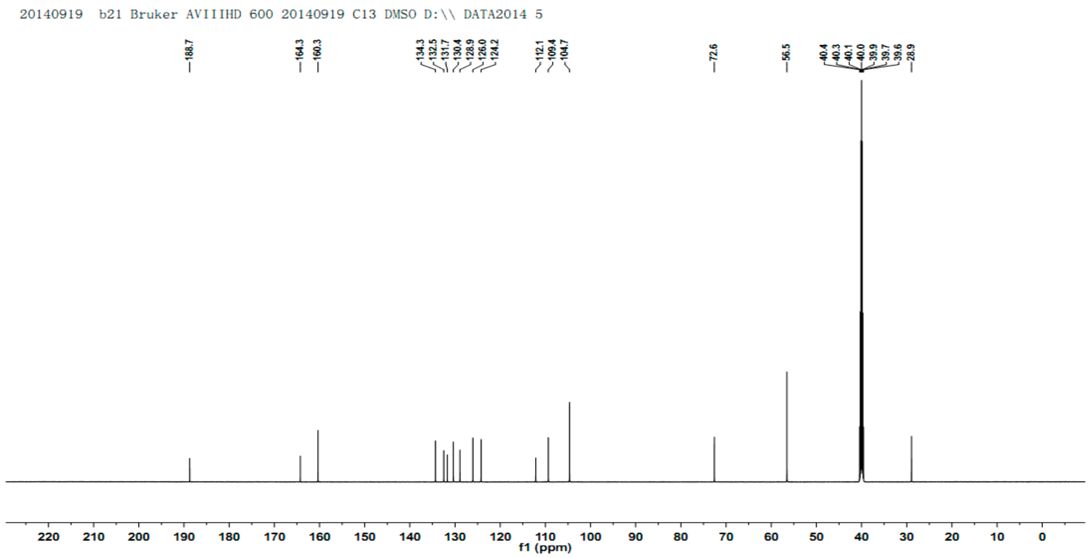

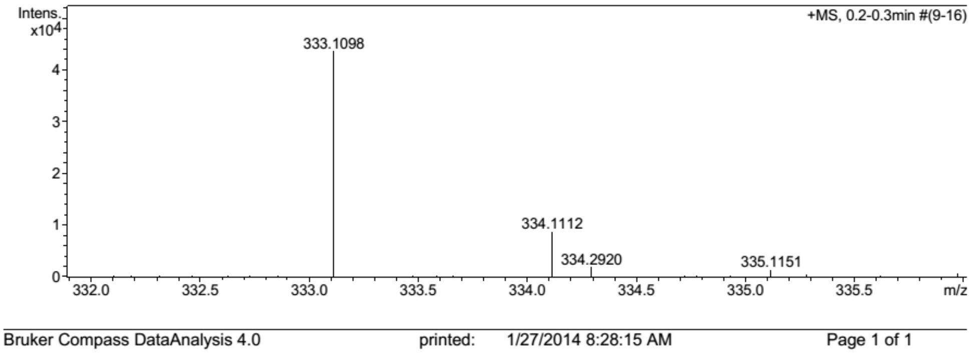

(E)-1-([1,1'-Biphenyl]-4-yl)-3-(2,6-dimethoxyphenyl)prop-2-en-1-one (**b22**)

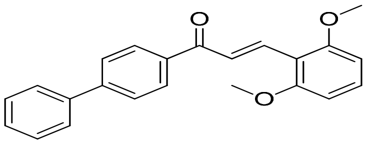

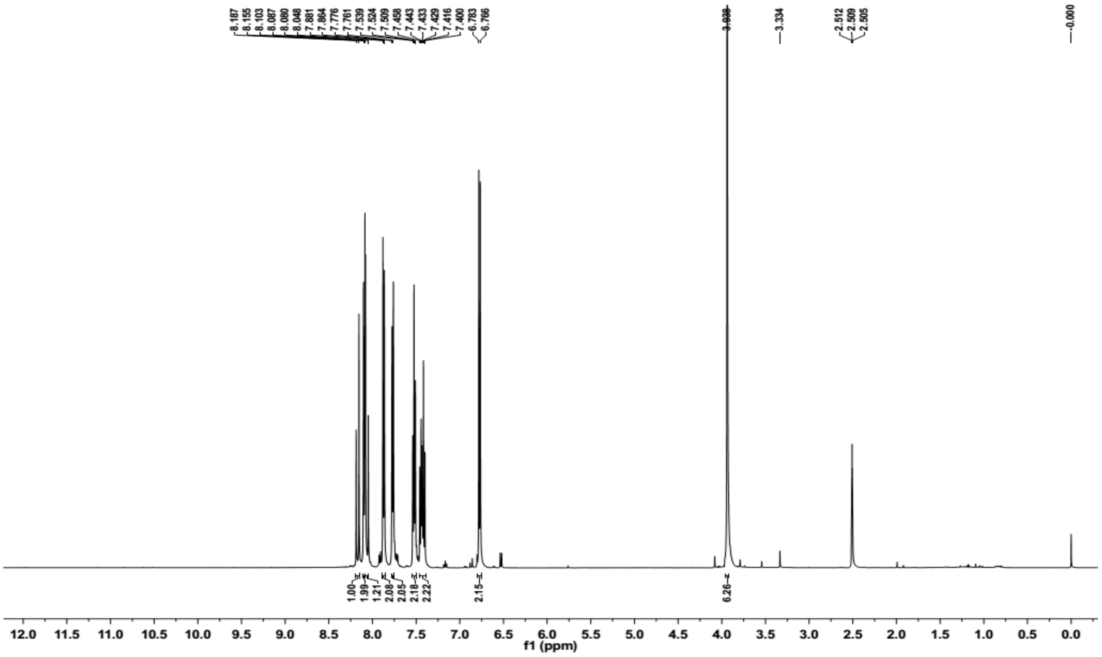

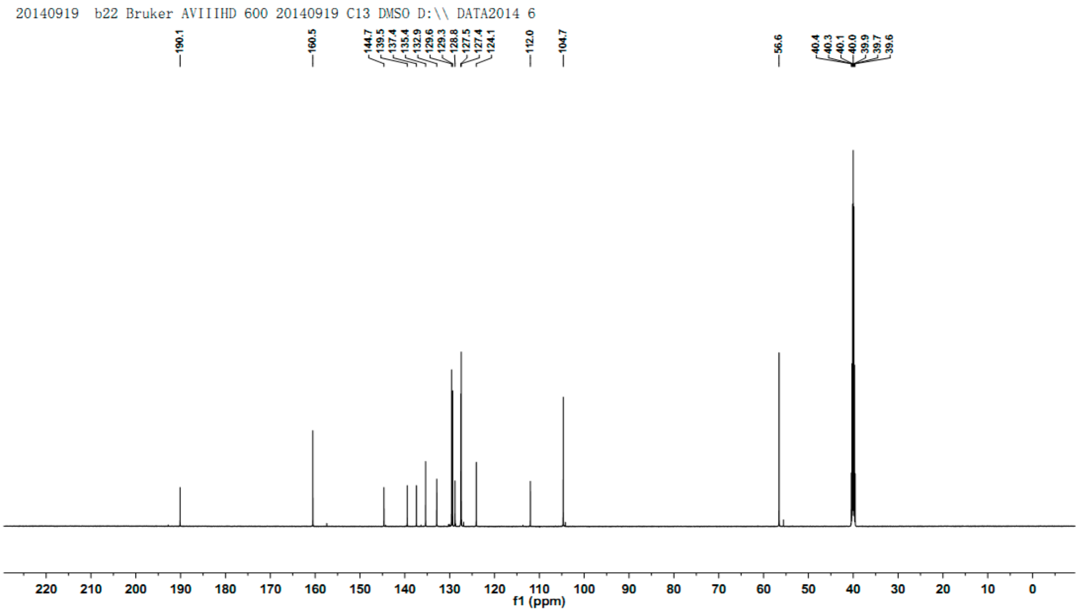

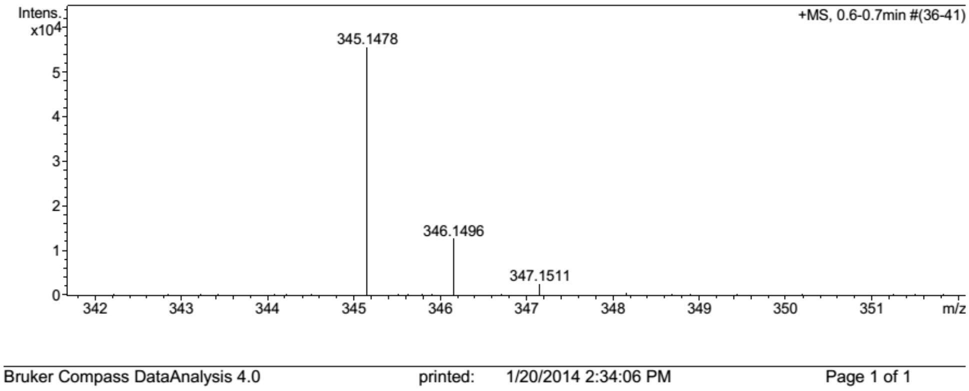

(E)-1-(3-Aminophenyl)-3-(2,6-dimethoxyphenyl)prop-2-en-1-one (**b23**)

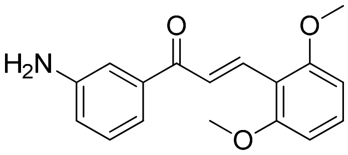

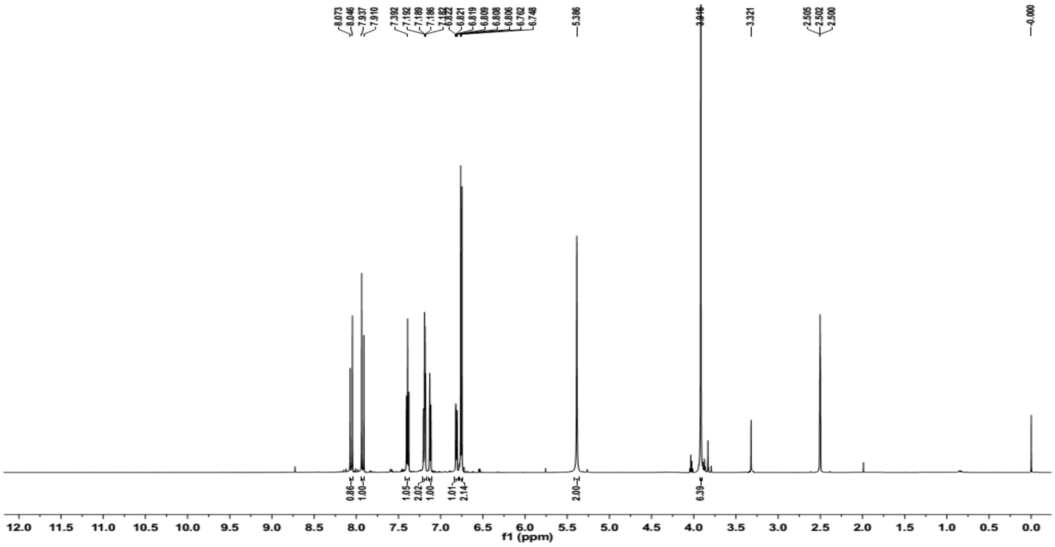

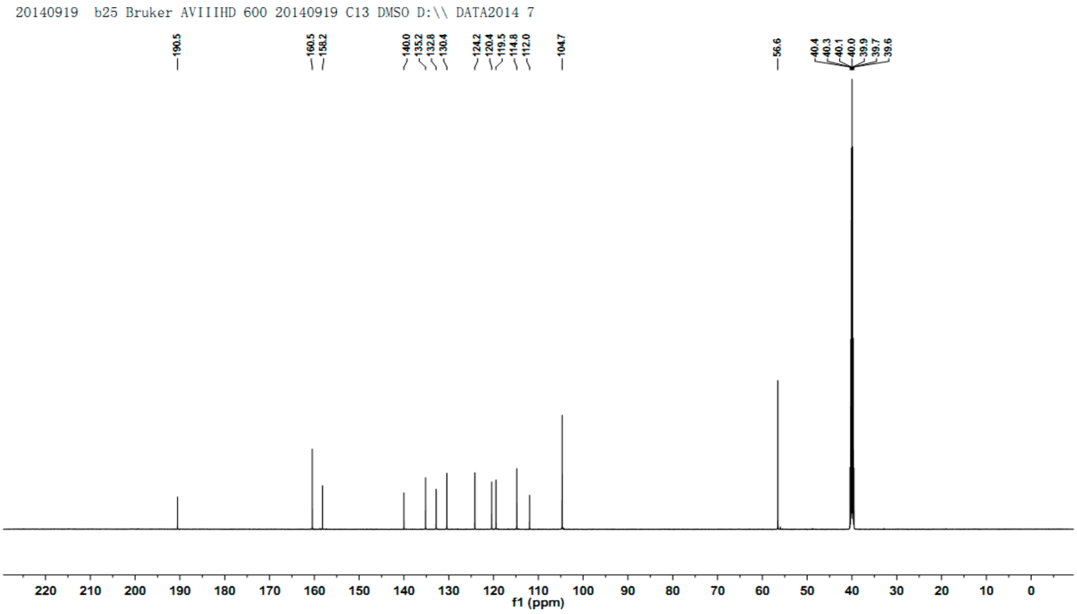

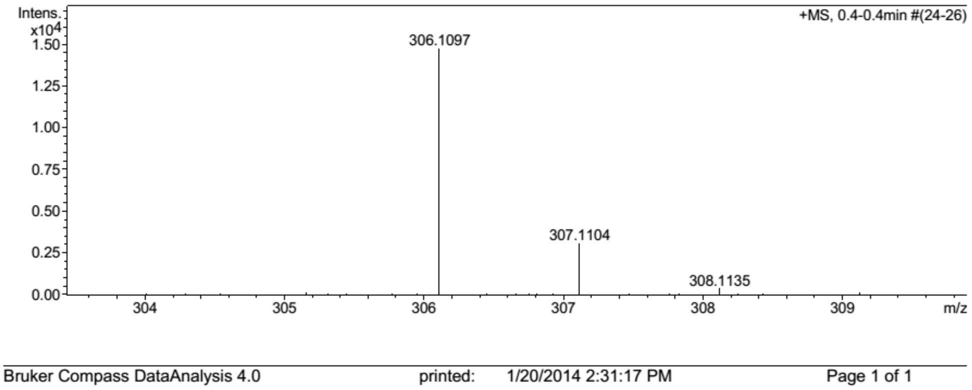

(E)-1-(2-Hydroxyphenyl)-3-(2,6-dimethoxyphenyl)prop-2-en-1-one (**b24**)

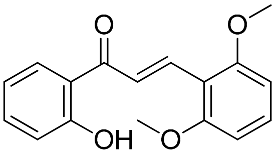

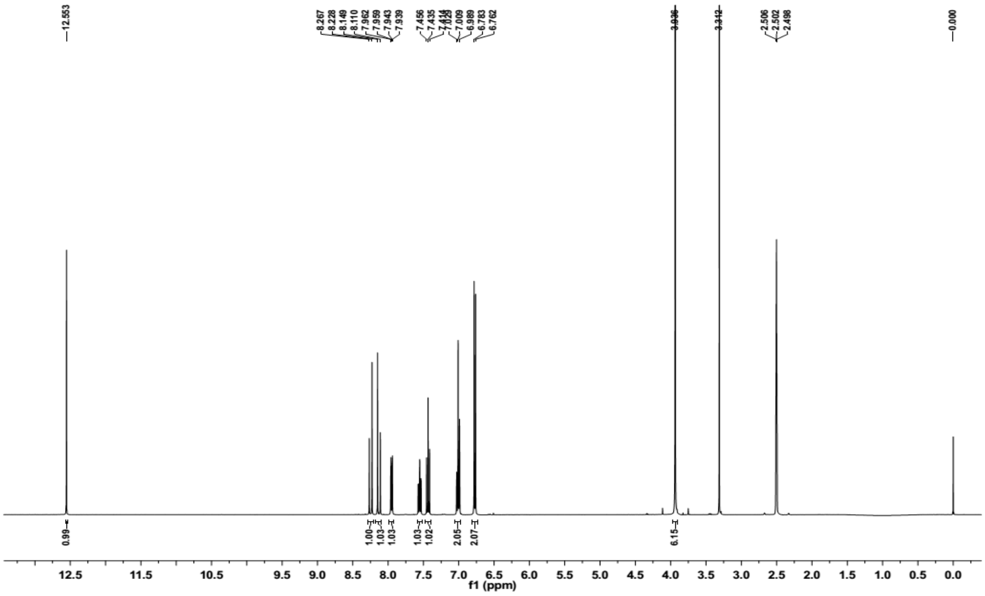

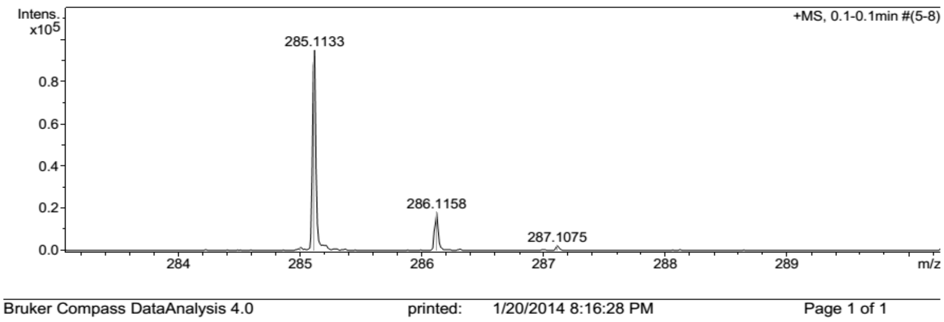

(E)-1-(3-Hydroxyphenyl)-3-(2,6-dimethoxyphenyl)prop-2-en-1-one (**b25**)

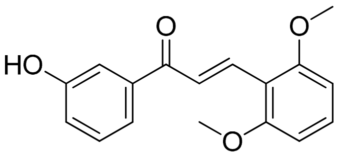

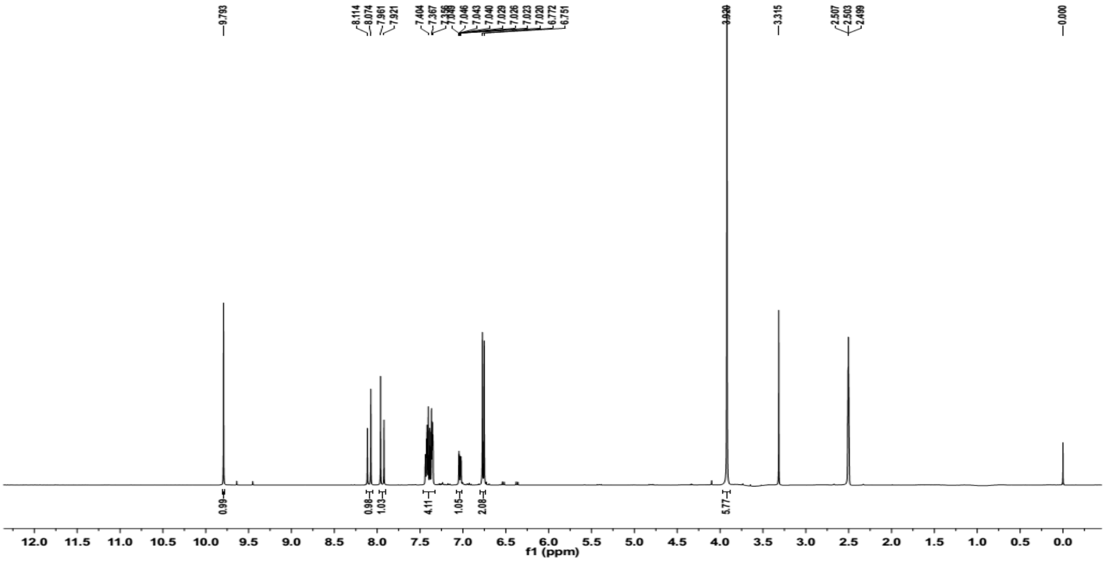

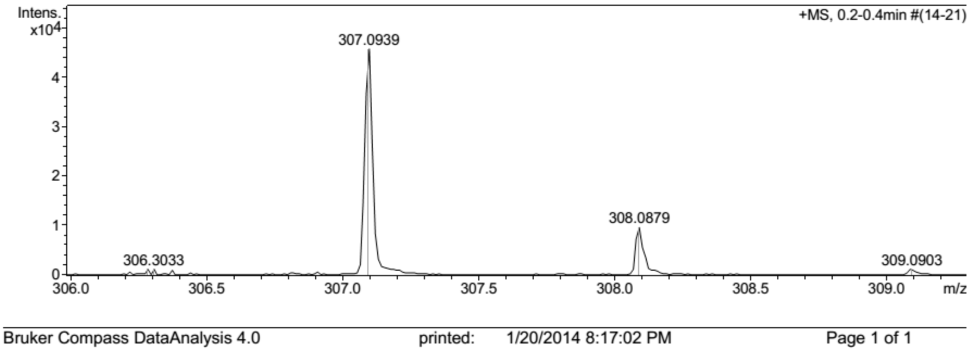

(E)-1-(4-Hydroxyphenyl)-3-(2,6-dimethoxyphenyl)prop-2-en-1-one (**b26**)

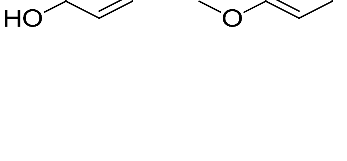

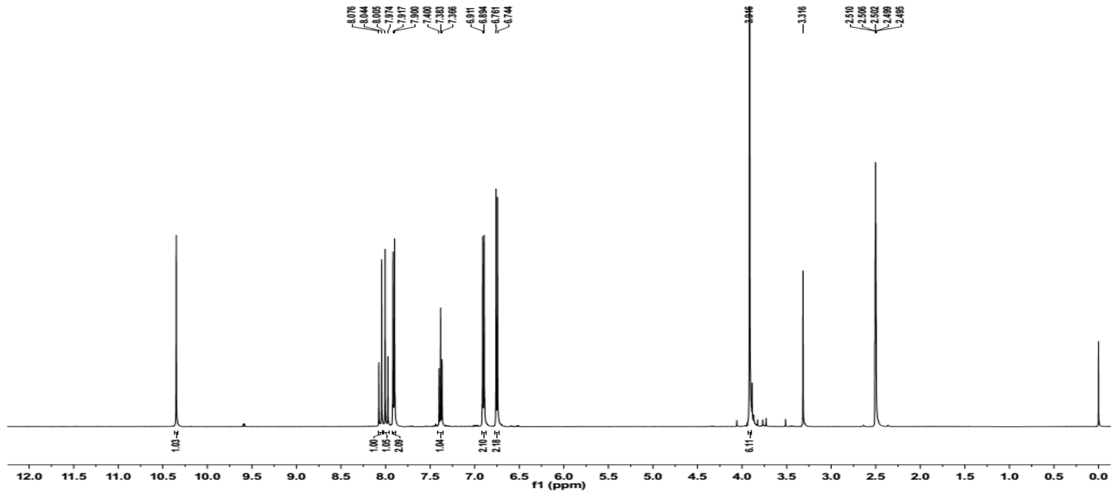

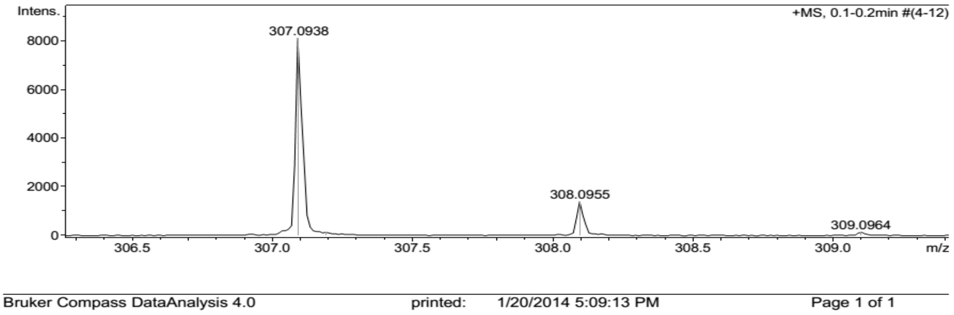

(E)-1-(2,4-Dihydroxyphenyl)-3-(2,6-dimethoxyphenyl)prop-2-en-1-one (**b27**)

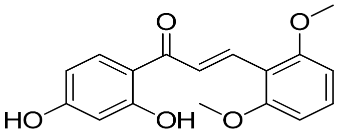

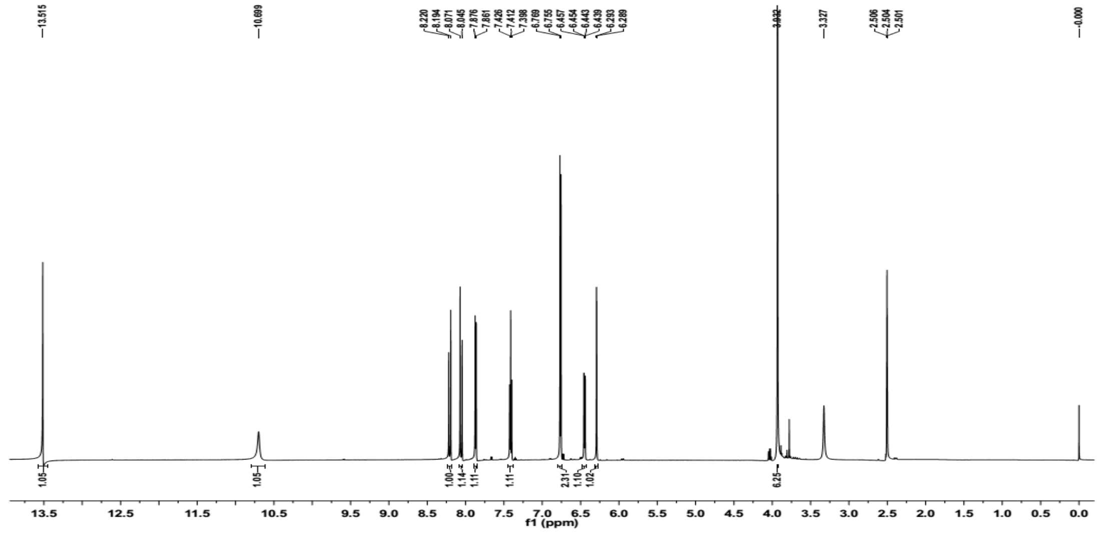

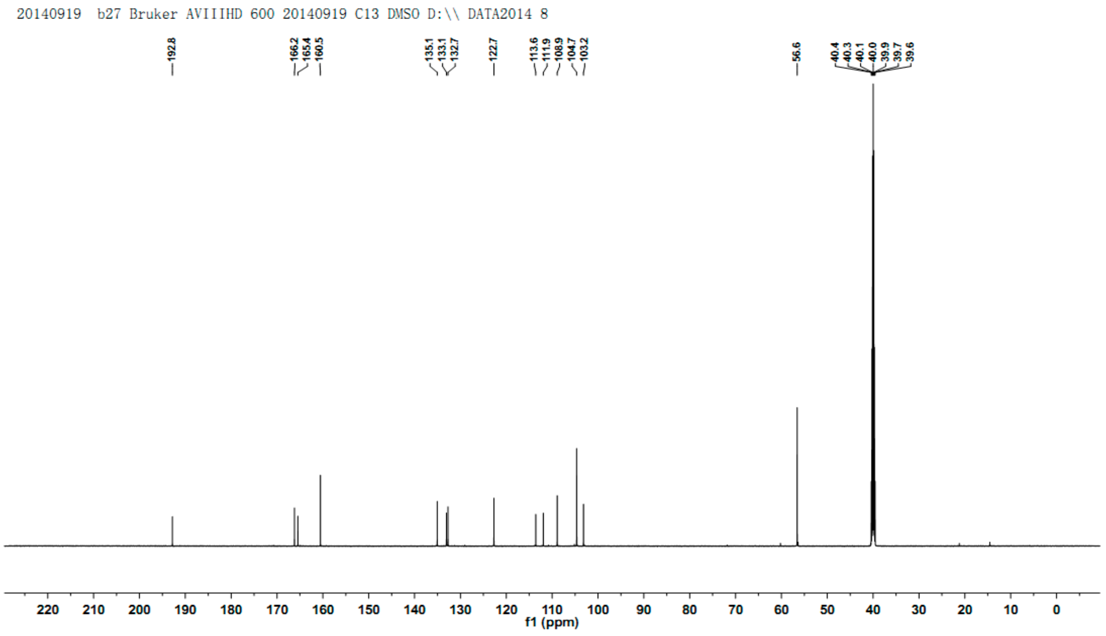

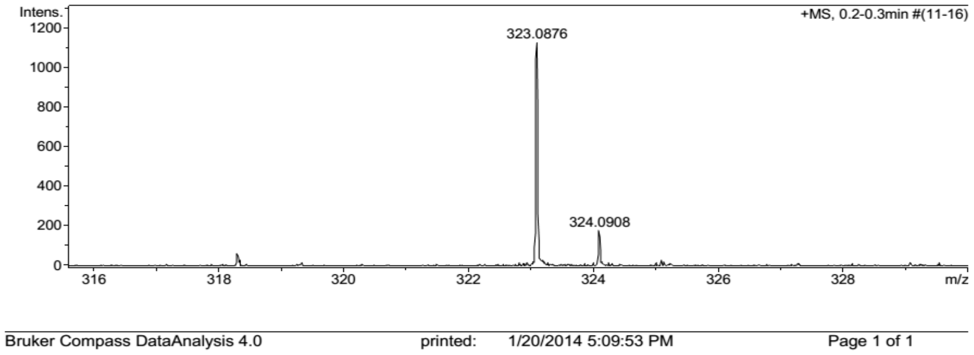

(E)-1-(2-Hydroxy-4-methoxyphenyl)-3-(2,6-dimethoxyphenyl)prop-2-en-1-one (**b28**)

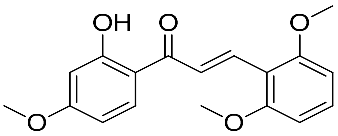

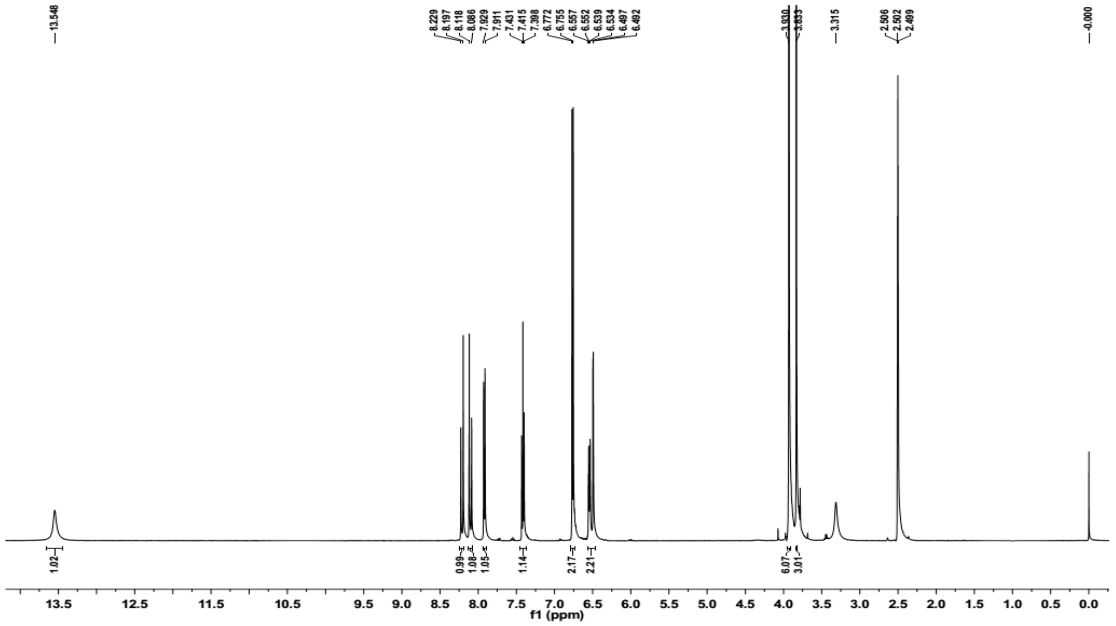

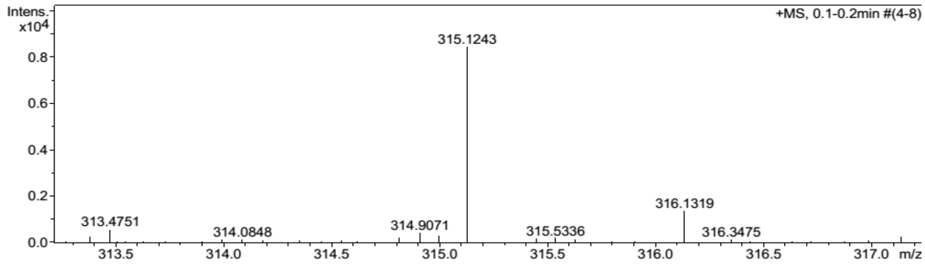

Bruker Compass DataAnalysis 4.0 printed: 1/20/2014 8:00:36 PM Page 1 of 1

(E)-1-(4-Hydroxy-3-methoxyphenyl)-3-(2,6-dimethoxyphenyl)prop-2-en-1-one (**b29**)

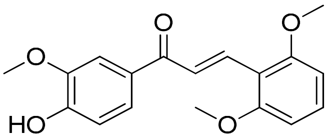

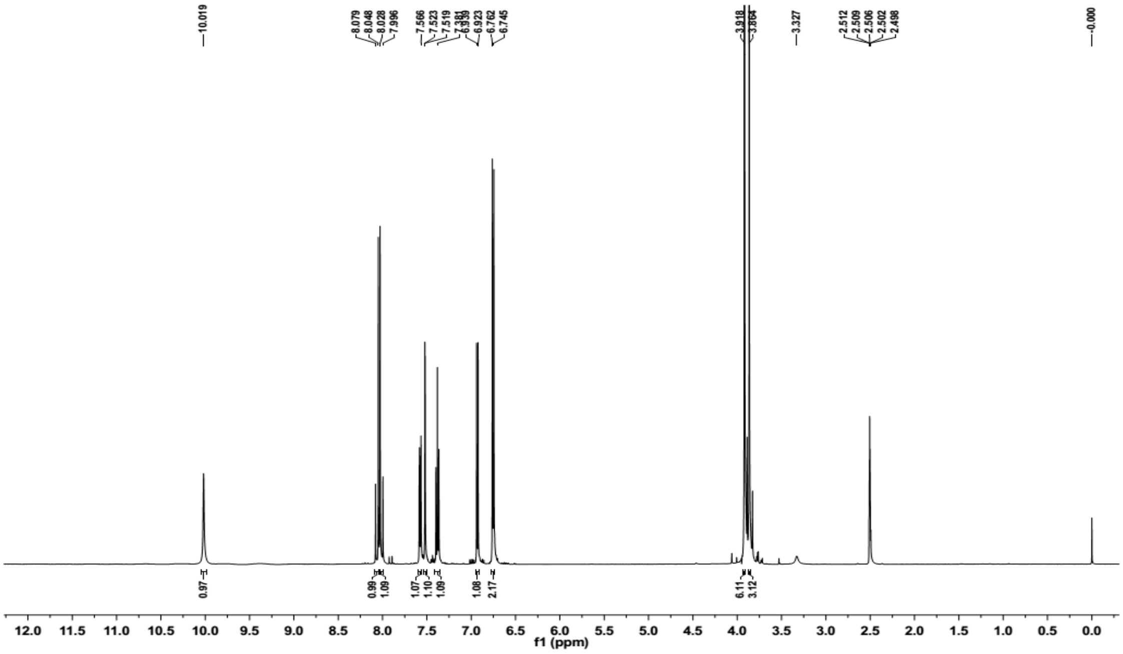

20140919 b29 Bruker AVIIIHD 600 20140919 C13 DMSO D:\\ DATA2014 9

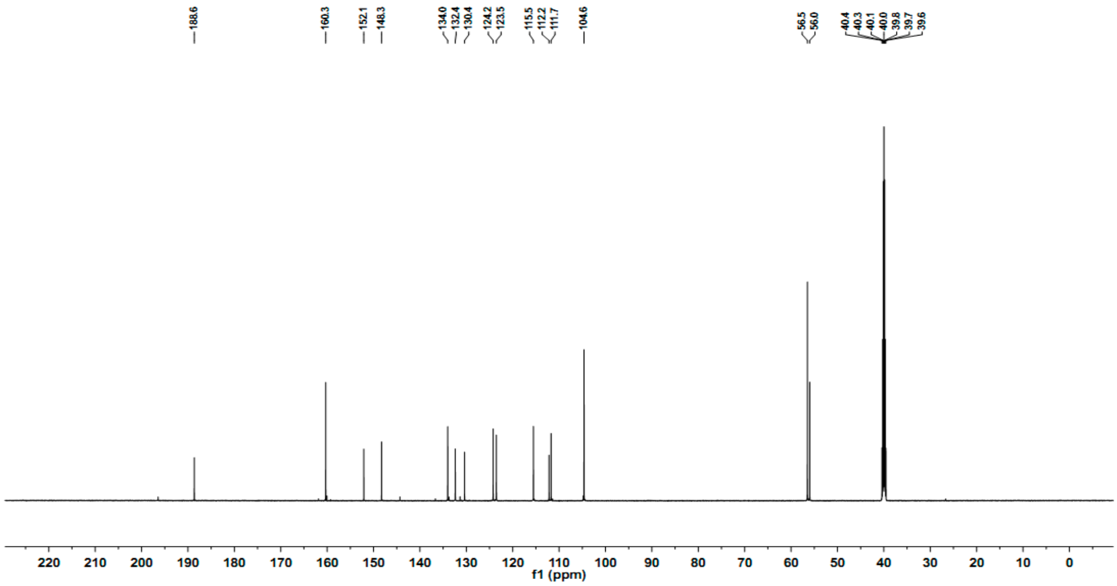

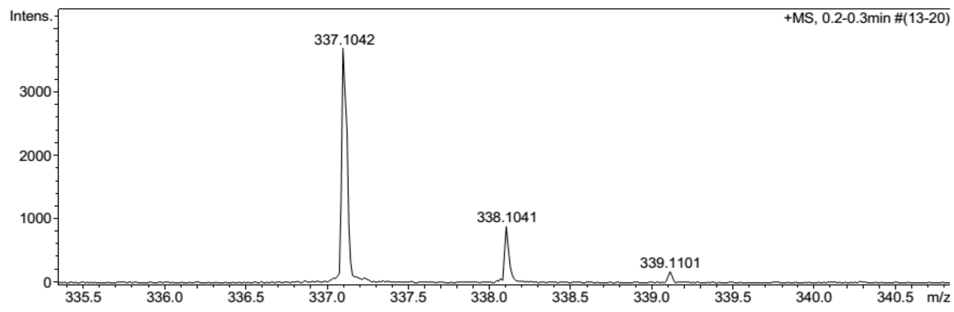

Bruker Compass DataAnalysis 4.0 printed: 1/20/2014 5:10:47 PM Page 1 of 1

(E)-1-(4-Cyanophenyl)-3-(2,6-dimethoxyphenyl)prop-2-en-1-one (**b30**)

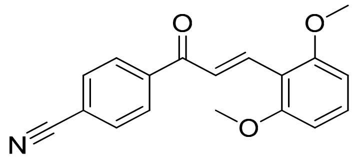

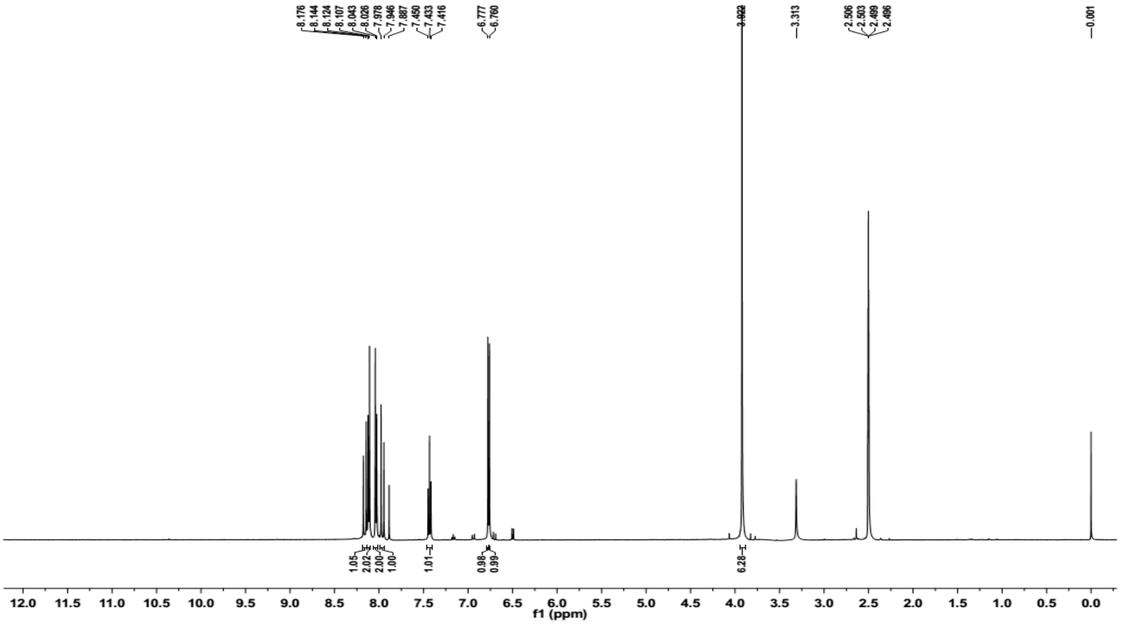

20140919 b30 Bruker AVIIIHD 600 20140919 C13 DMSO D:\\ DATA2014 10

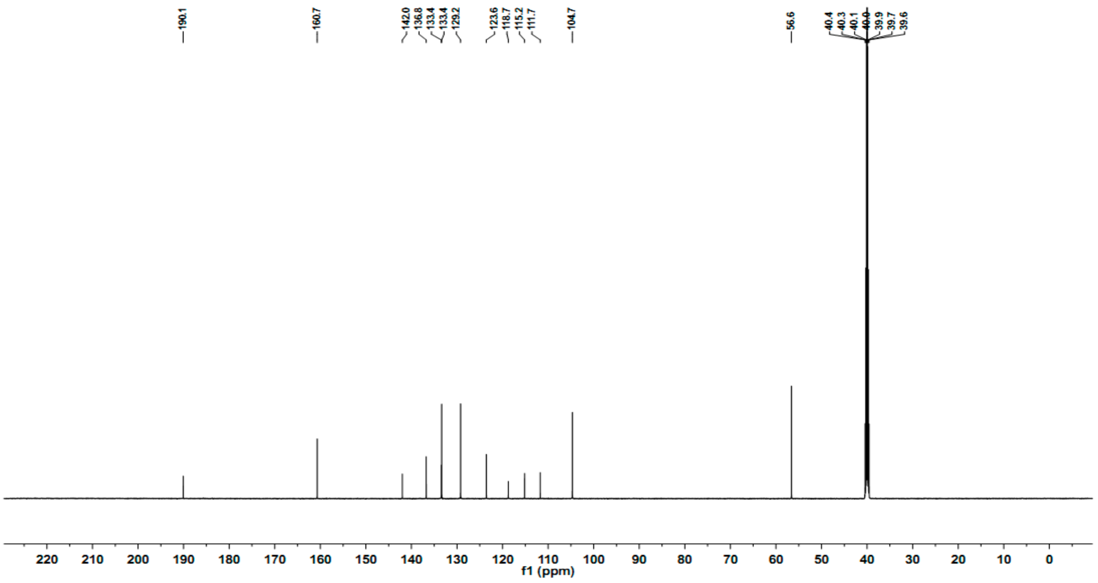

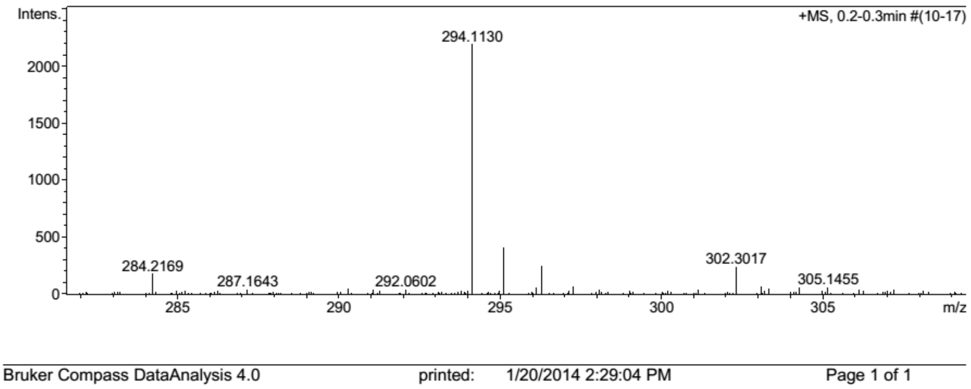

(E)-1-(2-Fluorophenyl)-3-(2,6-dimethoxyphenyl)prop-2-en-1-one (**b31**)

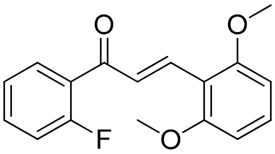

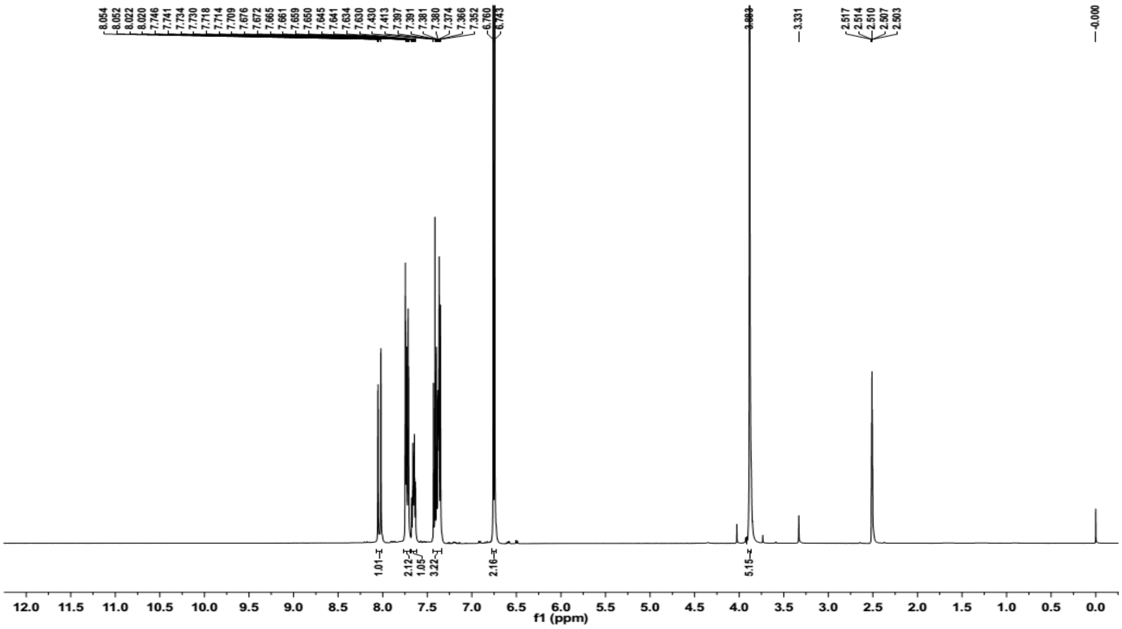

20140919 b31 Bruker AVIIIHD 600 20140919 C13 DMSO D:\\ DATA2014 11

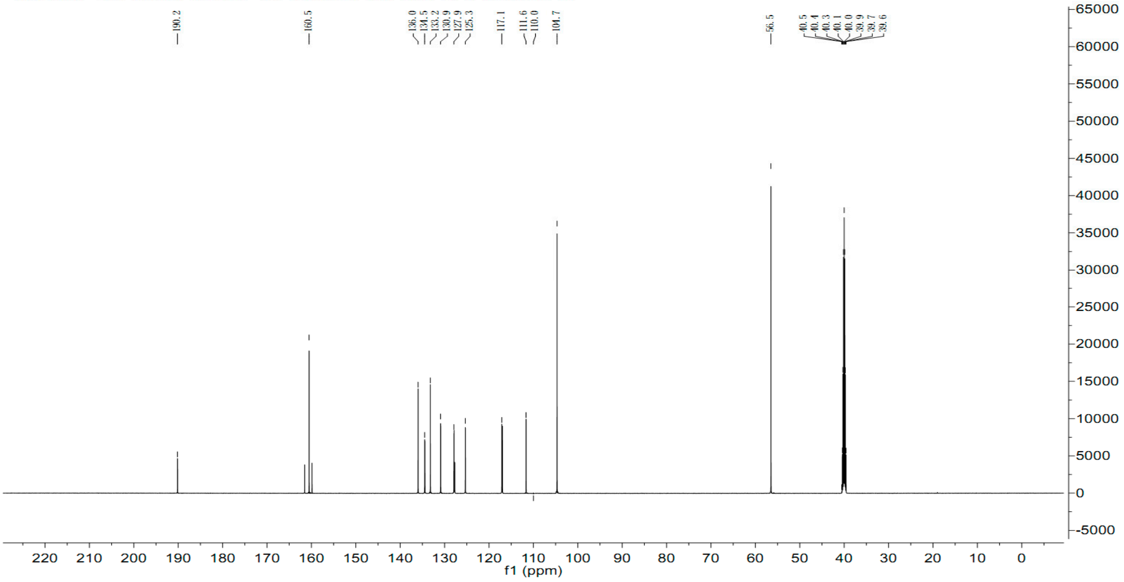

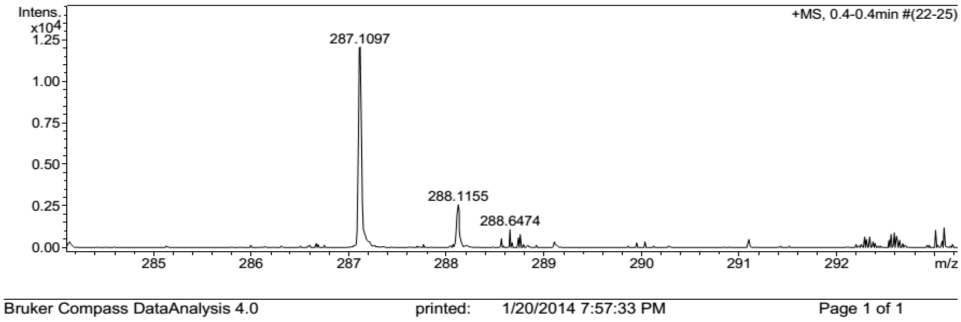

(E)-1-(3-Fluorophenyl)-3-(2,6-dimethoxyphenyl)prop-2-en-1-one (**b32**)

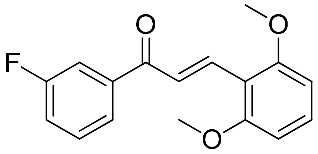

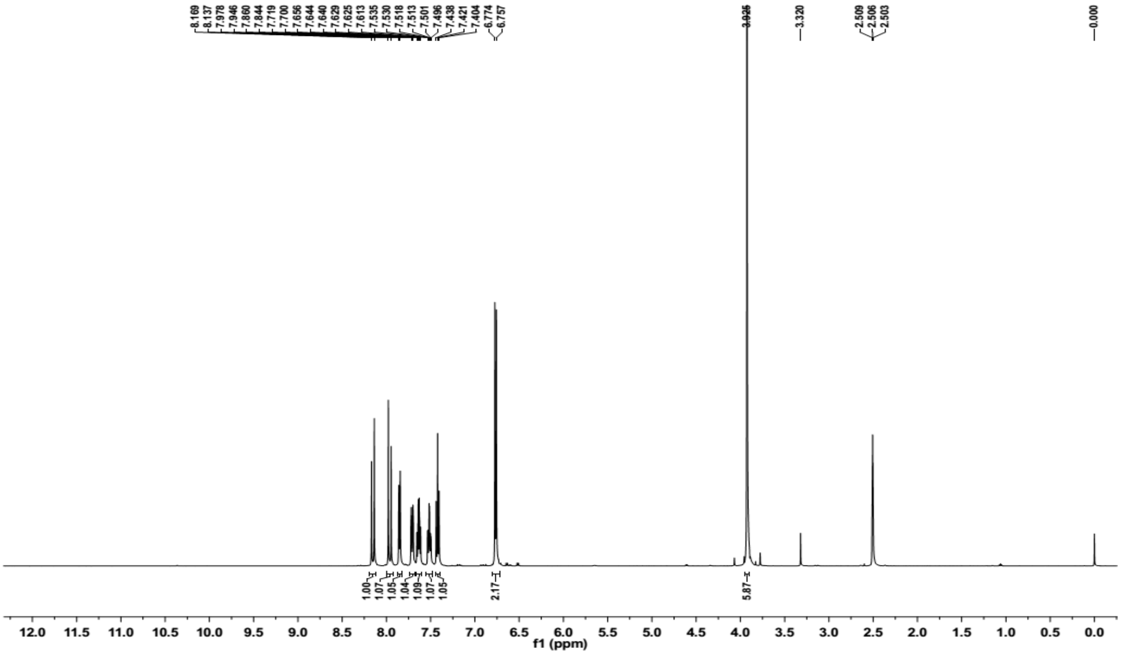

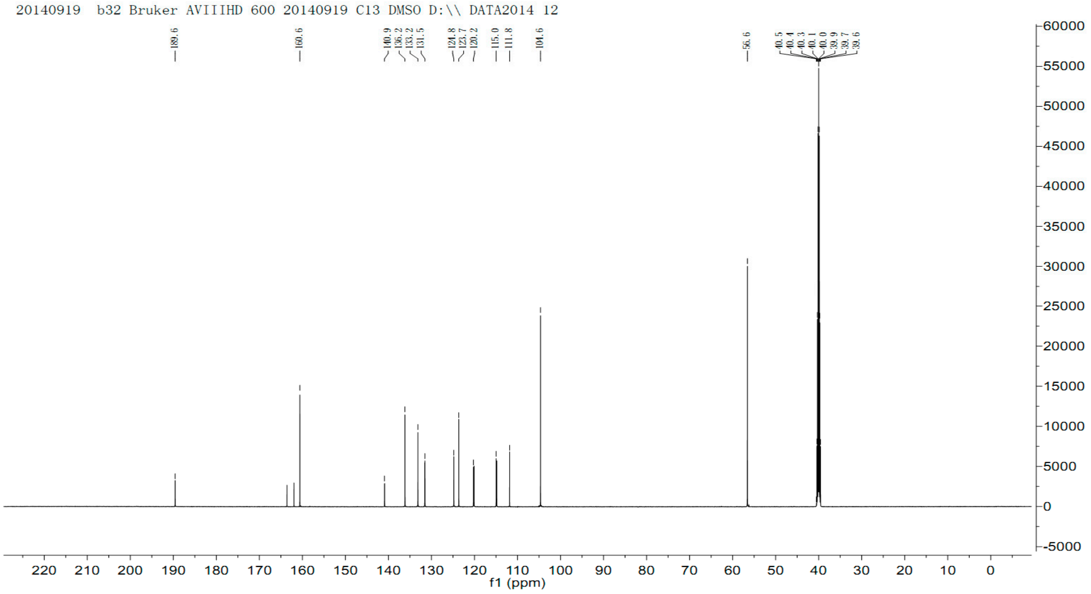

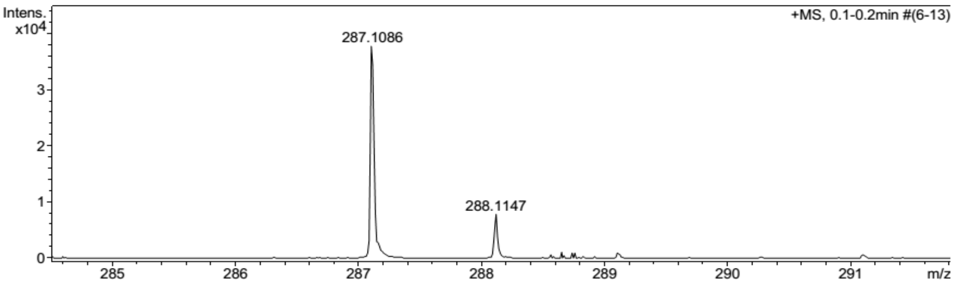

Bruker Compass DataAnalysis 4.0 printed: 1/20/2014 7:58:23 PM Page 1 of 1

(E)-1-(4-Fluorophenyl)-3-(2,6-dimethoxyphenyl)prop-2-en-1-one (**b33**)

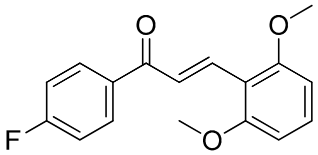

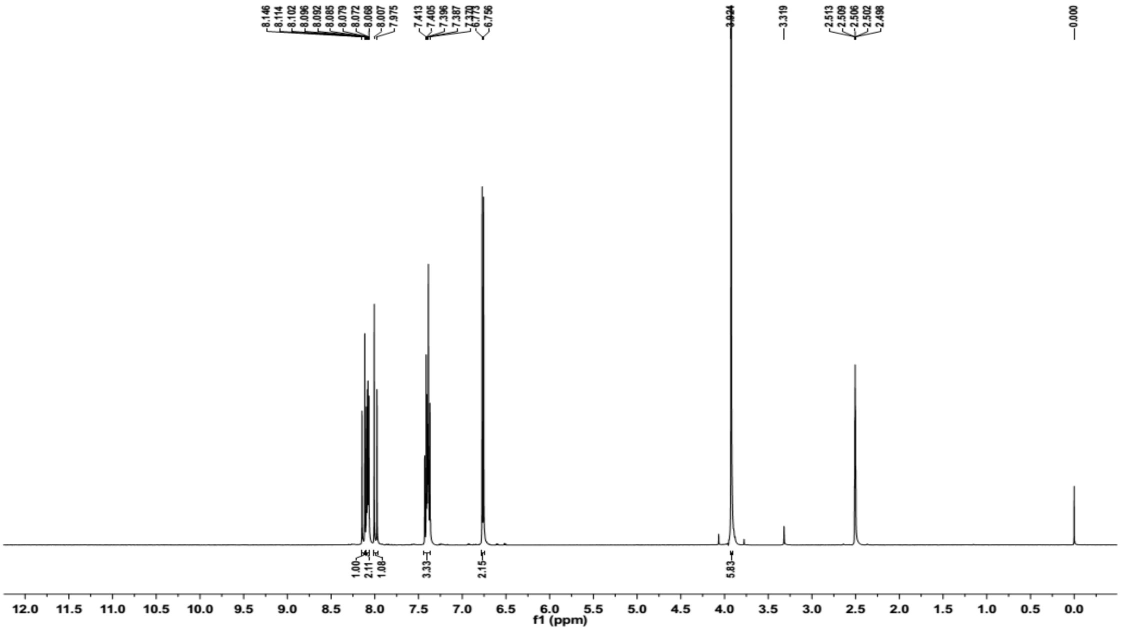

20140919 b33 Bruker AVIIIHD 600 20140919 C13 DMSO D:\\ DATA2014 1

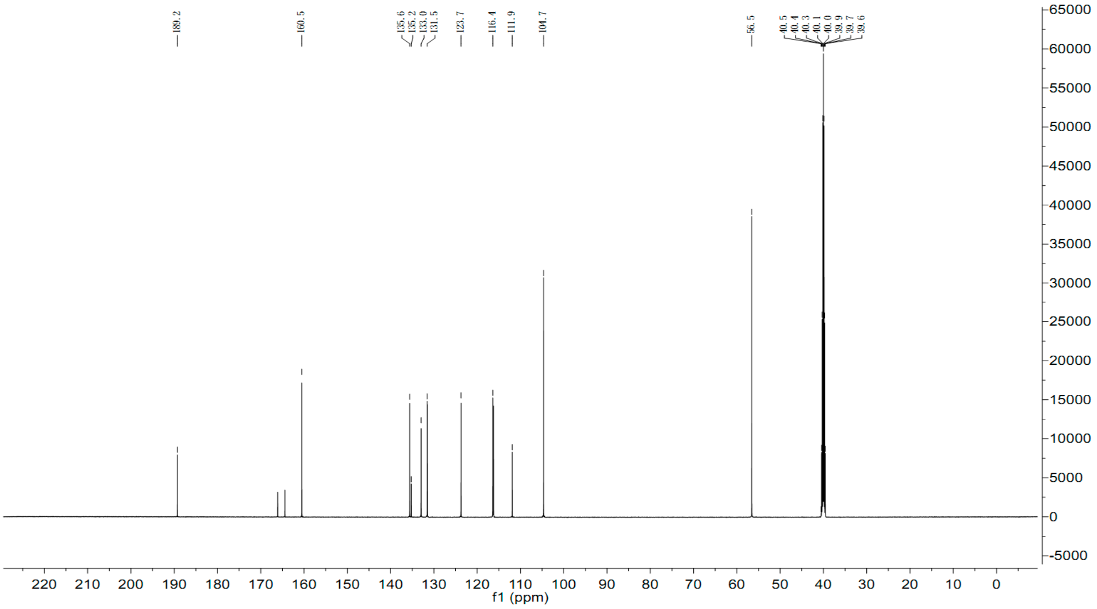

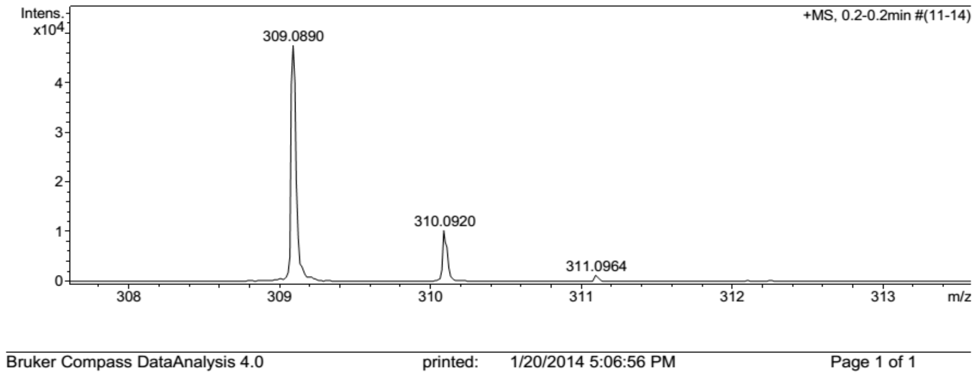

(E)-1-(3-Chlorophenyl)-3-(2,6-dimethoxyphenyl)prop-2-en-1-one (**b34**)

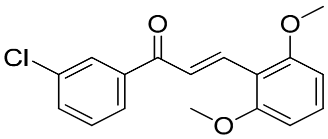

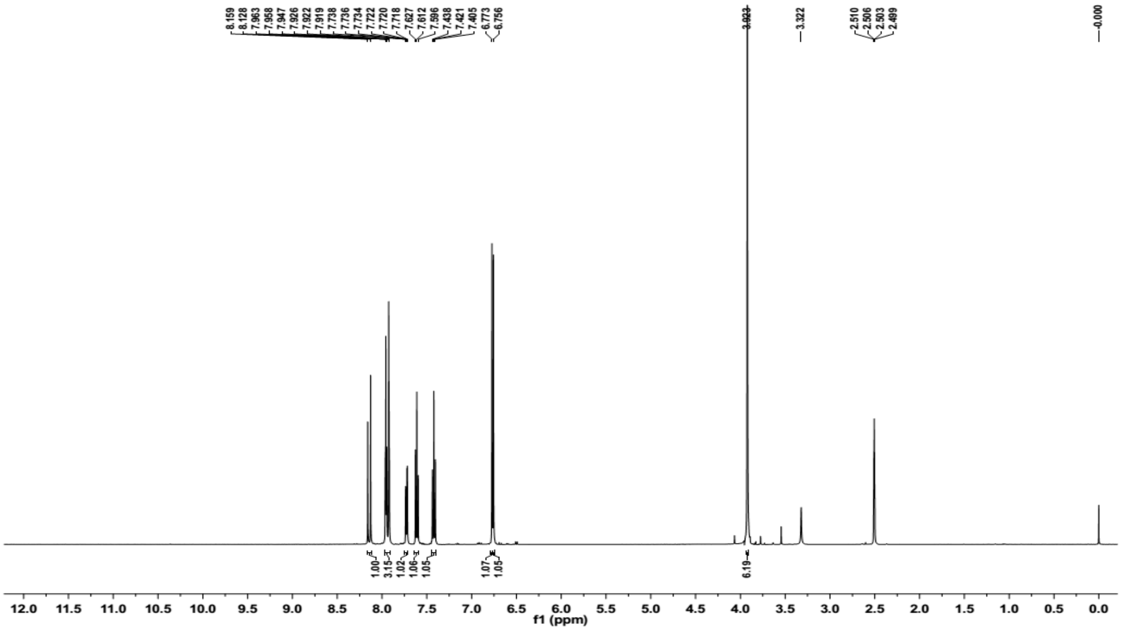

20140919 b34 Bruker AVIIIHD 600 20140919 C13 DMSO D:\\ DATA2014 2

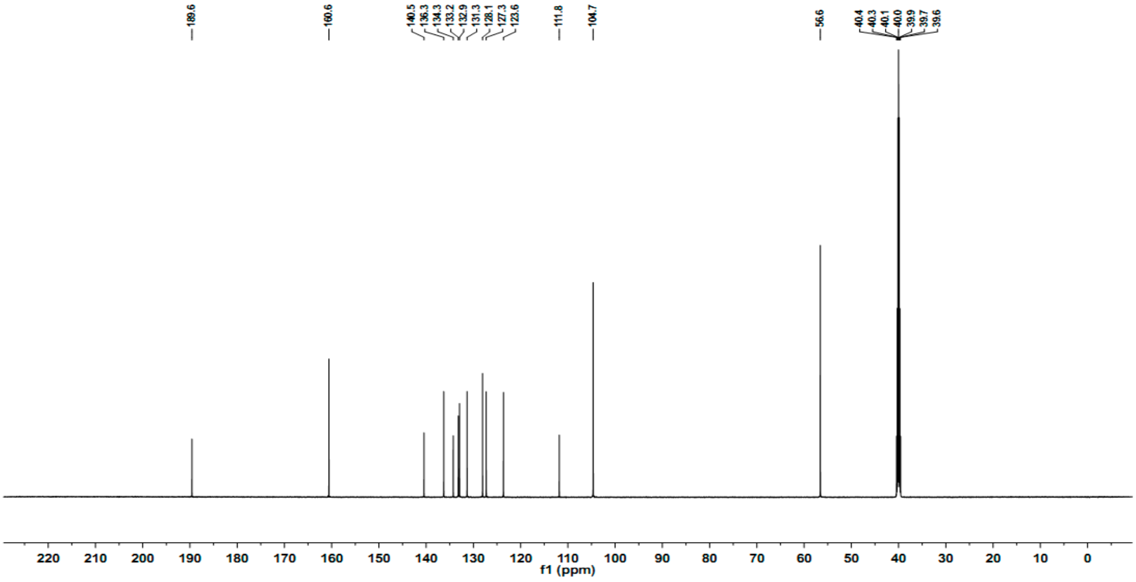

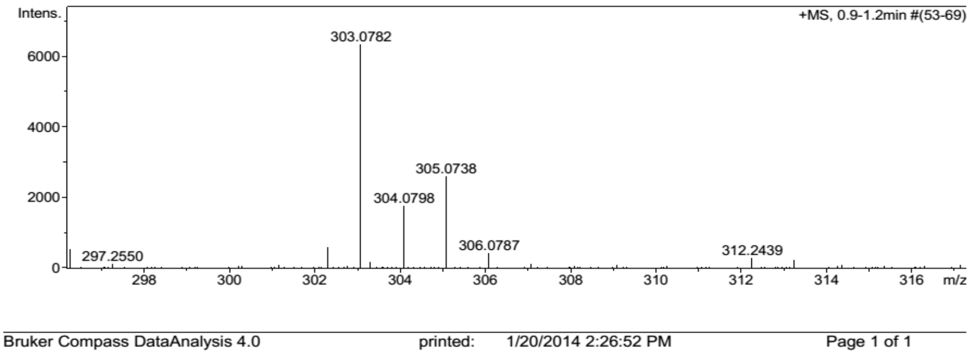

(E)-1-(4-Chlorophenyl)-3-(2,6-dimethoxyphenyl)prop-2-en-1-one (**b35**)

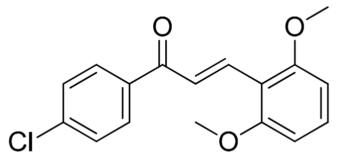

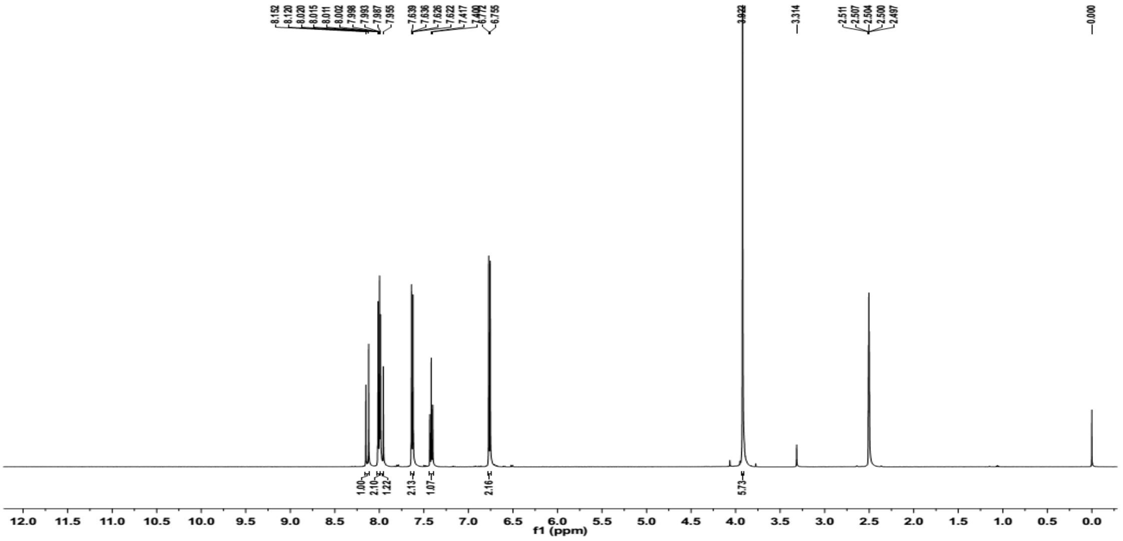

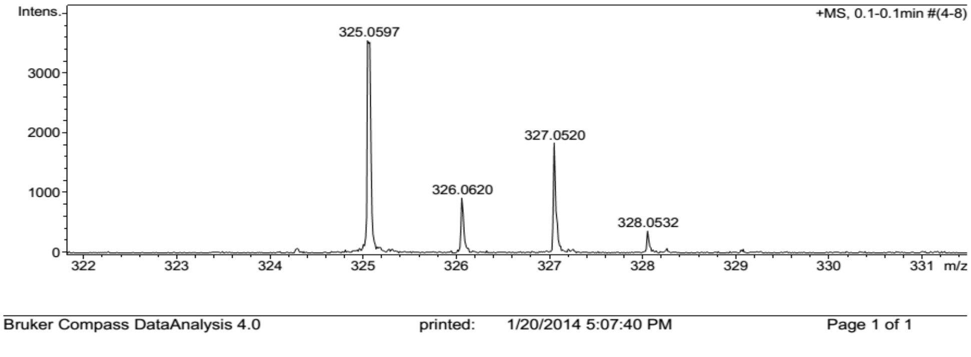

(E)-1-(3-Bromophenyl)-3-(2,6-dimethoxyphenyl)prop-2-en-1-one (**b36**)

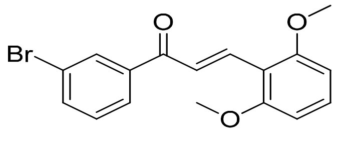

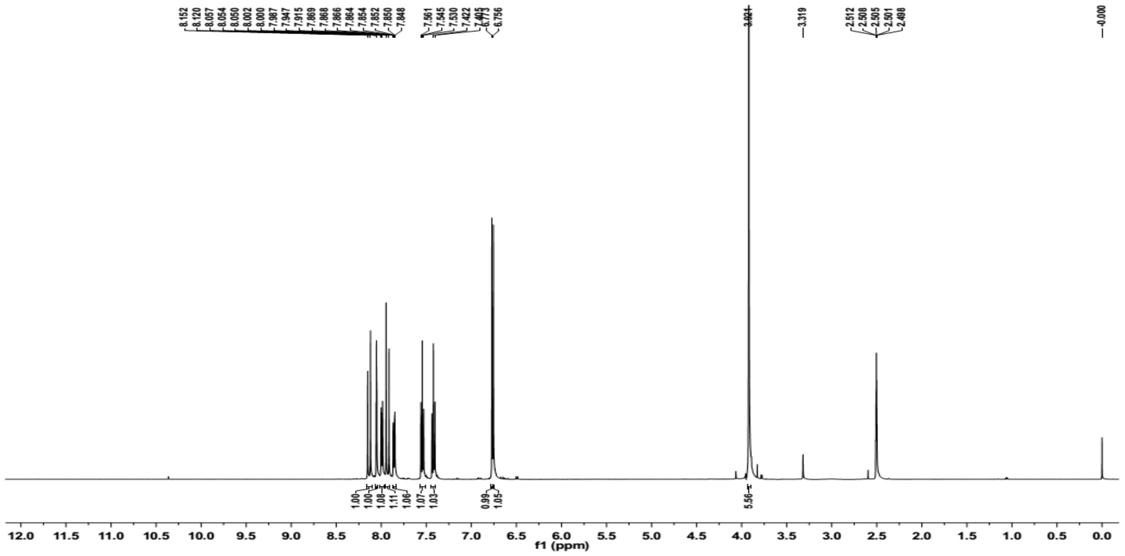

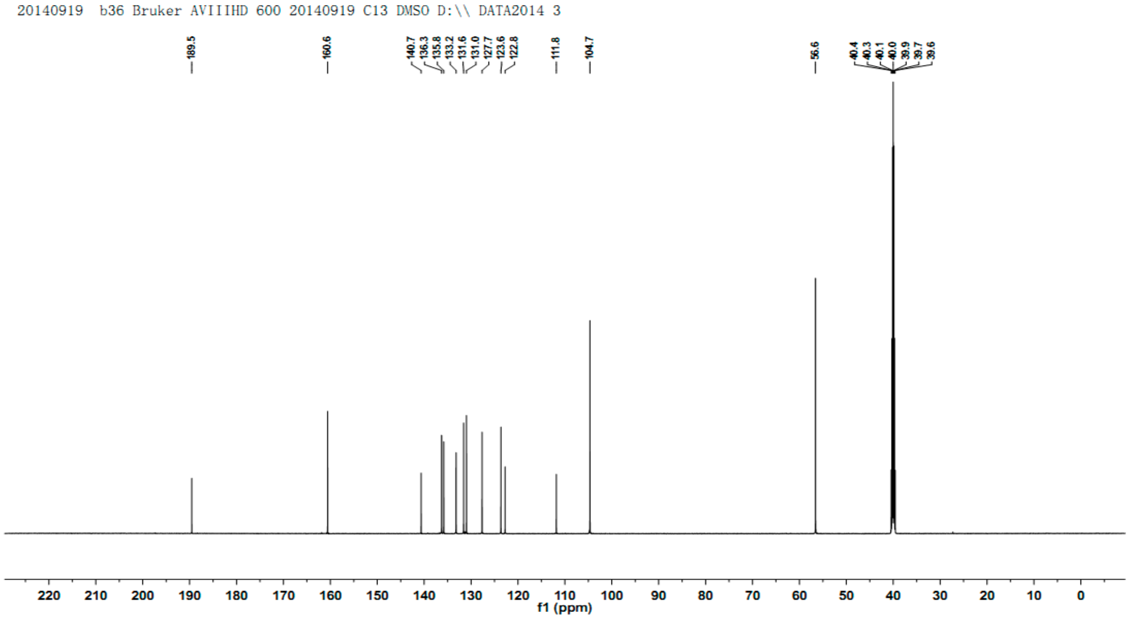

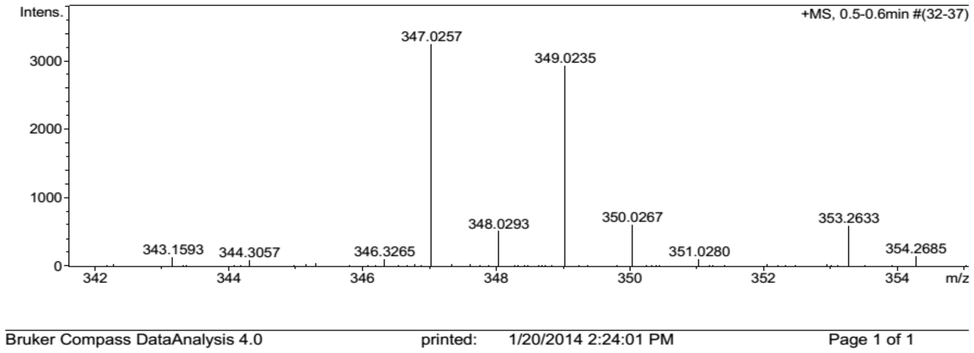

(E)-1-(2,4-Dichlorophenyl)-3-(2,6-dimethoxyphenyl)prop-2-en-1-one (**b37**)

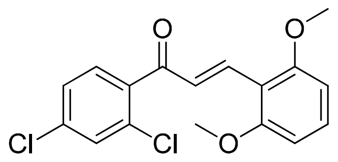

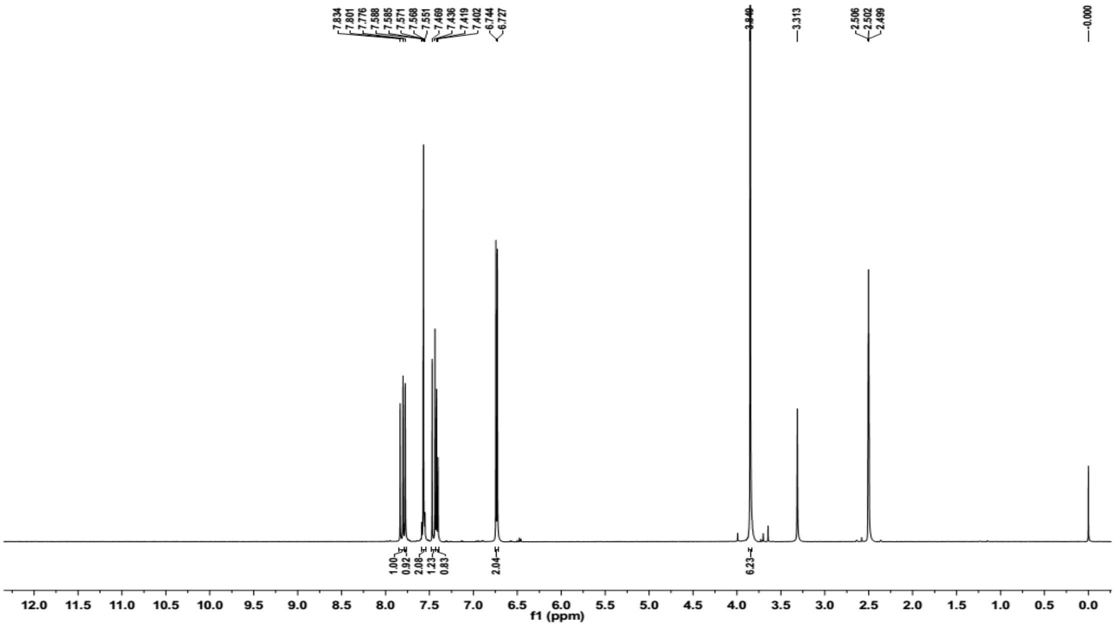

20140919 b37 Bruker AVIIIHD 600 20140919 C13 DMSO D:\\ DATA2014 4

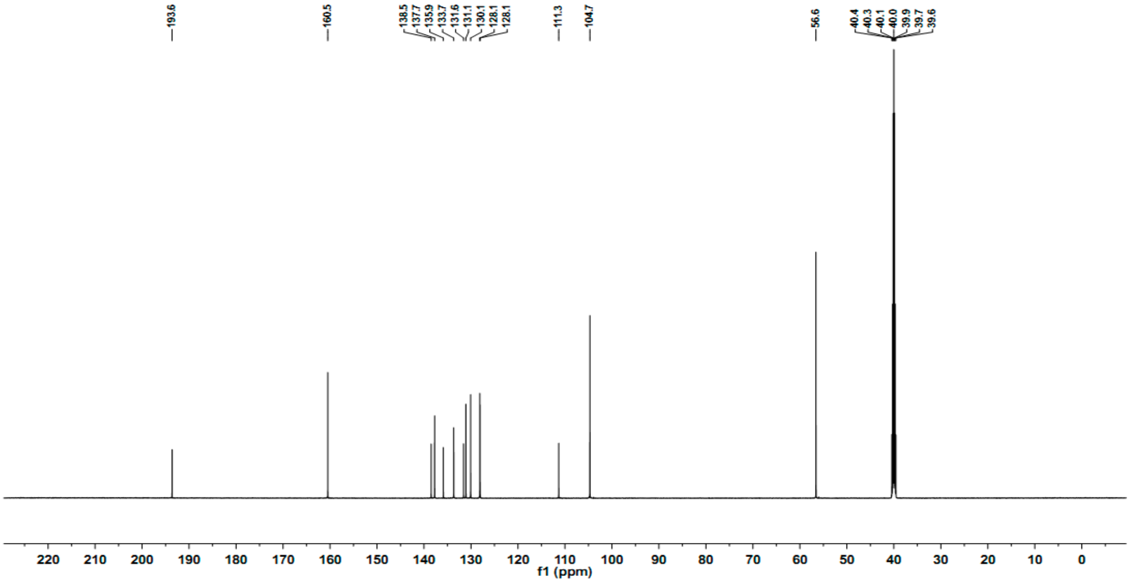

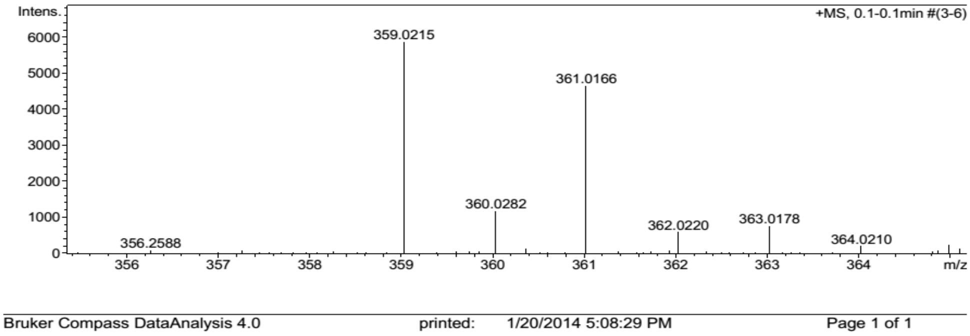

(E)-1-(3,4-Dichlorophenyl)-3-(2,6-dimethoxyphenyl)prop-2-en-1-one (**b38**)

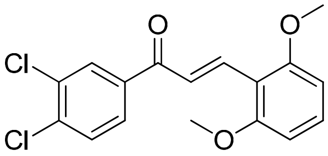

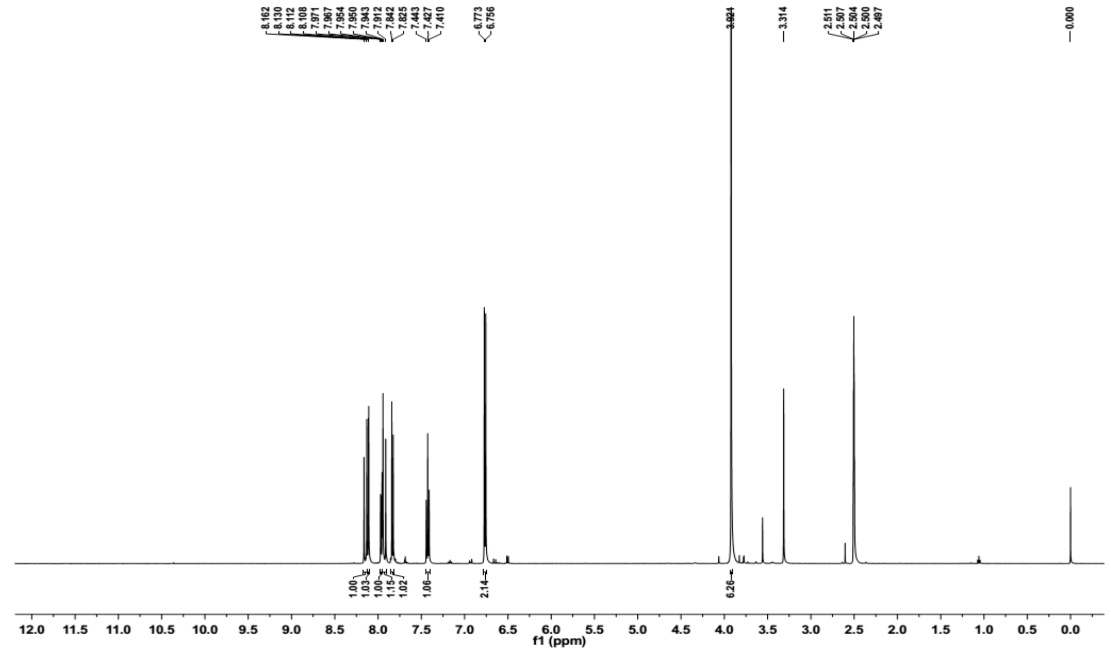

20140919 b38 Bruker AVIIIHD 600 20140919 C13 DMSO D:\\ DATA2014 5

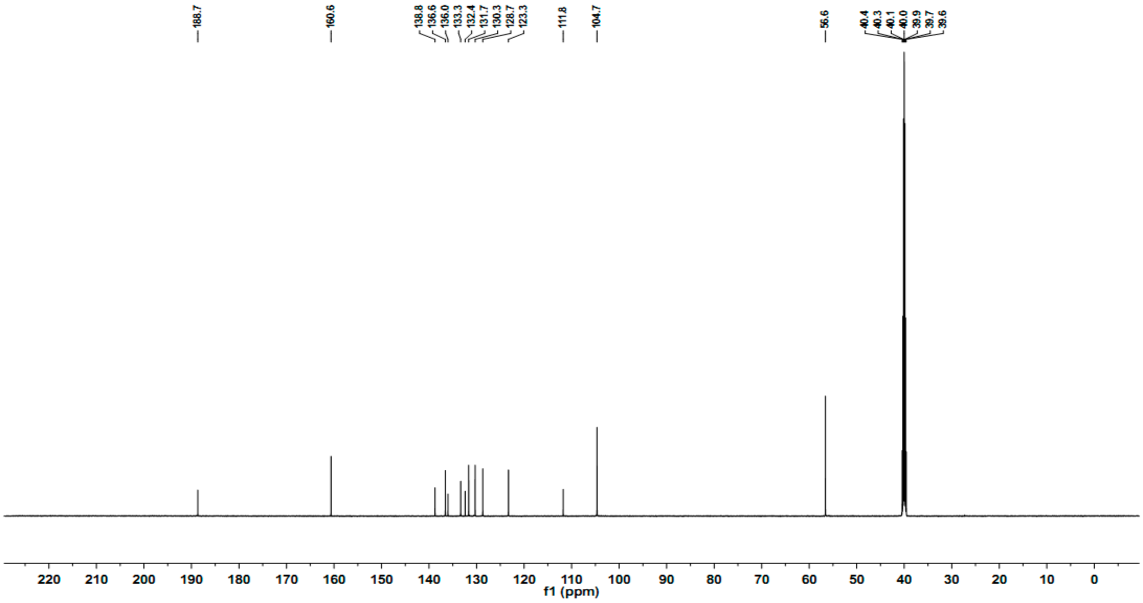

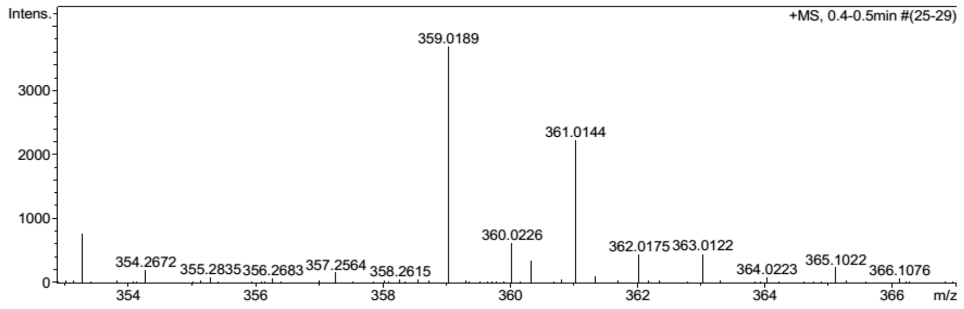

Bruker Compass DataAnalysis 4.0 printed: 1/20/2014 2:30:29 PM Page 1 of 1

(E)-1-(3-Nitrophenyl)-3-(2,6-dimethoxyphenyl)prop-2-en-1-one (**b39**)

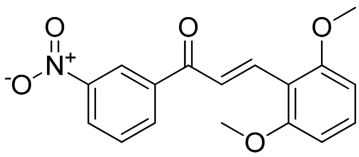

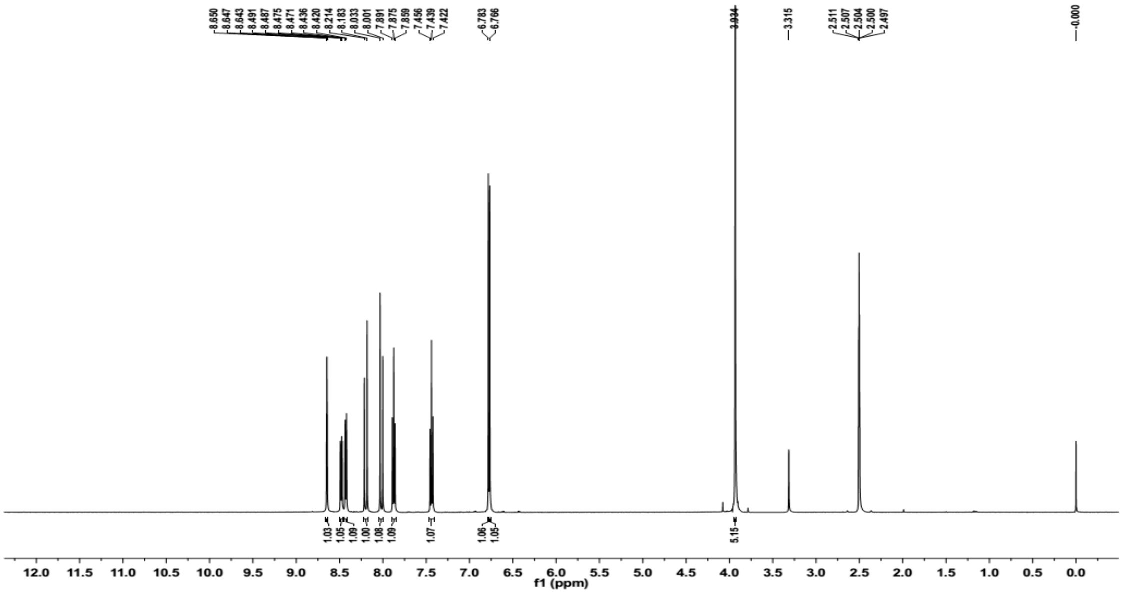

20140919 b39 Bruker AVIIIHD 600 20140919 C13 DMSO D:\\ DATA2014 6

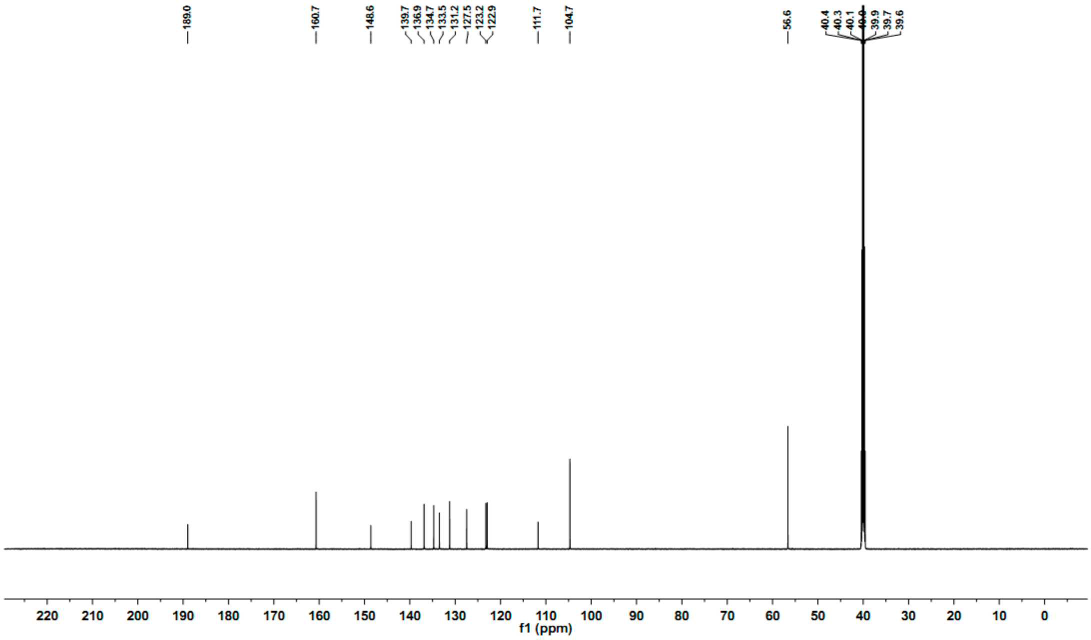

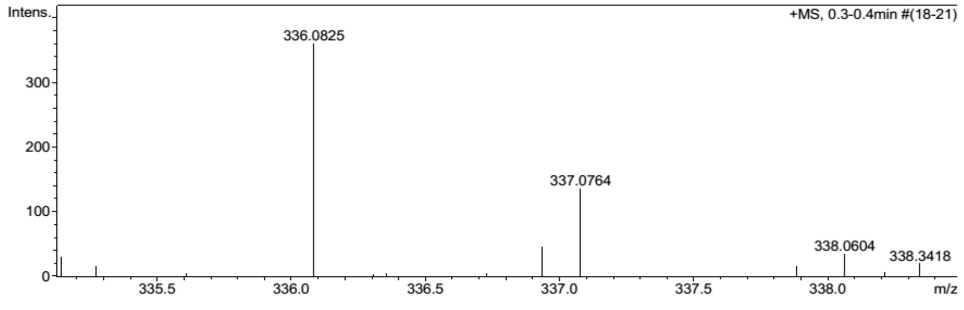

Bruker Compass DataAnalysis 4.0 printed: 1/20/2014 2:36:21 PM Page 1 of 1

(E)-1-(4-Nitrophenyl)-3-(2,6-dimethoxyphenyl)prop-2-en-1-one (**b40**)

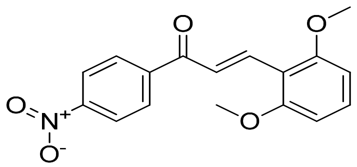

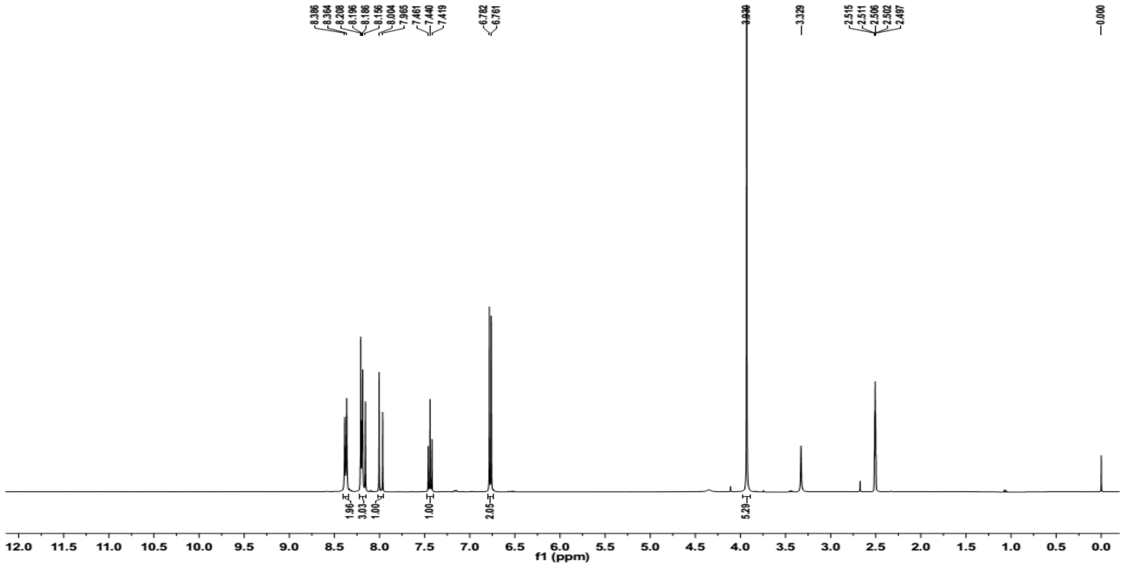

20140919 b40 Bruker AVIIIHD 600 20140919 C13 DMSO D:\\ DATA2014 7

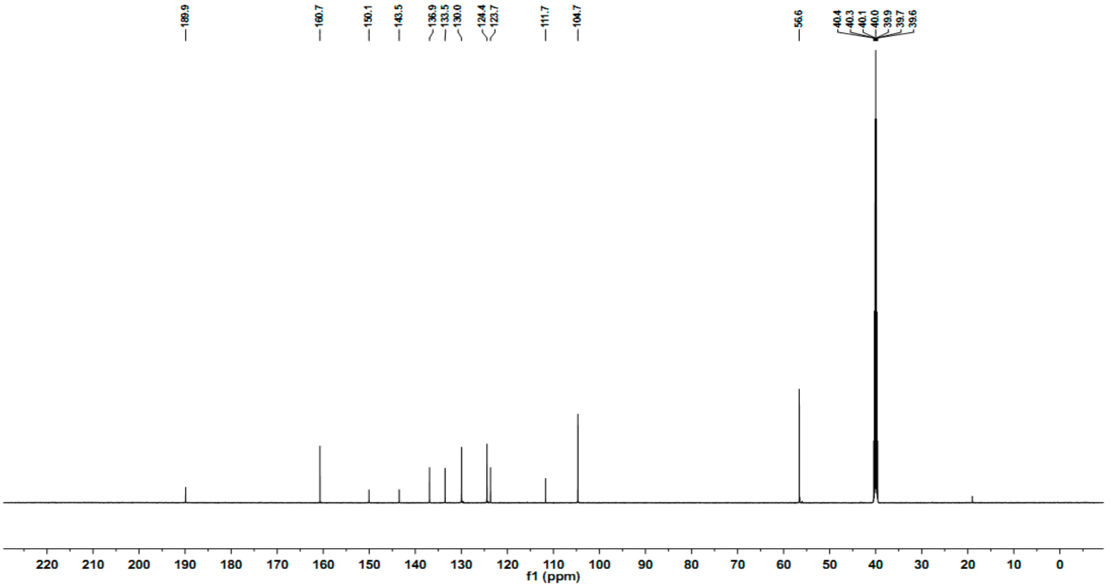

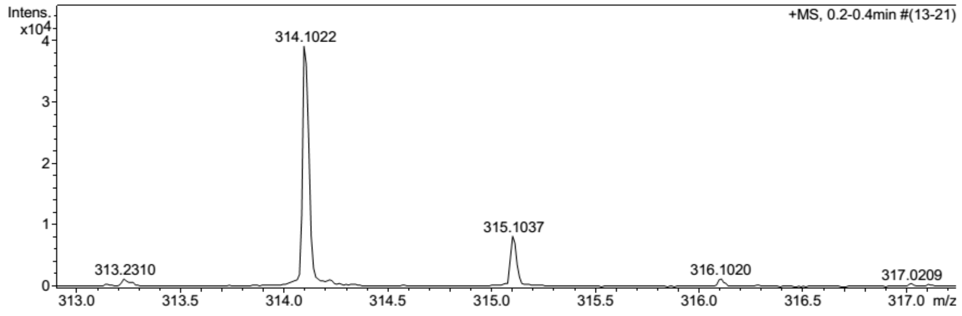

Bruker Compass DataAnalysis 4.0 printed: 1/19/2014 5:07:37 PM Page 1 of 1

(E)-1-(3-(Trifluoromethyl)phenyl)-3-(2,6-dimethoxyphenyl)prop-2-en-1-one (**b41**)

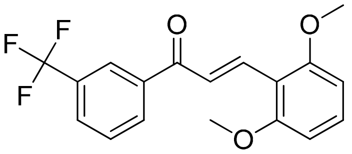

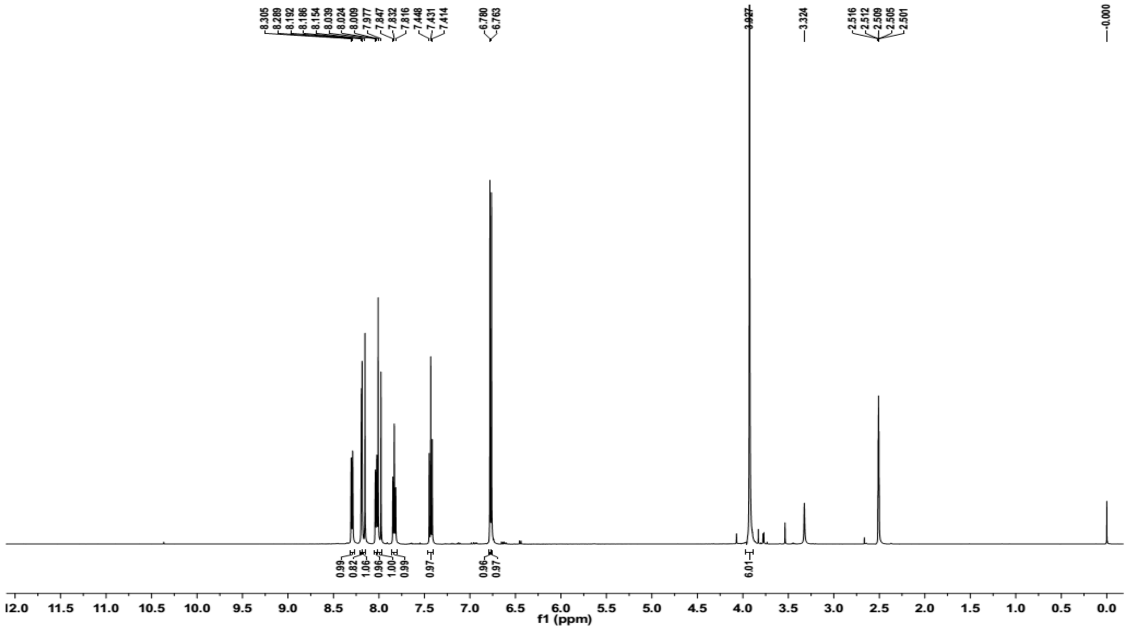

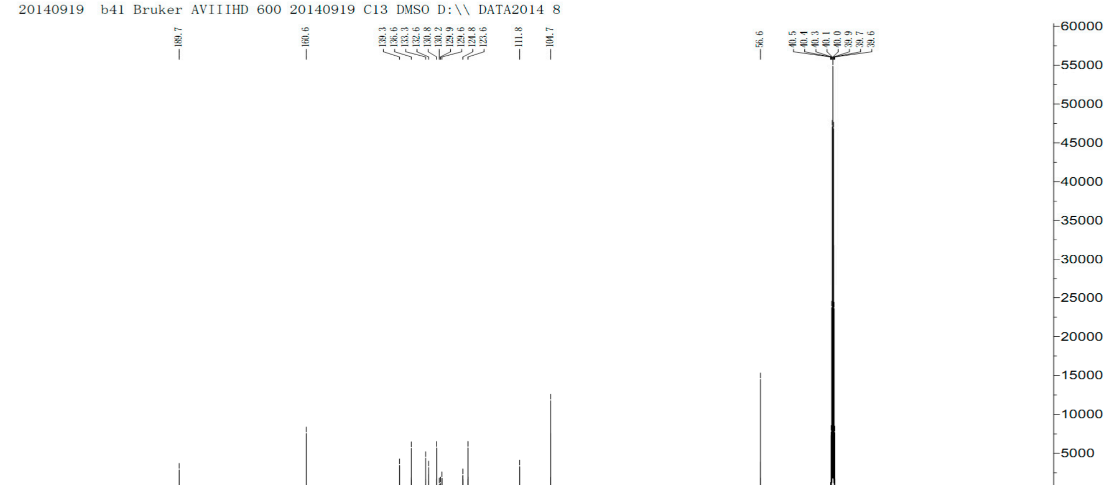

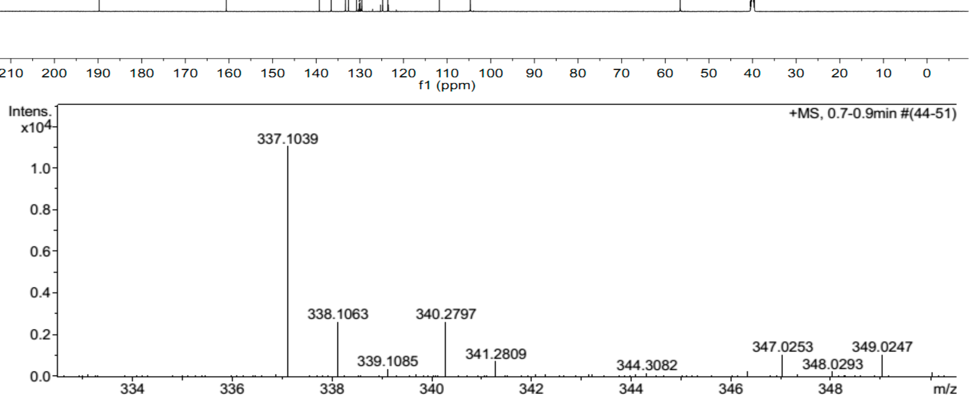

Bruker Compass DataAnalysis 4.0 printed: 1/20/2014 2:25:44 PM Page 1 of 1

(E)-1-(3,5-bis(Trifluoromethyl)phenyl)-3-(2,6-dimethoxyphenyl)prop-2-en-1-one (**b42**)

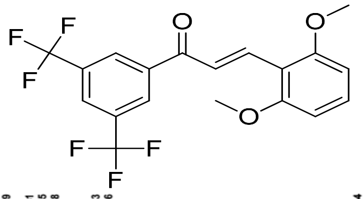

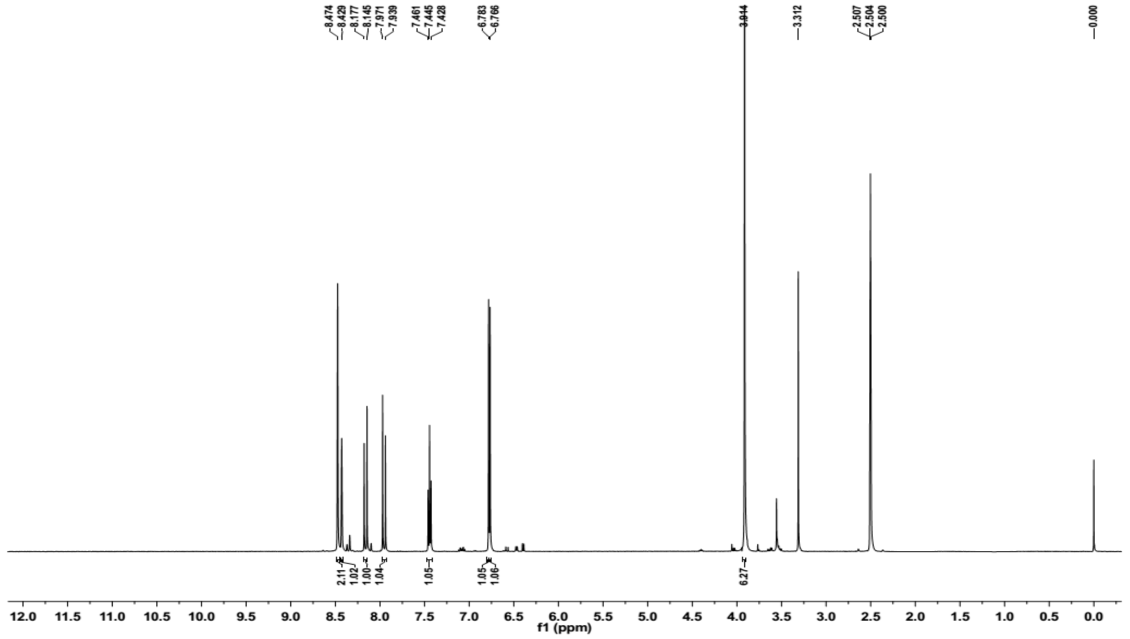

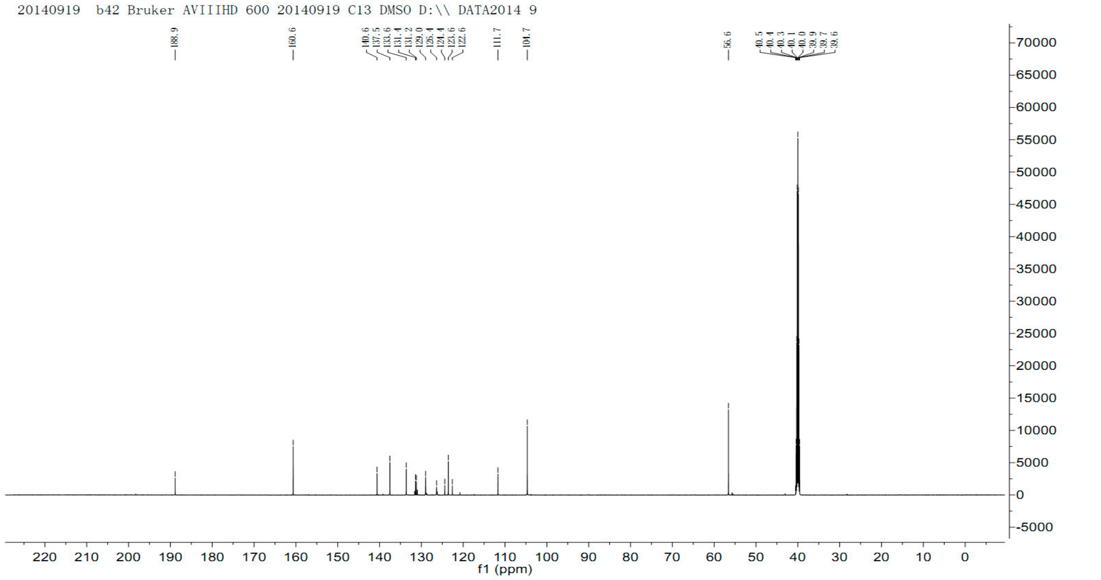

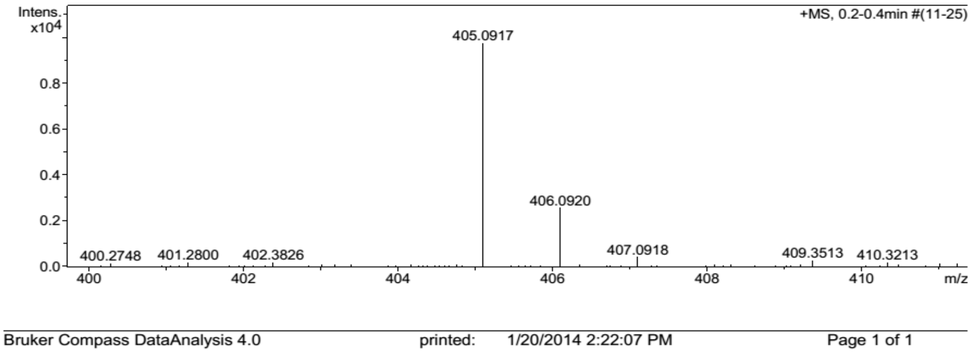

Supplement: Supplementary File 1 [file molecules-19-17256-s001.pdf]
